# KIF15 is essential for USP10-mediated PGK1 deubiquitination during the glycolysis of pancreatic cancer

**DOI:** 10.1038/s41419-023-05679-2

**Published:** 2023-02-17

**Authors:** Gang Quan, Jian Xu, Jie Wang, Xinyuan Liu, Jichuan Xu, Jianxin Jiang

**Affiliations:** 1 Department of Hepatobiliary Surgery, Renmin Hospital of Wuhan University, Wuhan, 430060 China; 2 Department of Hepatobiliary Surgery, Affiliated Hospital of Guizhou Medical University, Guiyang, 550004 China

**Keywords:** Oncogenes, Pancreatic cancer

## Abstract

Glycolysis is the most predominant metabolic reprogramming of pancreatic cancer (PC), the underlying mechanism of which in PC cells remains unclear. In this study, we found for the first time that KIF15 promotes the glycolytic capacity of PC cells and PC tumor growth. Moreover, the expression of KIF15 was negatively correlated with the prognosis of PC patients. The ECAR and OCR measurements indicated that KIF15 knockdown significantly impaired the glycolytic capacity of PC cells. Western blotting demonstrated that the expression of glycolysis molecular markers decreased rapidly after the knockdown of KIF15. Further experiments revealed that KIF15 promoted the stability of PGK1 and its effect on PC cell glycolysis. Interestingly, the overexpression of KIF15 impaired the ubiquitination level of PGK1. To investigate the underlying mechanism by which KIF15 regulates the function of PGK1, we performed mass spectrometry (MS). The MS and Co-IP assay indicated that KIF15 recruited and enhanced the binding between PGK1 and USP10. The ubiquitination assay verified that KIF15 recruited and promoted the effect of USP10 on PGK1, thereby deubiquitinating PGK1. Through the construction of KIF15 truncators, we found that KIF15 is bound to PGK1 and USP10 through its coil2 domain. Together, our study demonstrated for the first time that KIF15 enhances the glycolytic capacity of PC through the recruitment of USP10 and PGK1, and that the KIF15/USP10/PGK1 axis may serve as an effective therapeutic agent for PC.

## Introduction

Pancreatic ductal adenocarcinoma (PDAC) is among the deadliest types cancers of the digestive tract, with a mortality rate of over 95% [1]. Treatment options are limited, and surgery remains the best option. However, patients are usually confirmed in the advanced stage of PDAC, missing the optimal time for surgery [2]. In recent years, the incidence of PDAC has been gradually increasing, despite the significant progression in comprehensive treatment. This is due to the insidious onset and resistance of PDAC to chemotherapy drugs, which led to poor prognosis and low postoperative quality of life. Therefore, it is urgent to explore novel and specific biomarkers, or therapeutic targets to improve the prognosis of PDAC patients [3].

One characteristic of PDAC is its acidic tumor microenvironment, further characterized by excessive fibrosis and hypoxia, and nutrient depletion due to poor vascular function. In order to maintain the rapid proliferation of pancreatic cancer (PC) cells and provide the capacity and material basis for synthetic reactions, PC cells absorb and ferment large amounts of glucose to produce lactic acid, even when oxygen is abundant. This phenomenon is known as the Warburg effect. A growing number of glycolytic enzymes have been reported to be associated with the poor prognosis of PDAC [4, 5], suggesting that interference with glycolytic enzymes is a potential strategy for inhibiting the oncogenic activity of PDAC cells. For example, circular RNA circSLIT2 regulates LDHA expression by sponging miR-510-5p, resulting in the promotion of aerobic glycolysis of PDAC [6]. The combination of PDAC driver mutations and adaptation to this adverse environment prompts extensive metabolic reprogramming of cancer cells to atypical metabolic pathways, and increases reliance on clearance mechanisms such as autophagy, proteasome hydrolysis, and pinocytosis.

Deubiquitination enzyme (DUB) plays an important role in targeting protein degradation and represents an emerging paradigm for cancer therapy [7, 8]. Accumulated evidence reported that DUBs participate in the regulation and stabilization of glycolytic enzymes, leading to the metabolic reprogramming of PDAC cells [9]. For example, USP44 inhibits PDAC cell proliferation and enhances gemcitabine resistance by deubiquitinating FBP1 [9]. FBP1 is one of the key enzymes in gluconeogenesis and negatively regulates aerobic glycolysis. USP25 is positively associated with poor prognosis, and promotes PDAC growth by deubiquitinating and stabilizing the HIF-1α transcription factor. Subsequently, this conserves the high levels of HIF-1α transcriptional activity and upregulates several key enzymes. Eventually, USP25 potentiates aerobic glycolysis and promotes PDAC progression [10]. Therefore, DUBs may be a novel therapeutic target for PDAC treatment.

In our previous study, we found that kinesin 12 (KIF15), a kinesin family member, is a plus-end-directed kinesin that functions to form bipolar spindles. KIF15 is highly expressed in PDAC and promotes PDAC cell proliferation by activating MEK-ERK signaling [11]. However, in this study, we found that KIF15 could enhance aerobic glycolysis and promote PDAC progression. Mechanically, KIF15 serves as a scaffold to recruit USP10 and phosphoglycerate kinase 1 (PGK1), and promote the deubiquitination and stabilization of PGK1 via USP10. PGK1 is a glycolytic enzyme that produces the first ATP by glycolysis and catalyzes the conversion of 1, 3-bisphosphoglycerate to 3-phosphoglycerate [12]. PGK1 is also involved in DNA repair and autophagy. PGK1 is overexpressed in most cancers and suggests a poor prognosis in some cancer types. However, when targeting glycolysis for PDAC, it is important to ensure that the glycolytic enzymes are not impaired by normal glycation or energy metabolism. Therefore, in this study, we focused on the function of KIF15, which may be the key scaffold for USP10 and PGK1 that leads to the metabolic reprogramming and malignant progression of PDAC. This study elucidates KIF15 as a potential novel therapeutic target for PDAC treatment.

## Materials and methods

### Bioinformatics analysis

According to the PC sample resources of the TCGA (The Cancer Genome Atlas) database, the expression levels, prognosis, and pathological grade of the studied genes (KIF15, USP10, and PGK1) in PC and normal tissues were analyzed by GEPIA online (gepia.cancer-pku.cn).

### PC specimens and immunohistochemical analysis

A total of ten PC and adjacent tissue specimens were collected from patients who underwent surgical treatment with the consent of patients in Renmin Hospital at Wuhan University (Wuhan, Hubei, China). All experiments on tissue specimens have been approved by the ethics committee of Renmin Hospital at Wuhan University.

Paraffin-embedded PC tissues fixed with formalin were dewaxed and rehydrated, and incubated with 0.3% H_2_O_2_ at room temperature for 30 min. After the antigen was taken, the slices were blocked with diluted serum for 30 min, and then incubated with primary antibody overnight at 4 °C. Avidin-biotin immunoperoxidase staining was performed, followed by a visual analysis of the samples using ImageScope software (Leica Biosystems, Nussloch, Germany).

### Cell culture and transfection

Two PC cell lines (AsPC-1 and BxPC-3) and a normal human pancreatic ductal epithelial cell line (HPDE) were cultured in RPMI 1640 medium (Hyclone, USA) supplemented with 10% fetal bovine serum (FBS) (Gibco, USA), inside an incubator (37 °C, 5% CO_2_). Another three PC cell lines (SW1990, PANC-1, and MIA PaCa-2) were cultured in DMEM (Hyclone, USA) supplemented with 10% FBS (Gibco, USA). All cell lines were purchased from the American Type Culture Collection (ATCC, Manassas, VA, USA).

### Western blot

RIPA lysate was added to the PANC-1 and MIA PaCa-2 cell lines, and the total protein was extracted after ultrasonic fragmentation. Strict balancing centrifugation (4 °C, 12,000r/15 min) was performed to absorb 5 μl of supernatant per tube for protein quantification. Loading buffer was added to the remaining supernatant for subsequent sample loading.

After sampling loading, SDS-PAGE gel electrophoresis (120 V, 2 h) was performed, followed by membrane transfer (300 mA, 2 h). The PVDF membrane was sealed with defatted milk powder for 30 min after membrane transfer. The bands were then cut accordingly to different index molecular sizes, and different types of primary antibodies were prepared (4 °C, 14–16 h). The membranes were washed with TBST three times, for 10 min each time. After the second antibody was incubated, the membranes were washed with TBST three times, for 10 min each time, and stored at room temperature for 2 h. Finally, chemiluminescence was used to analyze the required bands. The Supplementary table lists the details of all antibodies used in this study.

### Quantitative real-time PCR (Qrt-PCR)

Total RNA was extracted from PC tissues or cells using TRIzol (Invitrogen, USA) based on the manufacturer’s instructions. Reverse transcription of target genes was performed using HiScript®III 1st Strand Cdna Synthesis Kit (+Gdna wipe) (Vazyme, China). The target genes were amplified by Qpcr using ChamQ Universal SYBR Qpcr Master Mix (Vazyme, China). The internal control was GAPDH. The amplification level of the target gene was evaluated by the 2^−ΔΔCt^ method. The Supplementary table lists the details of all PCR primer sequences used in this study.

### Immunofluorescence

First, PC cells were cultured on slides overnight, then fixed with 4% paraformaldehyde for 30 min and infiltrated with 0.1% Triton X-100 (Boster Biological Technology) for 5 min. Later, 5% BSA was added as a blocking buffer and incubated for 1 h. The cells were then incubated with different primary antibodies at 4 °C overnight. CY3-conjugated goat anti-rabbit antibody and fluorescein isothiocyanate (FITC)-conjugated goat anti-mouse secondary antibody were diluted at 1:200 in blocking buffer and treated at 37 °C for 1 h, followed by DAPI staining for 5 min. Lastly, images were obtained by a laser scanning confocal microscope.

### Cell counting kit-8 (CCK-8)

PC cells cultured in 96-well plates were transfected with silenced or control sequences of the target gene (*N* = 1 × 10^4^). To evaluate cell viability, 10% CCK8 working solution (Dojindo, Japan) was prepared and 100 µl was added into each well, followed by incubation at 37 °C for 2 h. The relative cell viability was determined by spectrophotometry at a wavelength of 450 nm.

### Metabolic analyses

Real-time OCR and ECAR were measured using a XF-96 Extracellular Flux Analyzer (Seahorse Bioscience, North Billerica, MA, USA). PC cells of different groups were plated in XF-96 plates (Seahorse Bioscience) and cultured for 12 h in the corresponding medium supplemented with 5% FBS. For OCR measurement, PC cells were cultured in XF media containing a non-buffered medium under basal conditions and individually tested with oligomycin, carbonylcyanide-4-(trifluoromethoxy)-phenylhydrazone (FCCP), and the combination of antimycin and rotenone. ECAR of PC cells was measured in XF media in basal conditions and individually tested with glucose, oligomycin, and 2-DG. The OCR profile represented the mitochondrial respiratory function. The measurement of OCR before the addition of oligomycin represented the basal OCR. The difference between basal OCR rate and oligomycin-induced OCR rate represented the ATP-linked OCR. The difference between the FCCP rate and the combined antimycin and rotenone rate represented the maximal OCR. The ECAR profile represented the glycolytic pathway activation of PC cells. The measurement of ECAR after the addition of glucose represented the basal ECAR. The measurement of ECAR after the addition of oligomycin represented the maximal ECAR. The difference between oligomycin-induced ECAR and 2-DG-induced ECAR represented the glycolytic capacity. Data are expressed as mean ± S.E.M.

#### EdU

For EdU staining, PC cells were treated with 10 μM EdU for 15 min and processed according to the manufacturer’s instructions (Invitrogen, C10356). Cells were fixed with 4% paraformaldehyde and culture medium for 15 min. Then, the cells were washed in PBS, incubated in permeabilization/blocking buffer for 1 h, and stained with DAPI for 5 min. The results were visualized by a fluorescence microscope.

### IHC assay

For IHC staining, the extracted nude tumor, PC, and adjacent normal tissues were fixed for 14 h in formalin, embedded in paraffin, and cut into 5 μm thick sections. These were then incubated with primary antibodies overnight at 4 °C, followed by incubation with secondary antibodies at room temperature for 30 min. Slides were then stained with DAPI, treated with hematoxylin, dehydrated, and mounted for IHC.

### Transwell assay

Matrix intrusion tests were performed in 24-well plates containing Transwell polycarbonate filters (Corning, New York, NY, USA) with an aperture of 8 μm. To the lower chamber, 600 μl medium containing 10% fetal bovine serum (FBS) was added. The cells were placed in serum-free medium (1 × 10^4^ cells/100 μl) in Matrigel (BD Biosciences, San Jose, CA, USA) coated upper chambers and incubated at 37 °C for 24 h. Subsequently, the cells were fixed with 4% paraformaldehyde and stained with 0.5% crystal violet. Finally, the invaded cells were photographed and counted.

### Mass spectrometry

Mass spectrometry analysis was performed according to standard procedures. The precipitations were separated by SDS-PAGE, followed by Coomassie blue staining. The different bands were excised from the gel and analyzed by matrix-assisted laser desorption, ionization time-of-flight, and time-of-flight mass spectrometry.

### Co‐immunoprecipitation (Co‐IP) assay

The whole cell lysates were incubated with primary antibodies against KIF15/USP10/PGK1 or normal IgG at 4 °C overnight, and further incubated with 30 μl protein A/G agarose beads (Abmart, Shanghai, China) for another 2 h. The agarose beads were then washed three times with ice-cold cell lysis buffer, and the immunoprecipitated proteins were analyzed by immunoblotting.

### Ubiquitination assay

For the ubiquitination assay, 293T cells were transfected with Myc-tag Ub and the selected plasmids from this study. The cells were then treated with MG132 to inhibit the function of proteasomes. The cells were immunoprecipitated to tag the PGK1 proteins with Flag-tag antibodies. The immunoprecipitated proteins were then extracted by NP-40 lysis buffer, and bound ubiquitin conjugates were eluted using SDS buffer supplemented with 500 mM Imidazole at 65 °C for 10 min. The samples were analyzed by SDS-PAGE and fluorescence scanning using a Bio-Rad imager.

### PC-bearing nude mice

Our animal experiments were performed in accordance with a protocol approved by the Institutional Animal Care and Use Committee of Wuhan University. Nude mice were purchased from Beiente Biology Corporation. Ten nude mice were randomly separated into two groups for following experiments. The constructs containing KIF15 cDNA sequences or empty vectors were transfected to PANC-1 cells. Nude mice (Jackson Laboratory) were injected subcutaneously in the flanks with the transfected PANC-1 cells suspended in Matrigel. The mice were monitored for 13 weeks after injection and sacrificed thereafter.

### Statistical analysis

All experiments in this study were repeated three times. The statistical software GraphPad Prism 6 was used for statistical analysis. Sample statistical values were presented as mean ± standard deviation (SD). Student’s *t* test was used for the comparison of means of two independent samples, and analysis of variance (ANOVA/Fisher test) was used for the comparison of means of multiple samples. *p* value ≤ 0.05 indicated that the difference was statistically significant.

## Results

### KIF15 was highly expressed in PC cancer

**Fig. 1:**
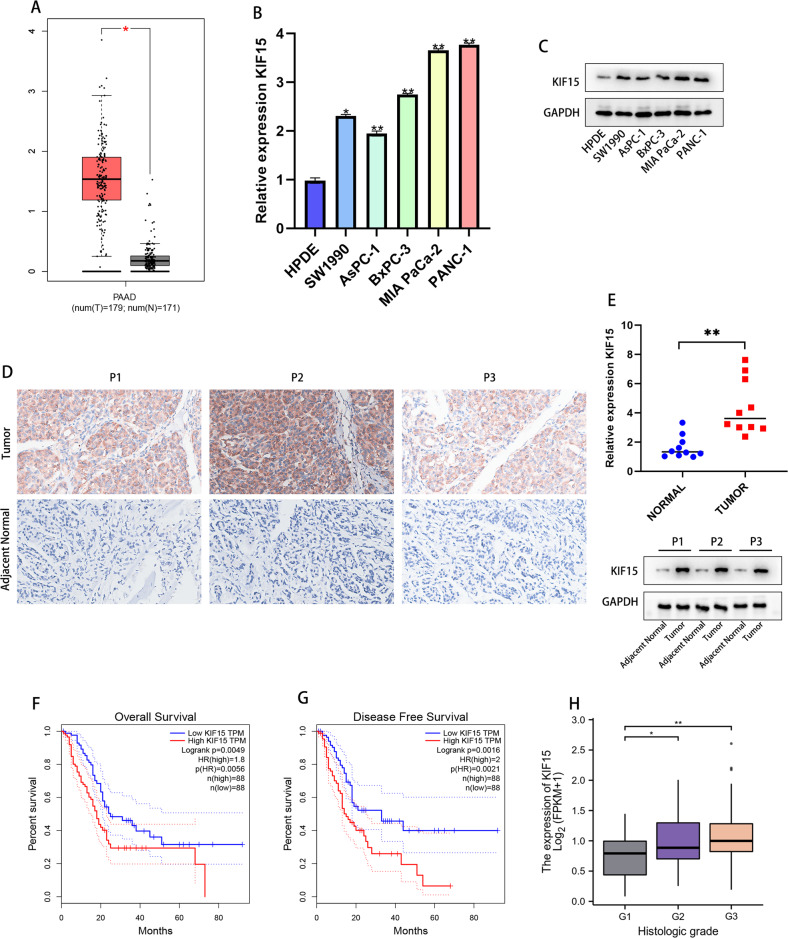
KIF15 was highly expressed in PC cancer. **A** The expression of KIF15 in TCGA-PAAD dataset. **B** The relative expression of KIF15 mRNA in PC cells and normal pancreatic cells. **C** The KIF15 protein expression in PC cells and normal pancreatic cells. **D** The expression of KIF15 mRNA in PC tissues. *n* = 10. **E** The western blotting and IHC staining of KIF15 in PC patient tissues. *n* = 10. **F** The overall survival of patient in KIF15-low or KIF15-high expression group. **G** The disease-free survival of PC. *n* = 88. **H** The correlation between KIF15 and PC histologic grade. *n* = 88.

Based on The Cancer Genome Atlas (TCGA) database, we compared the expression of KIF15 in normal pancreatic tissues and PC tissues, and the results showed that KIF15 expression elevated in PC tissues than in normal ones (Fig. 1A). In line with the bioinformatics prediction, the PCR and western blot results demonstrated that PC cells exhibited a risen expression of KIF15 than that in normal pancreatic ductal cells (Fig. 1B, C). Moreover, after collecting 10 pairs of matched PC and adjacent normal pancreatic tissue samples, we conducted PCR and IHC experiments to test the expression of KIF15. The results indicated that the expression of KIF15 upregulated in tumor tissues than in the adjacent normal tissues (Fig. 1D, E). The clinical data from TCAG-PDAC showed that a High KIF15 mRNA expression significantly decreases overall survival and disease-free survival in PC patients (*n* = 88) (Fig. 1F, G). Moreover, found that patients with higher KIF15 was accompanied with higher histologic grade (Fig. 1H).

### KIF15 promoted the migration, invasion and glycolytic capacity of PC cells

**Fig. 2:**
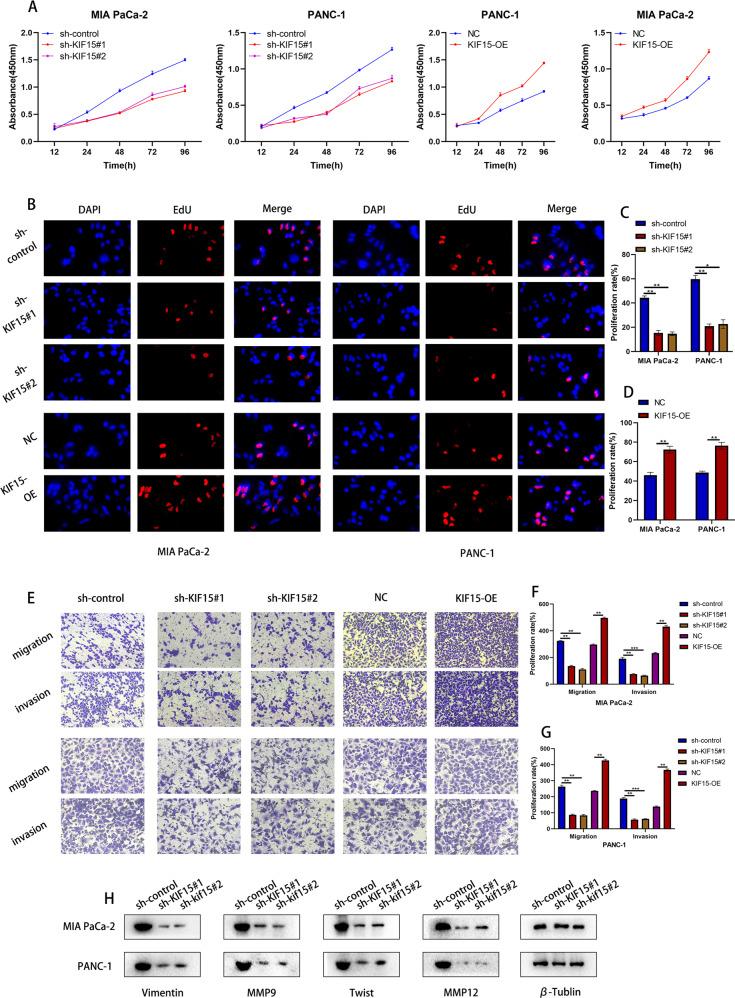
KIF15 promoted the malignant phenotype of PC cells. **A** The proliferation rate of PC cells measured by CCK-8 assay. **B** The EdU assay of PC cells transfected with different plasmids. **C** The quantification of EdU assay in PC cells transfected with sh-control plasmid or KIF15 shRNA plasmids. **D** The quantification of EdU assay in PC cells transfected with negative control plasmid or KIF15 overexpression plasmids. **E** The transwell assay pf PC cells transfected with different plasmids. **F** The quantification of transwell migration assay in PC cells. **G** The quantification of transwell invasion assay in different group of PC cells. **H** The western blot assay in PC cells transfected with corresponding plasmids.

Our previous research has elucidated the role of KIF15 in the proliferation of PC. To further elucidate the effect of KIF15 on the malignant biological behavior of PC, we performed corresponding function experiments in PC cells transfected with KIF15 overexpression or knockdown plasmids. CCK-8 assay indicated that after transfection of KIF15 shRNA, PC cells exhibited a decreased proliferation rate, while the opposite results were observed in KIF15 overexpression group (Fig. 2A, B). Similar results were shown in our EdU assay, compared with the control group, knockdown of KIF15 significantly decreased the EdU-positive rate on PC cells, whereas the overexpression of KIF15 led to opposite results in PC cells (Fig. 2C–E). The indicated results implied that KIF15 promoted the proliferation of PC cells. Next, we conducted the Transwell assay to investigate the influence of KIF15 on cell migration and invasion. Our results demonstrated that KIF15 knockdown impeded the migration and invasion of PC cells, whereas the KIF15 overexpression group of PC cells showed enhanced ability of migration and invasion (Fig. 2F–H).

**Fig. 3:**
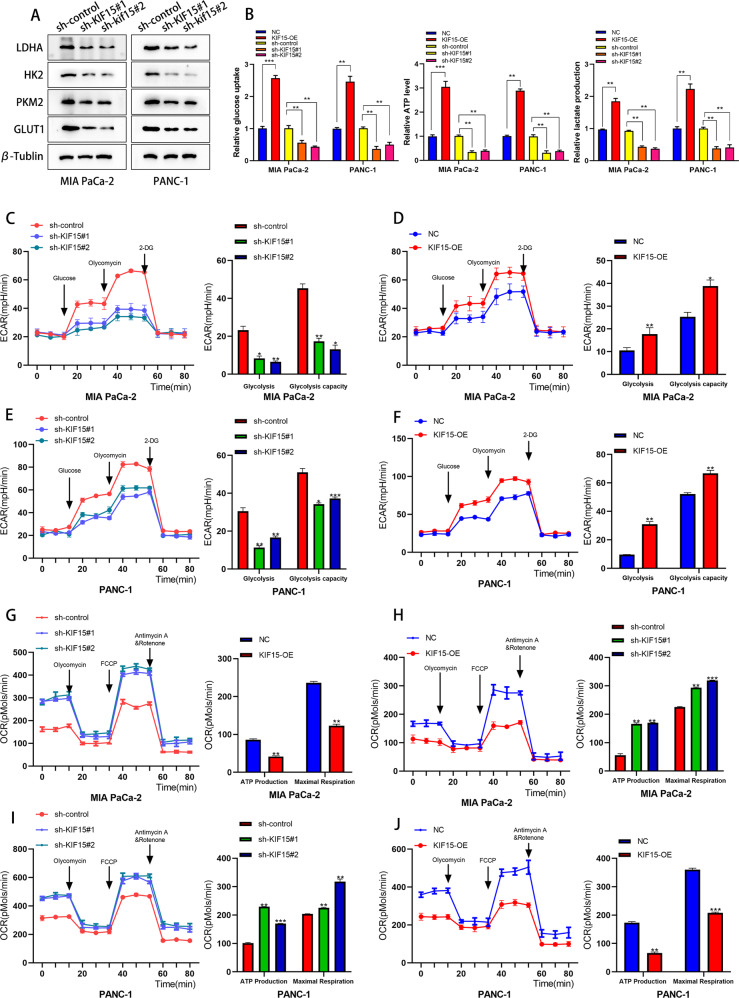
KIF15 promoted the glycolysis of PC cells. **A** The Western blot assay of glycolysis markers in PC cells. **B** The glucose uptake, ATP and lactate production assay of PC cells. **C** The ECAR assay and quantification of Mia PaCa-2 cells transfected with KIF15 shRNA. **D** The ECAR assay and quantification of Mia PaCa-2 cells transfected with KIF15 overexpression plasmids. **E** The ECAR assay and quantification of PANC-1 cells transfected with KIF15 shRNA. **F** The ECAR assay and quantification of PANC-1 cells transfected with overexpression plasmids. **G** The OCR assay and quantification of Mia PaCa-2 cells transfected with KIF15 shRNA. **H** The OCR assay and quantification of Mia PaCa-2 cells transfected with KIF15 overexpression plasmids. **I** The OCR assay and quantification of PANC-1 cells transfected with KIF15 shRNA. **J** The OCR assay and quantification of PANC-1 cells transfected with KIF15 overexpression plasmids.

Considering that glycolysis supported the metabolism of PC cells and enhanced the malignant phenotype of tumor cells, we performed a western blot to detect the alteration of glycolysis-related molecular markers in PC cells. Interestingly, the expression of LDHA, HK2, PKM2, and GLUT1 decreased rapidly after KIF15 knockdown (Fig. 3A). The above results indicated that KIF15 may be involved in the process of glycolysis of PC cells. Further, by detecting the cellular energy metabolism of PC cells we found the glucose uptake, ATP production, and lactate production decreased after KIF15 knockdown and increased after KIF15 overexpression (Fig. 3B). Moreover, the ECAR and OCR measurements demonstrated that KIF15 knockdown significantly reduced both baseline ECAR and maximum glycolytic capacity but increased the basal ATP production and maximal respiration of PC cells. While KIF15 overexpression had the opposite effect on above parameters of PC cells (Fig. 3C–J).

### KIF15 interacted with PGK1

**Fig. 4:**
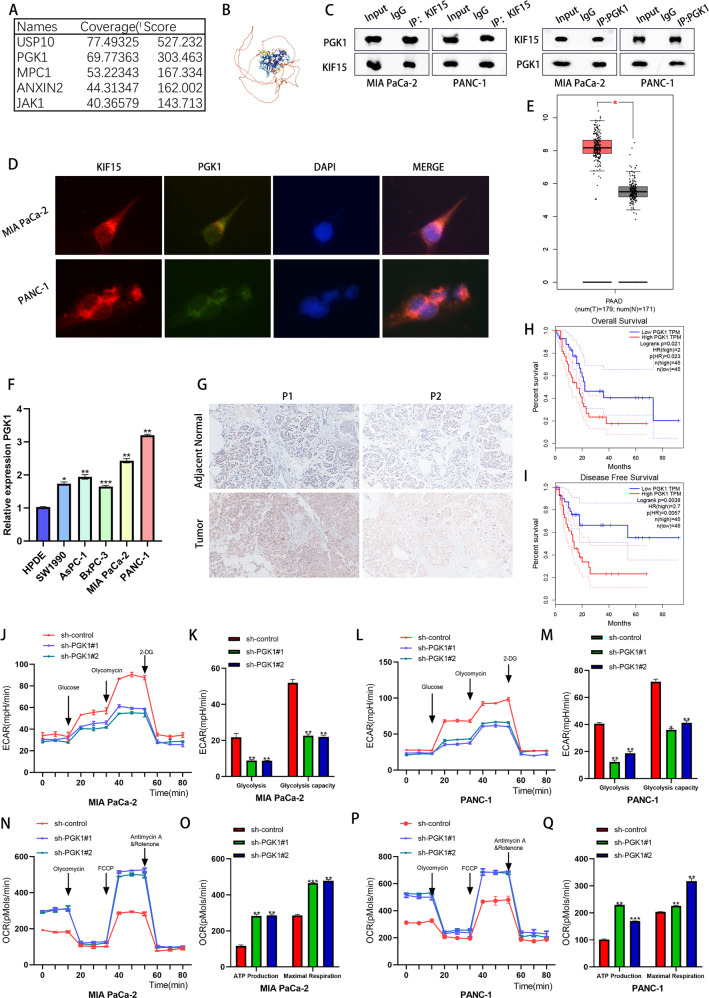
KIF15 interacted with PGK1. **A** The mass spectrum showed the possible binding proteins of KIF15. **B** The predicted construction of PGK1 by bioinformatic tools (https://alphafold.ebi.ac.uk/). **C** The reciprocal co‐immunoprecipitation assay between PGK1 and KIF15. **D** The immunofluorescence between KIF15 (red) and PGK1 (green). **E** The expression of PGK1 in TCGA-PAAD dataset. **F** The relative expression of PGK1 mRNA in PC cells and normal pancreatic cells. **G** The IHC staining of PGK1 in PC patient tissues. *n* = 10. **H** The overall survival of patient in PGK1-low or PGK1-high expression group. *n* = 45. **I** The disease-free survival of PC patient in PGK1-low or PGK1-high expression group. *n* = 45. **J**, **K** The ECAR assay and quantification of Mia PaCa-2 cells transfected with PGK1 shRNA. **L**, **M** The ECAR assay and quantification of PANC-1 cells transfected with PGK1 shRNA. **N**, **O** The OCR assay and quantification of Mia PaCa-2 cells transfected with PGK1 shRNA. **P**, **Q** The OCR assay and quantification of PANC-1 cells transfected with PGK1 overexpression plasmids.

Given that KIF15 is a member of the kinesin family, involved in the transportation of cancer related proteins, we speculate that KIF15 regulated the metabolic reprogramming of PC cells by interacting with other regulators. Hence, we performed mass spectrometry to screen the potential interactor of KIF15 in PC cells. Our data identified phosphoglycerate kinase 1 (PGK1) as the most probable candidate (Fig. 4A). We then conducted the Co-IP assay to confirm the binding between PGK1 and KIF15, which indicated that KIF15 and PGK1 interacted with each other. Similar results were observed in the IF assay which demonstrated that KIF15 colocalized with PGK1 in the cytoplasm of PC cells (Fig. 4D). By analyzing TCGA-PAAD data, we found that PGK1 was highly expressed in PC tissue. Further PCR experiments and IHC assay also demonstrated that PGK1 expression was significantly higher in PC cells or PC tissues than that in normal cells or tissues (Fig. 4E–G). The clinical data of TCGA indicated that PC patients with higher expression of PGK1 were accompanied by poorer overall survival or disease-free survival (Fig. 4H, I). For metabolic analysis, the ECAR was measured as a surrogate of glycolytic flux and the OCR was measured as the representation of glucose consumption of PC cells. As our results showed, PGK1 knockdown in PC cells significantly reduced both baseline ECAR and maximum glycolytic capacity but increased basal ATP production and maximal respiration (Fig. 4J–Q). Moreover, the transfection of PGK1 shRNA also decreased the glucose uptake, ATP production and lactate production of PC cells (Fig. S1A–C). The indicated results hinted that KIF15 regulated the glycolysis of PC cells in a PGK1-dependent manner.

### KIF15 mediated the function of PGK1 at the post-translational level

**Fig. 5:**
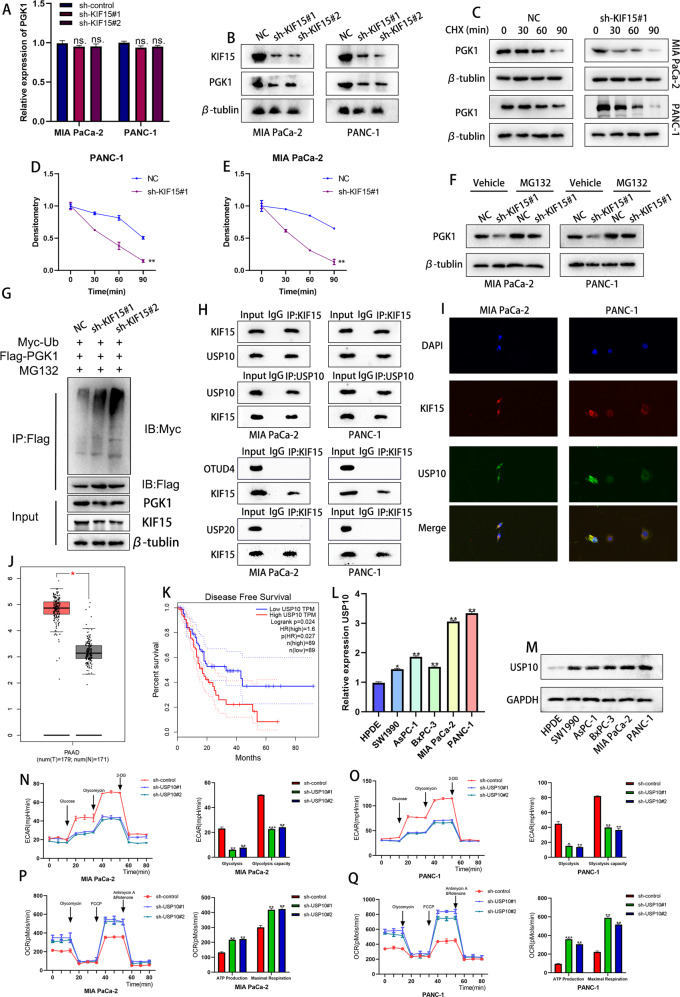
KIF15 medicated the function of PGK1 at the post-translational level. **A** The alteration of PGK1 mRNA after the knockdown of KIF15. **B** The western blot assay showed the protein alteration of PGK1 after the knockdown of KIF15. **C** The half-life of PGK1 protein was tested by western blot after the PC cells treated with CHX. **D** The quantification of PGK1 degradation in PANC-1 cells. **E** The quantification of PGK1 degradation in MIA PaCa-2 cells. **F** The protein level of PGK1 in PC cells treated with MG132. **G** The ubiquitination level of PGK1 measured by western blot assay. **H** The co‐immunoprecipitation assay between KIF15 and three deubiquitinase. **I** The immunofluorescence between KIF15 (red) and USP10 (green). **J** The expression of USP10 in TCGA-PAAD dataset. **K** The disease-free survival of PC patient in USP10-low or USP10-high expression group. *n* = 89. **L** The relative expression of USP10 mRNA in PC cells and normal pancreatic cells. **M** The expression of USP10 protein in PC cells and normal pancreatic cells. **N** The ECAR assay and quantification of Mia PaCa-2 cells transfected with USP10 shRNA. **O** The ECAR assay and quantification of PANC-1 cells transfected with USP10 shRNA. **P** The OCR assay and quantification of Mia PaCa-2 cells transfected with USP10 shRNA. **Q** The OCR assay and quantification of PANC-1 cells transfected with USP10 shRNA.

To figure out the exact mechanism by which KIF15 and PGK1 influence the progression of PC cells, we performed PCR and western blot assay to observe the alteration of PGK1 on PC cells transfected with KIF15 shRNA. The data indicated that KIF15 knockdown inhibited the expression of PGK1 protein, while no alteration was observed for PGK1 mRNA (Fig. 5A, B). After treating the PC cells with cycloheximide (CHX) which blocked the protein synthesis to study the effect of KIF15 on PGK1 protein turnover, our data showed that deficiency of KIF15 in PC cells significantly reduced the half-life of PGK1 protein (Fig. 5C–E). On the other hand, the decrease in PGK1 protein caused by KIF15 knockdown was reversed by treatment of the proteasome inhibitor, MG132 (Fig. 5F). Together, our data indicated that KIF15 enhances the stability of PGK1 and prevents its degradation by proteasomes.

Given that KIF15 inhibits the proteasomal degradation of PGK1 and considering that ubiquitination was the main process of proteasome-mediated protein degradation, we hypothesized KIF15 may be involved in the ubiquitination modification of PGK1. So we performed the ubiquitination assay to verify the effect of KIF15 on PGK1 post-translational modification. After the transfection of KIF15 shRNA, the ubiquitination level of PGK1 increased rapidly (Fig. 5G). This indicated that KIF15 promoted the deubiquitination of PGK1 in PC cells, which was interesting as KIF15 was not a member of the deubiquitinating enzyme family. Hence, we hypothesized that KIF15 might regulate the interaction between PGK1 and its deubiquitinating enzyme. Based on our hypothesis, we screened two deubiquitinating enzymes, OTUD1 and USP10 through mass spectroscopy. To determine the exact deubiquitinating enzyme of PGK1, we performed Co-IP experiments, and the results indicated that KIF15 was bound to USP10 but had no interaction with OUTD4 protein (Fig. 5H). This was consistent with the results obtained from our IF assay which showed that KIF15 and USP10 were colocalized in the cytoplasm of PC cells (Fig. 5I). Taken together, our results suggested that USP10 interacts with KIF15 and may be involved in the ubiquitination of PGK1. Further we also conducted the Co-IP experiment and confirmed that PGK1 could interacted with USP10 protein (Fig. 2A).

We next used the bioinformatic tools to analyze the potential role of USP10 in PC progression. Data from TCGA-PAAD indicated that the expression of USP10 was higher in PC tissues than in normal pancreatic tissues, and PC patients with higher expression of USP10 were accompanied by shorter disease-free survival (Fig. 5J, K). Our PCR and western blot result also indicated that PC cells exhibited higher expression of USP10 than normal pancreatic cells (Fig. 5L, M).

To verify the effect of USP10 in the glycolysis of PC cells, we measured the ECAR and OCR of PC cells transfected with USP10 shRNA. Results showed knockdown of USP10 significantly decreased the baseline ECAR and maximum glycolytic capacity but increased basal ATP production and maximal respiration of PC cells (Fig. 5K–P). Moreover, the transfection of USP10 shRNA also decreased the glucose uptake, ATP production and lactate production of PC cells (Fig. 3A–C). The above results confirmed that USP10 acted as a driver of glycolysis in PC.

### KIF15 recruited USP10 to mediate PGK1 deubiquitination

**Fig. 6:**
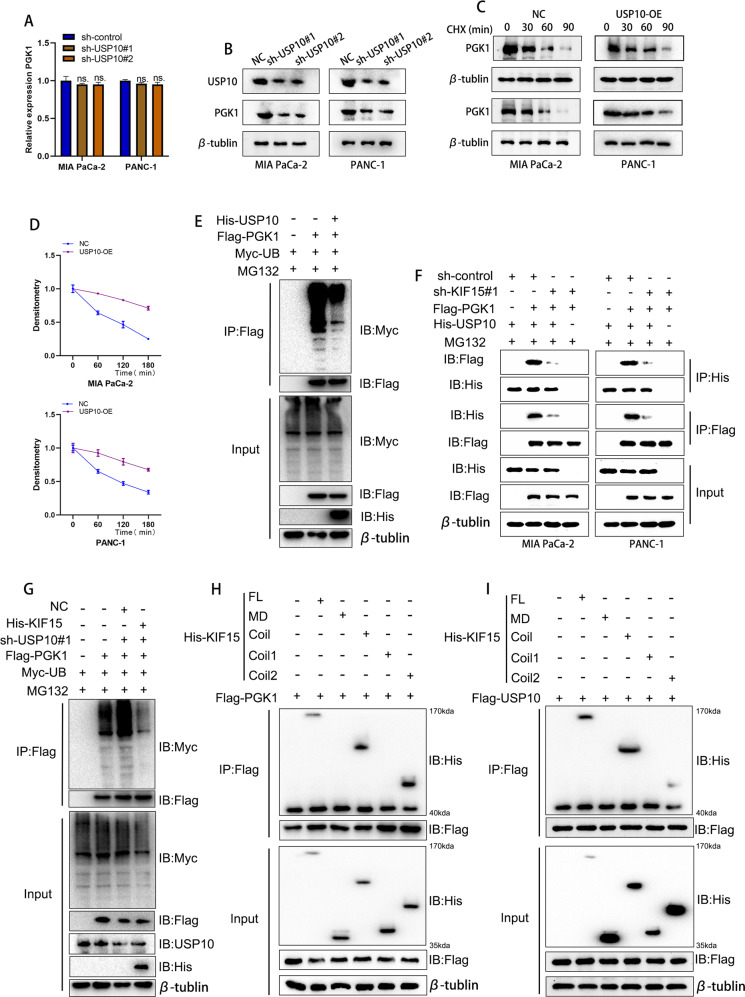
KIF15 recruited USP10 to mediate the deubiquitination of PGK1. **A** The alteration of PGK1 mRNA after the knockdown of USP10. **B** The western blot assay showed the protein alteration of PGK1 after the knockdown of USP10. **C** The half-life of PGK1 protein was tested by western blot after the PC cells treated with CHX. **D** The quantification of PGK1 degradation in PC cells. **E** The ubiquitination level of PGK1 measured by western blot assay. **F** The co‐immunoprecipitation assay between PGK1 and USP10. **G** The ubiquitination level of PGK1 measured by western blot assay. **H**, **I** After construction of indicated KIF15 truncation, the western blot assay showed the exact binding domain of KIF15 responsible for the binding of USP10 and PGK1.

Though our results confirmed that USP10 interacted with PGK1, it was unclear whether USP10 mediated the deubiquitination of PGK1. We conducted PCR and western blot to observe the effect of USP10 on the expression of PGK1 mRNA and protein. The results indicated that USP10 knockdown inhibited the expression of PGK1 protein but did not affect the expression of PGK1 mRNA (Fig. 6A, B). When treated with CHX, PC cells exhibited increased stability of PGK1 protein after the transfection of overexpressed USP10 plasmids (Fig. 6C–E). The ubiquitination assay showed that USP10 overexpression inhibited the ubiquitination level of PGK1 (Fig. 6F). Taken together, our results indicated that USP10 deubiquitinated PGK1 and increased its protein stability.

To determine the role of KIF15 in USP10-mediated PGK1 deubiquitination, we performed a reciprocal Co-IP assay and found that both USP10 and PGK1 could bind to each other, but the binding was prevented in KIF15 knockdown group (Fig. 6G). This indicated that KIF15 functions as a scaffold for the biding between USP10 and PGK1. Taken together, our data suggested that KIF15 recruited both USP10 and PGK1 and enhanced the interaction between USP10 and PGK1.

Furthermore, we conducted the ubiquitination assay to investigate the role of KIF15 in USP10-mediated ubiquitination of PGK1. Our results indicated that the knockdown of USP10 increased the ubiquitination level of PGK1, while KIF15 overexpression reversed the effect (Fig. 6H). Moreover, to determine the exact binding domain between KIF15 and both USP10 and PGK1, we transfected truncated KIF15 plasmids into 293T cells and performed the Co-IP assay. The results showed that the coil2 domain of KIF15 protein was the binding domain between KIF15 and both PGK1 and USP10 (Fig. 6I, J).

### USP10 and PGK1 were essential for KIF15 to mediate the malignant phenotype and the metabolism of PC

**Fig. 7:**
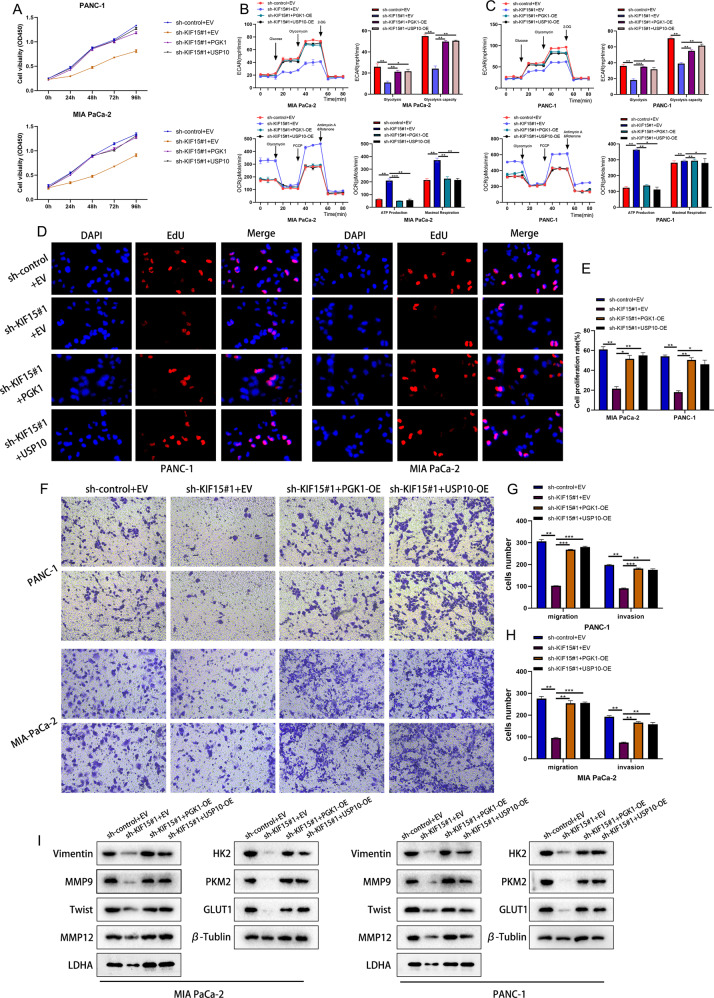
USP10 and PGK1 were essential for KIF15 to mediate the malignant phenotype of PC. **A** The proliferation rate of PC cells measured by CCK-8 assay. **B** The ECAR assay and quantification of PC cells transfected with indicated plasmids. **C** The OCR assay and quantification of PC cells transfected with indicated plasmids. **D** The EdU assay of PC cells transfected with indicated plasmids. **E** The quantification of EdU assay. **F** The Transwell experiment of PC cells transfected with indicated plasmids. **G**, **H** The quantification of Transwell assay. **I** The western blot assay of glycolysis, proliferation and invasion markers in PC cells.

To verify the effect of PGK1 and USP10 in the KIF15-mediated malignant phenotype of PC cells, we conducted rescue experiments. Our CCK-8 and EdU assay indicated that KIF15 knockdown impaired the proliferation ability of PC cells, whereas the overexpression of PGK1 or USP10 reversed the effect (Fig. 7A, D, E). The ECAR and OCR assay indicated that knockdown of KIF15 significantly impaired the glycolytic capacity of PC cells, but increased ATP production and maximal respiration. Overexpression of USP10 and PGK1 rescued the effects of KIF15 (Fig. 7B, C). Meanwhile, the effect of KIF15 on the glucose uptake, ATP production and lactate production of PC cells was rescued by co-transfection of KIF15 with PGK1 or USP10 (Fig. 4A–C). Similarly, the Transwell assay indicated that KIF15 knockdown inhibited the migration and invasion of PC cells, while the overexpression of PGK1 or USP10 reversed the effect (Fig. 7F–H). For western blot, the expression of molecular markers of proliferation, invasion, and glycolysis decreased in PC cells after the transfection of KIF15 shRNA, while overexpression of USP10 or PGK1 restored their expression (Fig. 7I). All things considered, our rescue experiments verified that KIF15 regulates the progression of PC cells through its interaction with USP10 and PGK1.

### KIF15 accelerated tumor growth of PC in vivo

**Fig. 8:**
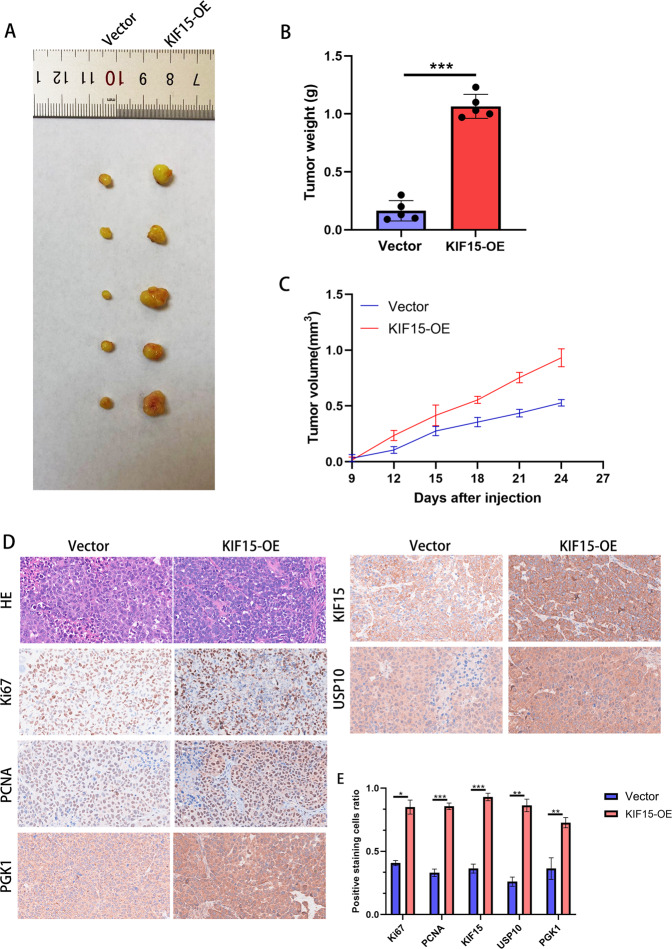
KIF15 enhanced the tumor growth of PC in vivo. **A** The xenografted PC tumors in nude mice transfected with KIF15 overexpression plasmids. *n* = 5. **B** The tumor weight quantification. **C** The tumor volume quantification. **D** The IHC staining of HE, Ki67, PCNA, PGK1, KIF15 and USP10 in xenografted tumors. **E** The quantification of IHC staining. *n* = 5.

For in vivo experiments, we transfected PC cells with KIF15 overexpression lentivirus and injected different group of PC cells into PC-bearing nude mice. The results indicated that KIF15 overexpression accelerated tumor growth in PC-bearing nude mice (Fig. 8A). Nude mice injected with overexpressed KIF15 PC cells increased tumor size (Fig. 8B, C). Moreover, IHC results indicated that higher expressions of KIF15 protein in PC tumors were accompanied by higher expressions of Ki67, PCNA, PGK1, and USP10 (Fig. 8D, E). The above results showed KIF15 promoted the tumor growth of PC and promoted the expression of PGK1 in nude mice.

## Discussion

Accumulated evidence suggests that elevated expression of KIF15 is positively associated with poor prognosis in multiple malignancies [13–17]. Aberrant expression of KIF15 promotes cancer cell proliferation and migration and inhibits cell apoptosis [18]. In our previous study, we confirmed that KIF15 is highly expressed in PDAC, and high expressions of KIF15 are negatively correlated with overall survival [11]. In this study, we measured the expression of KIF15 based on TCGA database and confirmed that KIF15 was highly expressed in PDAC. In addition, we discovered for the first time that KIF15 promoted PDAC proliferation and migration. In brief, KIF15 could enhance the aerobic glycolysis of PDAC by providing a scaffold for USP10 and PGK1 binding. The protein of PGK1 was deubiquitinated and stabilized by USP10, thus increasing the expression of PGK1 and promoting aerobic glycolysis of PDAC.

The interaction between KIF15 and DUBs was also confirmed in other cancers. For instance, Ge et al. reported that KIF15 overexpression reduced USP15-mediated DEK degradation, leading to leiomyosarcoma cell growth [19]. Furthermore, KIF15 was reported to directly bind to the N-terminus of androgen receptor (AR)/AR-V7 and prevent the degradation of AR/AR-V7 proteins, which contributed to enzalutamide resistance in prostate cancer [20]. Mechanically, KIF15 increased the association between USP14 and AR/AR-V7, resulting in increased protein stabilization of AR/AR-V7 by USP14-mediated deubiquitination. Meanwhile, the transcriptionally active AR induced KIF15 expression, which generated a positive feedback system that promotes drug resistance and cancer progression [20]. In this study, we first reported that KIF15 could promote aerobic glycolysis. We then performed IP-MS and Co-IP to confirm the interaction between KIF15 and both USP10 and PGK1. Moreover, USP10 could bind to PGK1, where the interaction between PGK1 and USP10 was decreased by KIF15 deficiency. USP10 increased the protein stabilization of PGK1 via deubiquitination. However, downregulated expression of KIF15 significantly suppressed the deubiquitination of PGK1 by targeting USP10. Later, it was shown that KIF15 promoted cell proliferation and migration by upregulating PGK1, which promoted aerobic glycolysis and metabolic reprogramming. PDAC progression via the proposed mechanism is independent of the activation of ERK signaling, which was previously investigated [11].

Glycolysis is critical for cancer cells to maintain their intrinsic characteristics, such as a high rate of biosynthesis, taxing energy requirements, tumor initiation, cell invasion, angiogenesis, and metastasis to distant organs [12]. Notably, PGK1 could play various roles in PDAC progression, and also as a biomarker for poor prognosis [21–23]. Liang et al. reported that transforming growth factor-β/SMAD4 can inhibit the glycolytic enzyme, PGK1. SMAD4 loss induces the upregulation of PGK1 in PDAC, which enhances glycolysis and aggressive tumor behavior [24]. Jiang et al. found that PGK1 is the target gene of NFAT5, which could upregulate PGK1 expression via transcriptional regulation, induce glycolysis, and subsequently contribute to PDAC progression [25]. In this study, we found that USP10 could upregulate PGK1 protein expression. The coil2 domain of KIF15 was also identified as the binding domain with PGK1 and USP10, and the interaction between them increased the deubiquitination of PGK1 by targeting USP10. It was also shown that upregulated PGK1 promoted glycolysis and PDAC progression. Interestingly, PGK1 also performed other functions in tumors, independent of its role as a glycolytic enzyme [26, 27]. For example, PGK1 could regulate the β-catenin expression and participate in the regulation of tumor metastasis [28]. Nuclear PGK1 inhibited the expression of E-cadherin by functioning as a transcription factor. E-cadherin induced the redistribution of cytoplasmic β-catenin to the cell membrane, thereby increasing tumor cell-cell adhesion [24, 29], suggesting that PGK1 is involved in the activation of Wnt signaling and tumor metastasis.

In summary, we found that KIF15 promoted PDAC aerobic glycolysis, proliferation, and migration. Mechanistically, KIF15 functioned as a scaffold for the direct binding of USP10 and PGK1, which enhances the interaction between USP10 and PGK1. This promoted USP10-mediated deubiquitination of PGK1. Overall, this study suggested that KIF15 might be an effective therapeutic target for the treatment of glycolysis in PDAC.

## Supplementary information

aj-checklist

Supplementray file

SUPPLEMENTAL FigureS1

SUPPLEMENTAL FigureS2

SUPPLEMENTAL FigureS3

SUPPLEMENTAL FigureS4

Figure legend for supplementary figures

Original Data File

## Data Availability

All data generated and analyzed during this study are included in this published article are available on request.
