# Supplementary material for: KIF15 is essential for USP10-mediated PGK1 deubiquitination during the glycolysis of pancreatic cancer
Source: Cell Death Dis. 2023 Feb 17;14(2):137. doi: 10.1038/s41419-023-05679-2 (PMC9938189; doi:10.1038/s41419-023-05679-2)
Supplement: Supplementary file 2 — Supplementray file [file 41419_2023_5679_MOESM2_ESM.docx]

| Primer | Sequence |
| --- | --- |
| PGK1Forward | TGGACGTTAAAGGGAAGCGG |
| PGK1Reverse | GCTCATAAGGACTACCGACTTGG |
| USP10Forward | ATTGAGTTTGGTGTCGATGAAGT |
| USP10Reverse | GGAGCCATAGCTTGCTTCTTTAG |
| KIF15Forward  KIF15Reverse  β-tublinForward  β-tublinReverse  GAPDH Forward  GAPDH Reverse | AAAACTGAGTTACGCAGCGTG  AGTTGCGAATACAGATTCCTGAG  TGGACTCTGTTCGCTCAGGT  TGCCTCCTTCCGTACCACAT  GGAGCGAGATCCCTCCAAAAT  GGCTGTTGTCATACTTCTCATGG |

Primers used in this study

Antibodies used in this study

| Protein | catalogue |
| --- | --- |
| PGK1  USP10  KIF15  Flag-tag  Myc-tag  Vimentin  MMP9  Twist  MMP12  LDHA  HK2  PKM2  GLUT1  beta-tublin  GAPDH  IgG  OTUD4  USP20 | 68035-1-Ig, Proteintech, Wuhan, China  67917-1-Ig, Proteintech, Wuhan, China  ab272615, Abcam, San francisco, USA  66008-4-Ig, Proteintech, Wuhan, China  60003-2-Ig, Proteintech, Wuhan, China  A19607, ABclonal, Wuhan, China  27306-1-AP, Proteintech, Wuhan, China  66544-1-Ig, Proteintech, Wuhan, China  A3713, ABclonal, Wuhan, China  66287-1-Ig, Proteintech, Wuhan, China  66974-1-Ig, Proteintech, Wuhan, China  A19102, ABclonal, Wuhan, China  66290-1-Ig, Proteintech, Wuhan, China  10094-1-AP, Proteintech, Wuhan, China  60004-1-Ig, Proteintech, Wuhan, China  30000-0-AP, Proteintech, Wuhan, China  25070-1-AP, Proteintech, Wuhan, China  17491-1-AP, Proteintech, Wuhan, China |
