# Supplementary figures and images for: KIF15 is essential for USP10-mediated PGK1 deubiquitination during the glycolysis of pancreatic cancer
Source: Cell Death Dis. 2023 Feb 17;14(2):137. doi: 10.1038/s41419-023-05679-2 (PMC9938189; doi:10.1038/s41419-023-05679-2)

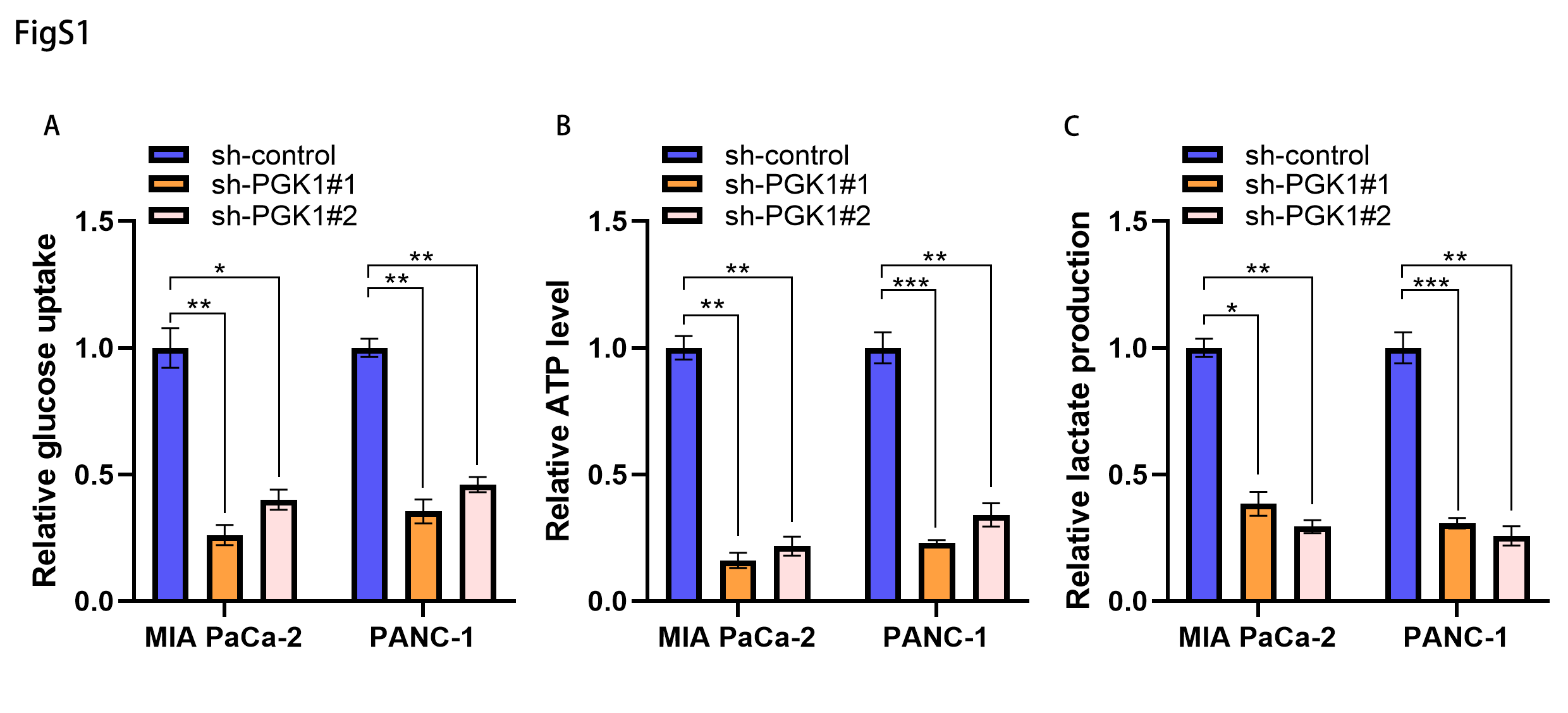

Supplement: Supplementary file 3 — SUPPLEMENTAL FigureS1 [file 41419_2023_5679_MOESM3_ESM.tif]

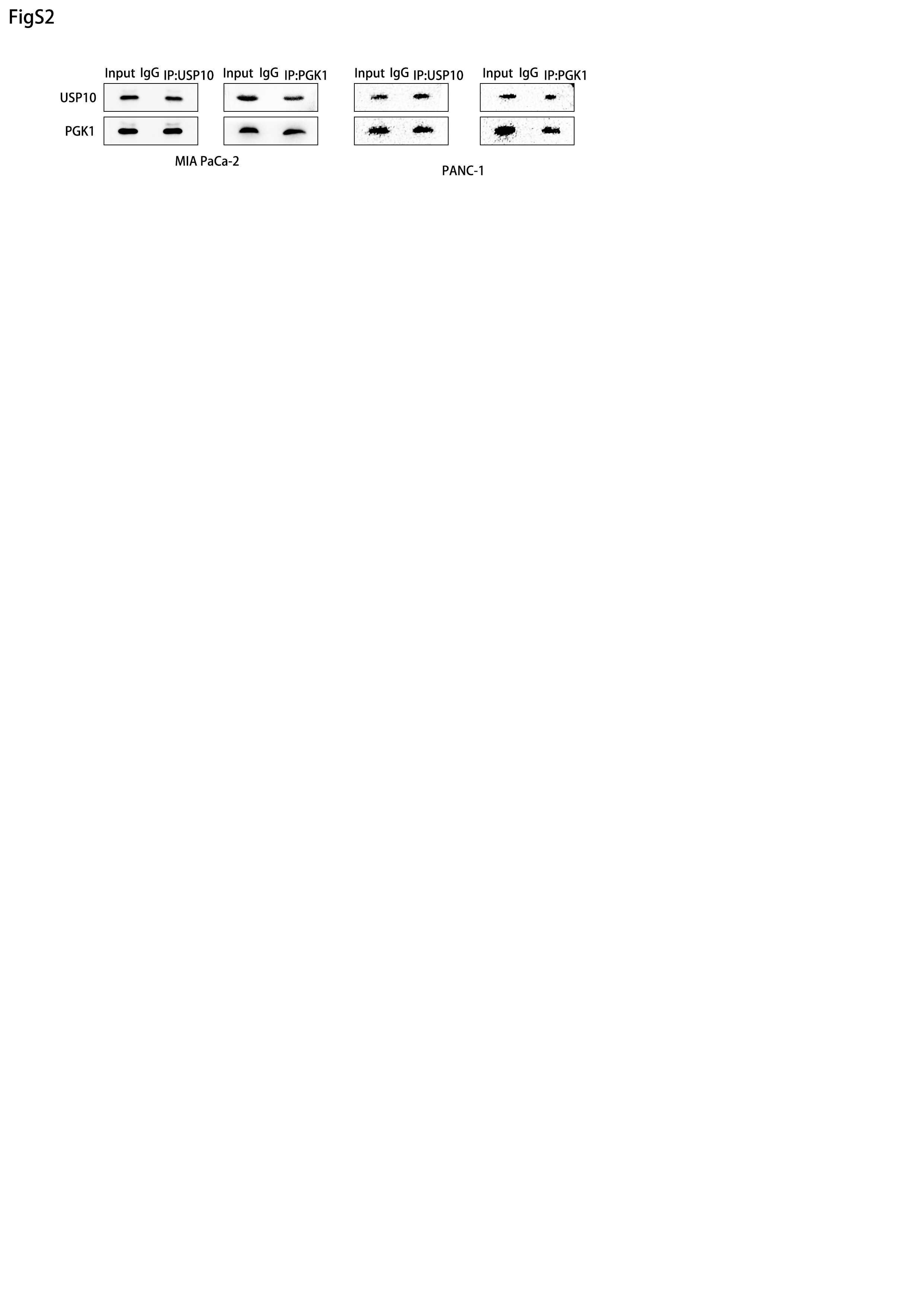

Supplement: Supplementary file 4 — SUPPLEMENTAL FigureS2 [file 41419_2023_5679_MOESM4_ESM.tif]

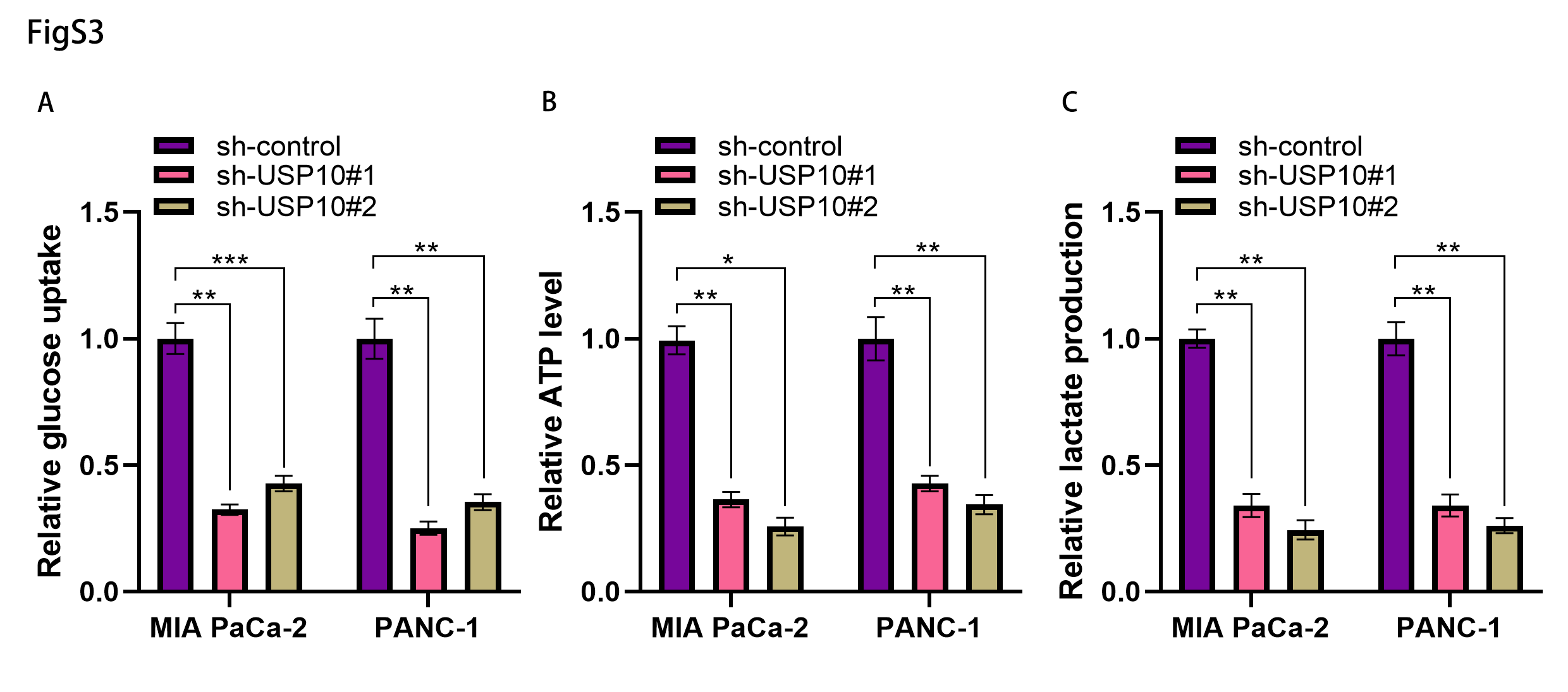

Supplement: Supplementary file 5 — SUPPLEMENTAL FigureS3 [file 41419_2023_5679_MOESM5_ESM.tif]

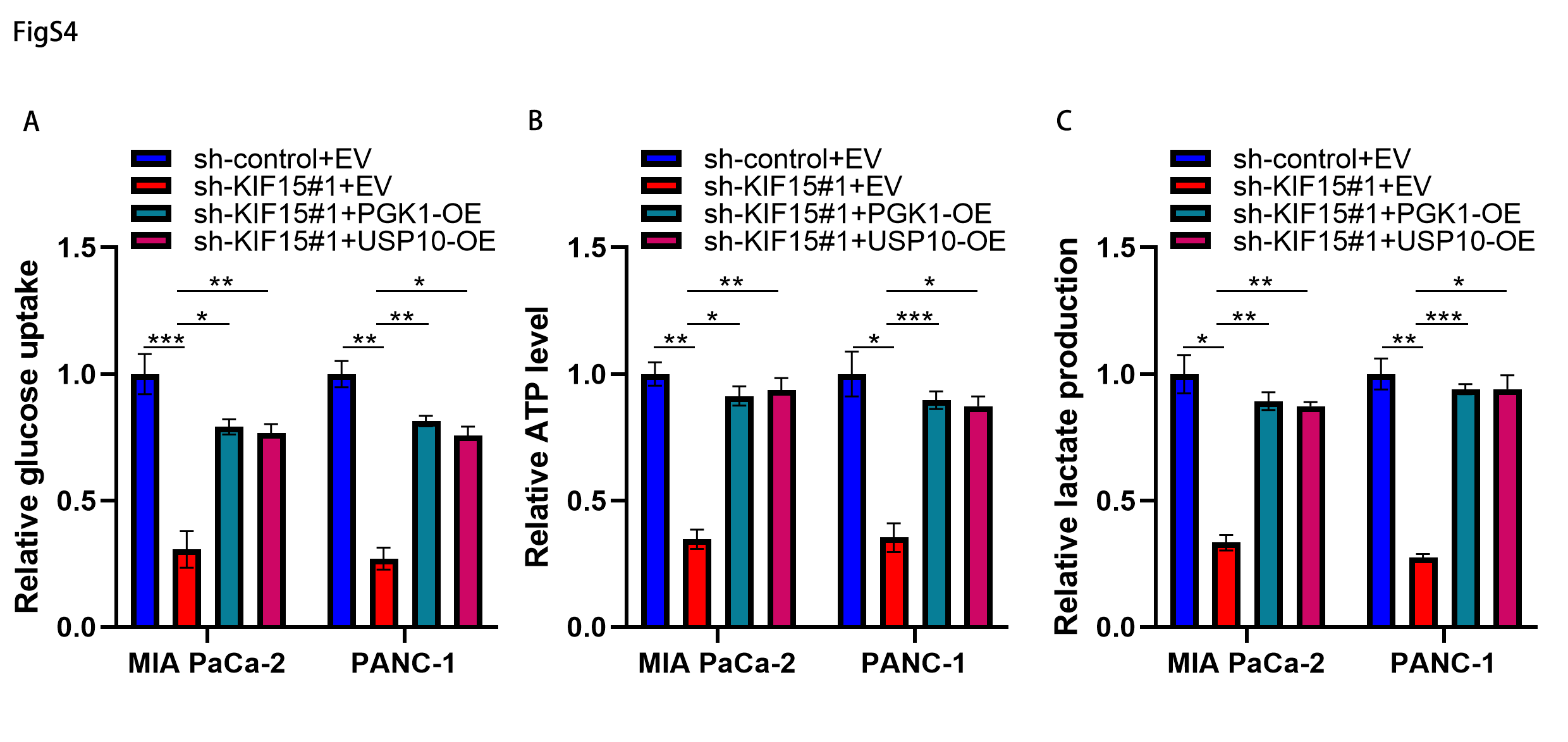

Supplement: Supplementary file 6 — SUPPLEMENTAL FigureS4 [file 41419_2023_5679_MOESM6_ESM.tif]
