# Supplementary material for: KIF15 is essential for USP10-mediated PGK1 deubiquitination during the glycolysis of pancreatic cancer
Source: Cell Death Dis. 2023 Feb 17;14(2):137. doi: 10.1038/s41419-023-05679-2 (PMC9938189; doi:10.1038/s41419-023-05679-2)
Supplement: Supplementary file 7 — Figure legend for supplementary figures [file 41419_2023_5679_MOESM7_ESM.docx]

**FigS1 PGK1 promoted the glycolytic capacity of PC cells**

A. The relative glucose uptake of PC cells transfected with PGK1 shRNAs. B. The relative ATP level of PC cells transfected with PGK1 shRNAs. C. The relative lactate production of PC cells transfected with PGK1 shRNAs.

**FigS2 USP10 interacted with PGK1 protein in PC cells**

A. The interaction of USP10 and PGK1 in PANC-1 or MIA PaCa-2 cells was verified by Co-IP assay and visualized by western-blot.

**FigS3 USP10 promoted the glycolytic capacity of PC cells**

A. The relative glucose uptake of PC cells transfected with USP10 shRNAs. B. The relative ATP level of PC cells transfected with USP10 shRNAs. C. The relative lactate production of PC cells transfected with USP10 shRNAs.

**FigS4 KIF15 influenced the aerobic glycolysis through PGK1 and USP10**

A. The relative glucose uptake of PC cells co-transfected with KIF15 shRNA and control, PGK1 overexpression or USP10 overexpression plasmids. B. The relative ATP level of PC cells transfected with indicated plasmids or shRNA. C. The relative lactate production of PC cells transfected with indicated plasmids or shRNA.
