# Supplementary material for: KIF15 is essential for USP10-mediated PGK1 deubiquitination during the glycolysis of pancreatic cancer
Source: Cell Death Dis. 2023 Feb 17;14(2):137. doi: 10.1038/s41419-023-05679-2 (PMC9938189; doi:10.1038/s41419-023-05679-2)

fig1C UP

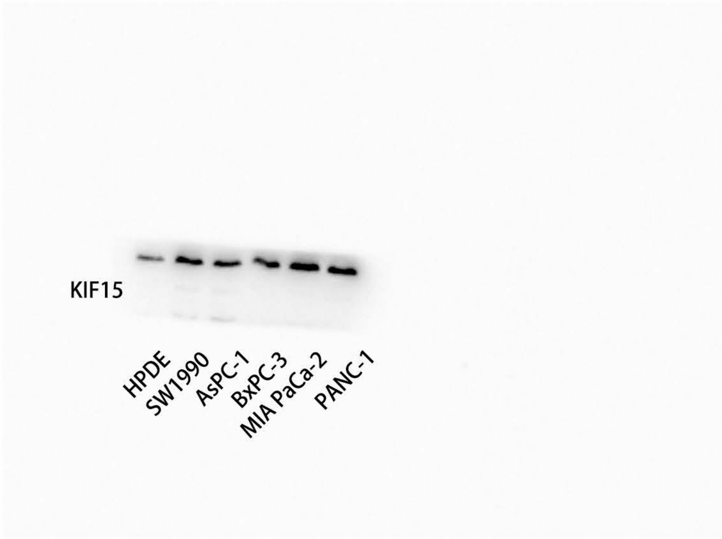

fig1C down

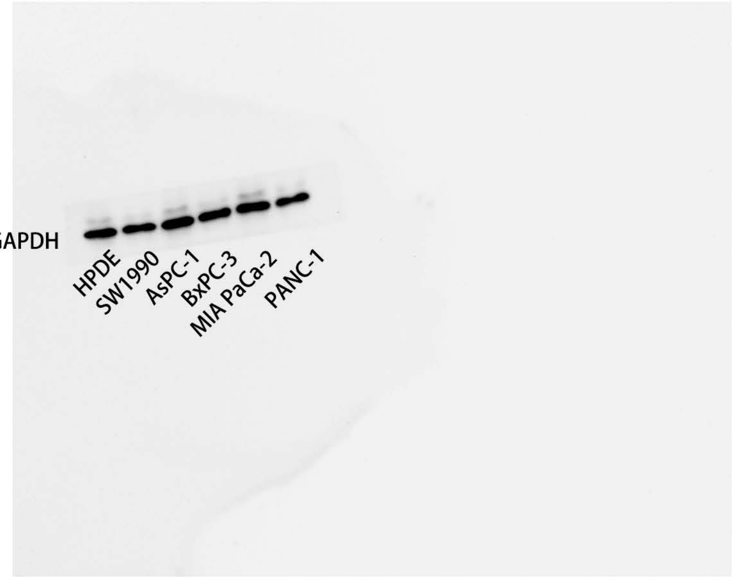

fig1E UP

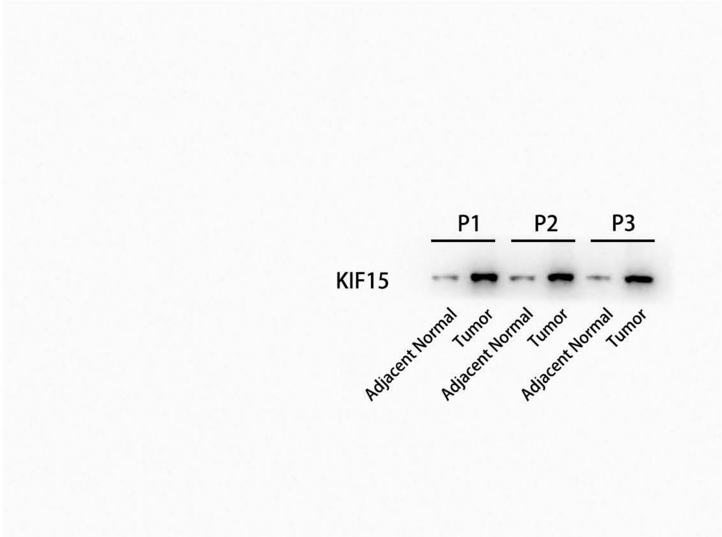

fig1E down

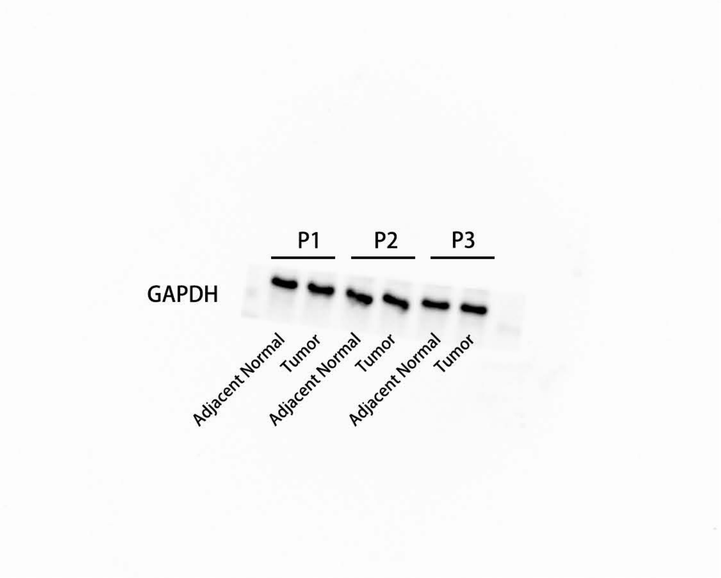

fig2H up first

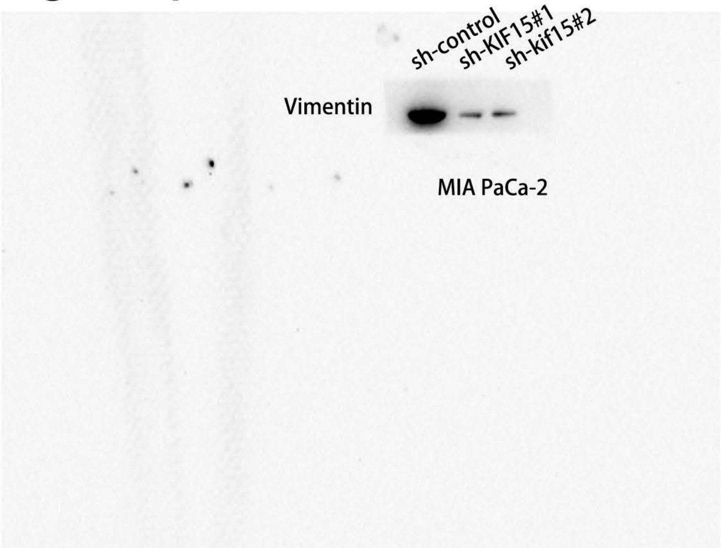

fig2H up second

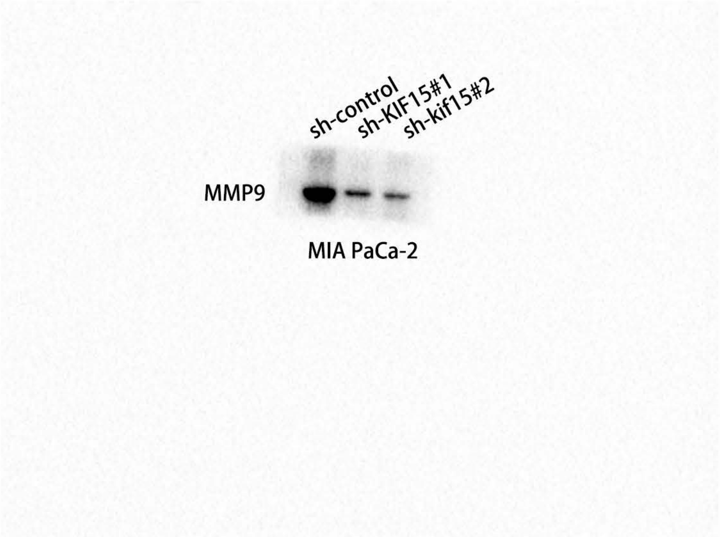

fig2H up third

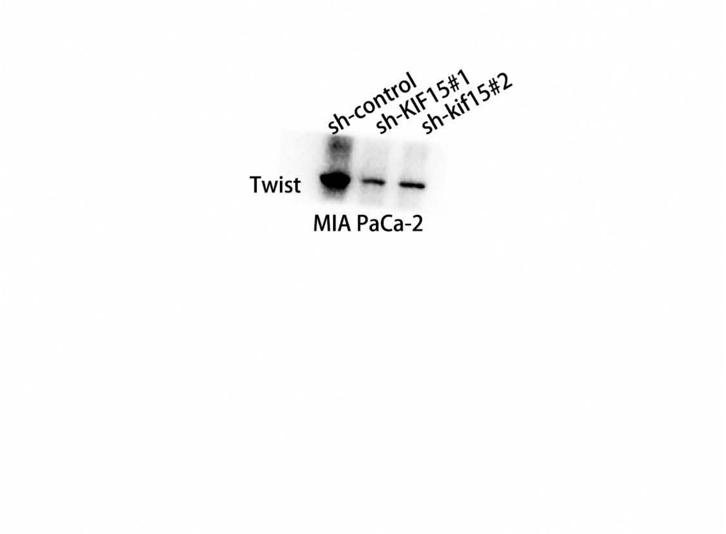

fig2H up fourth

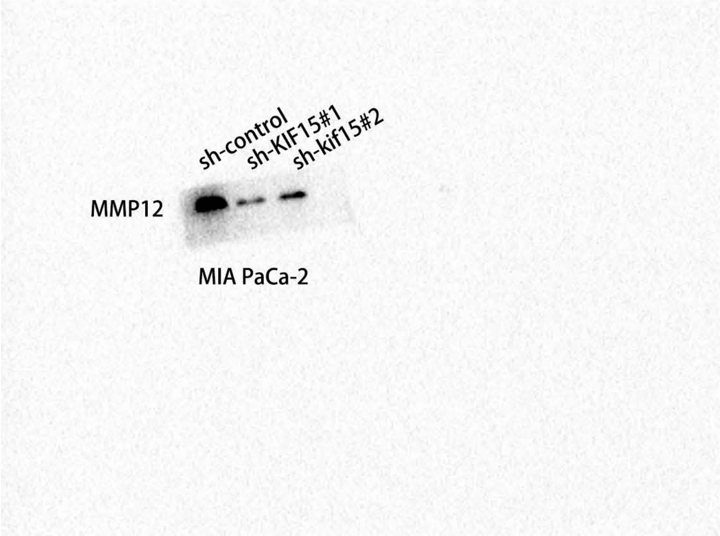

FIG2H up fifth

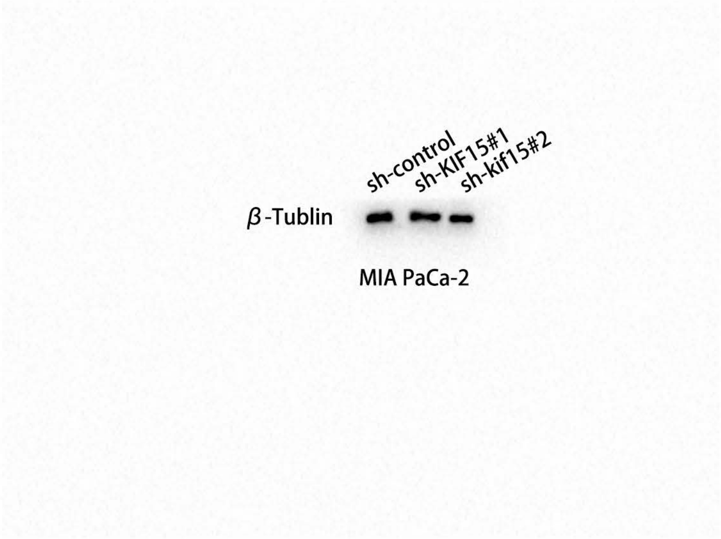

FIG2H down first

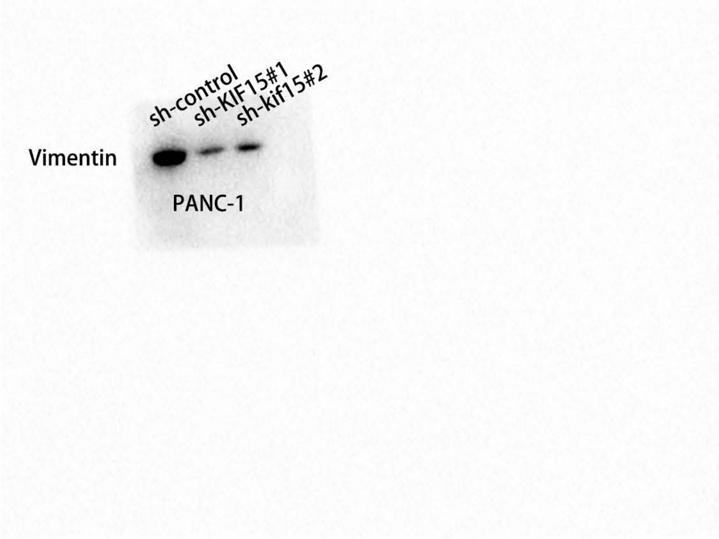

FIG2H down second

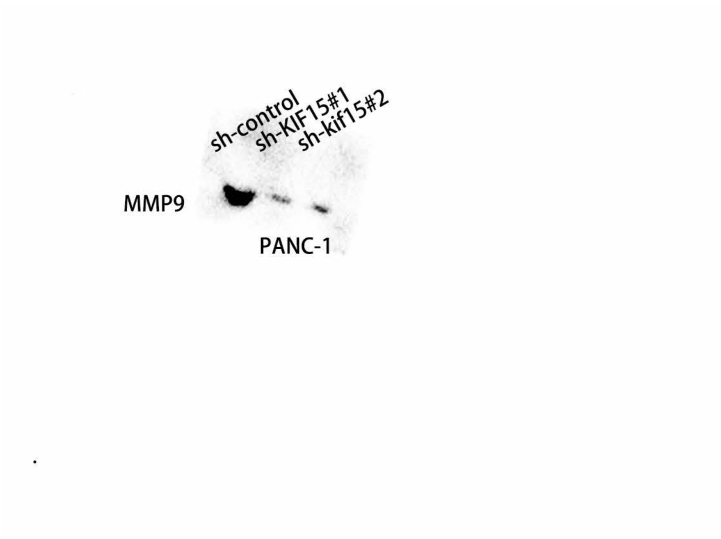

FIG2H down tird

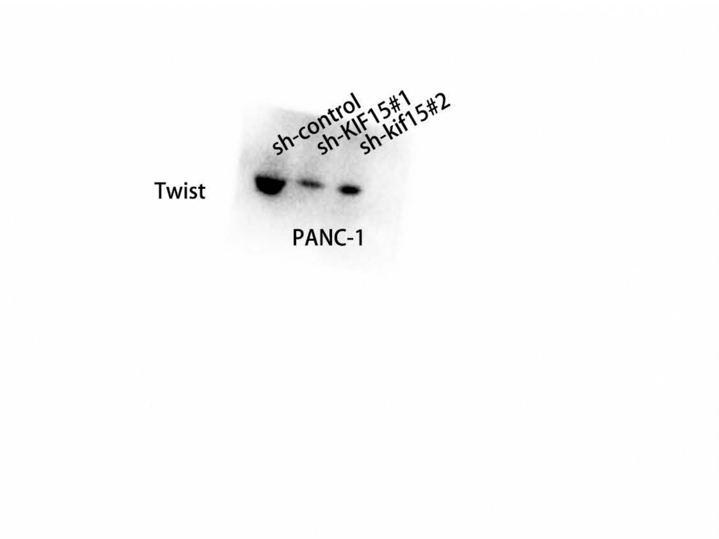

FIG2H down fourth

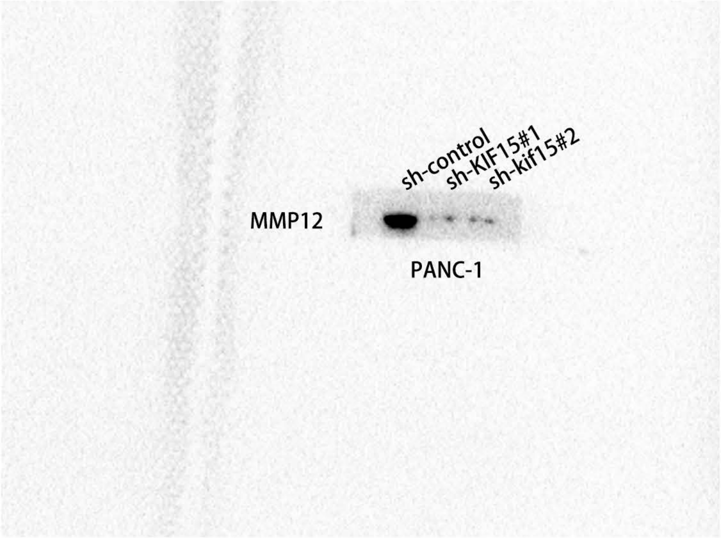

FIG2H down fifth

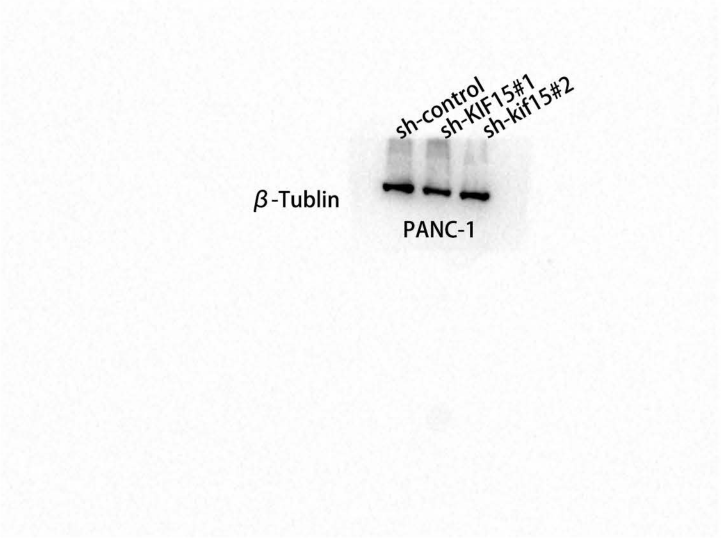

FIG3A left first

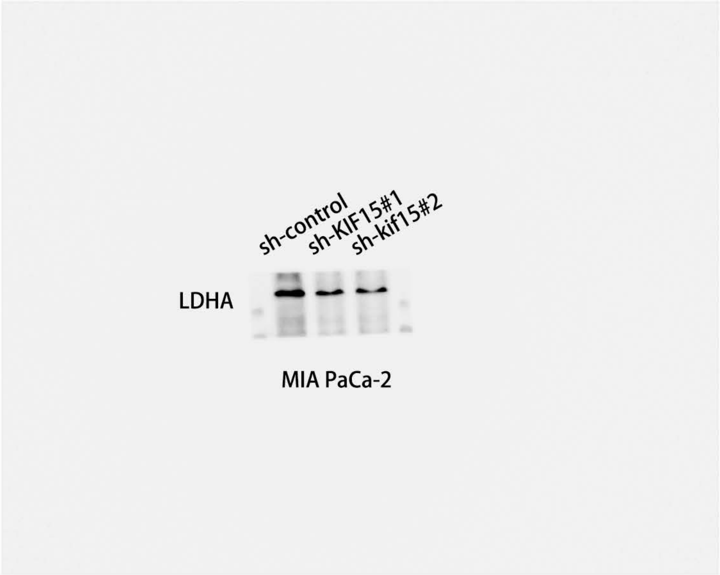

FIG3A left second

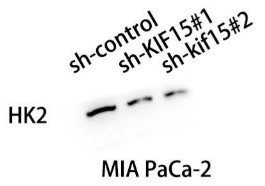

FIG3A left third

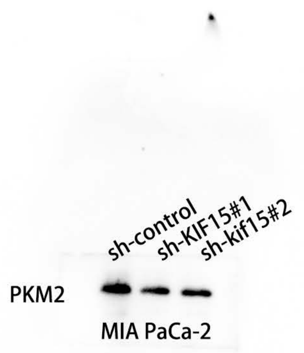

FIG3A left fourth

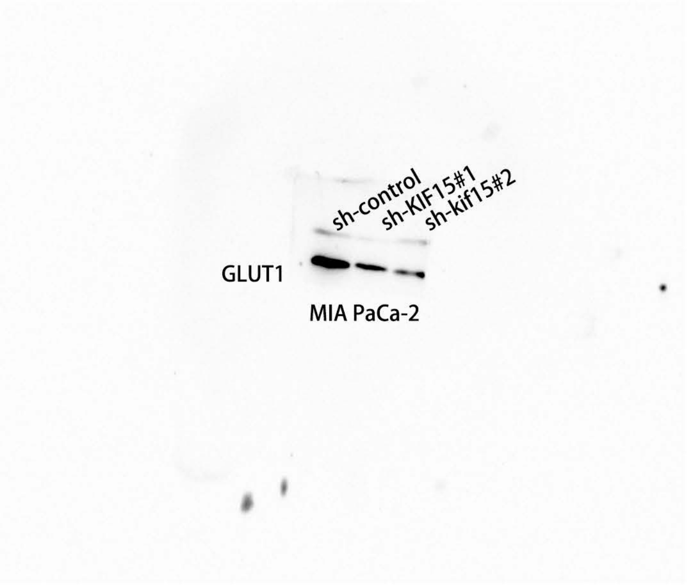

FIG3A left fifth

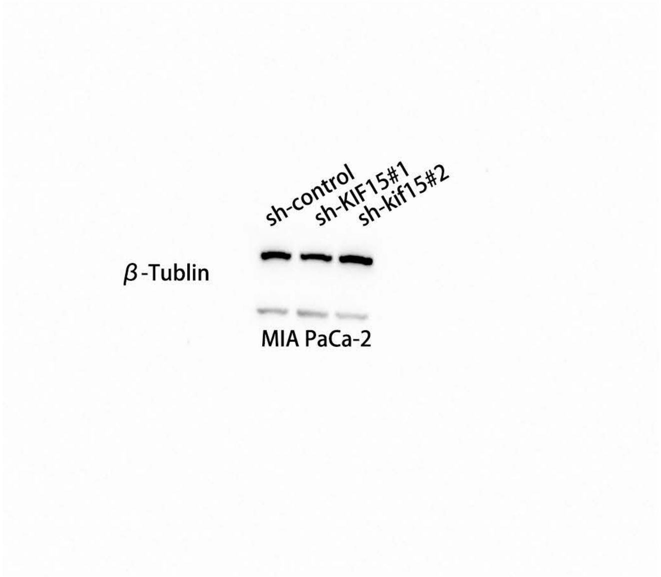

FIG3aA right first

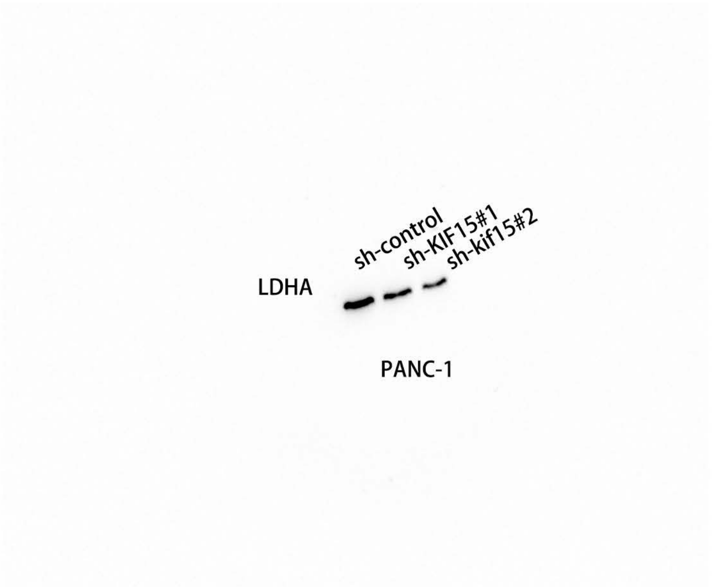

FIG3A right second

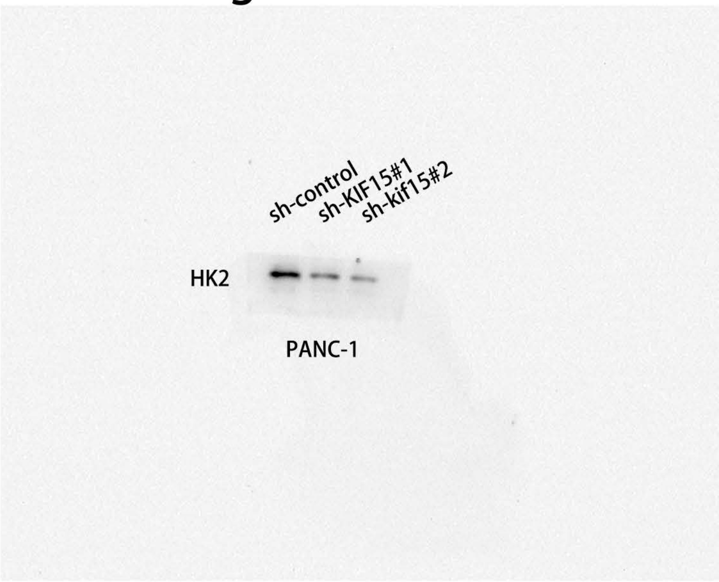

FIG3A right third

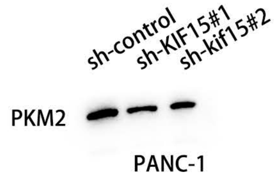

FIG3A right fourth

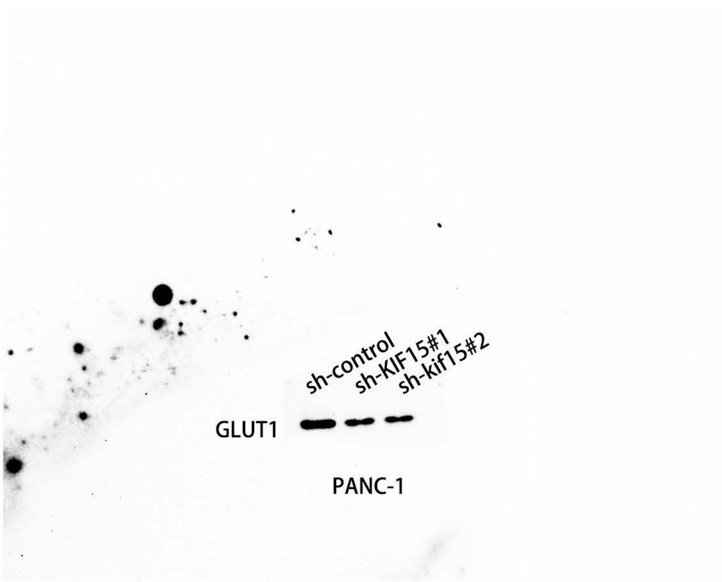

FIG3A right fifth

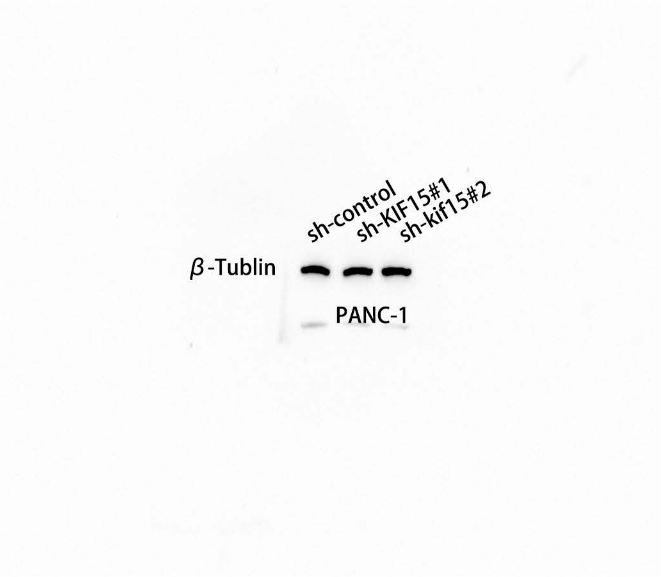

FIG4C up first

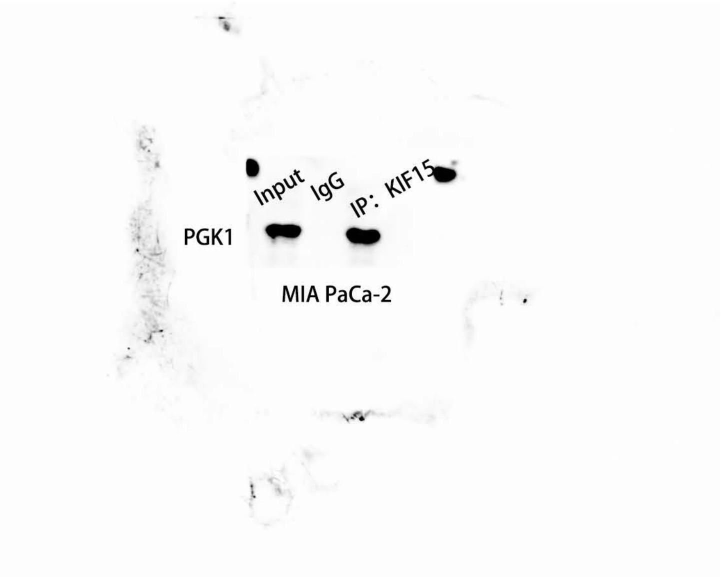

FIG4C down first

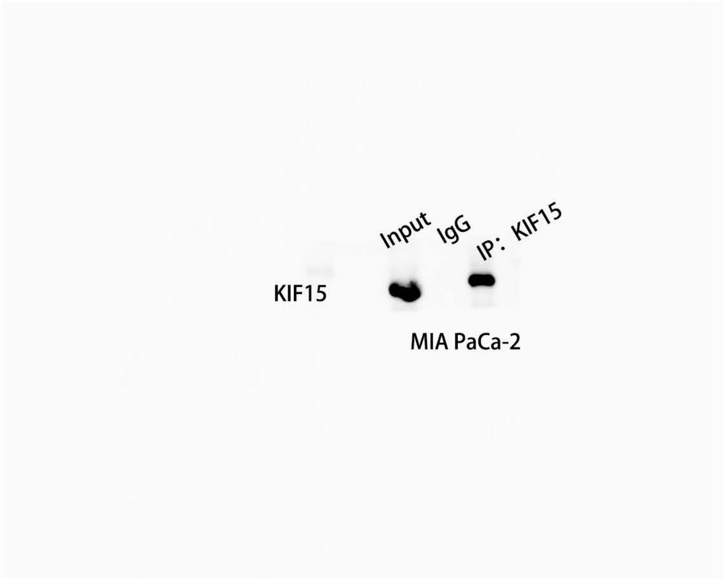

FIG4C up second

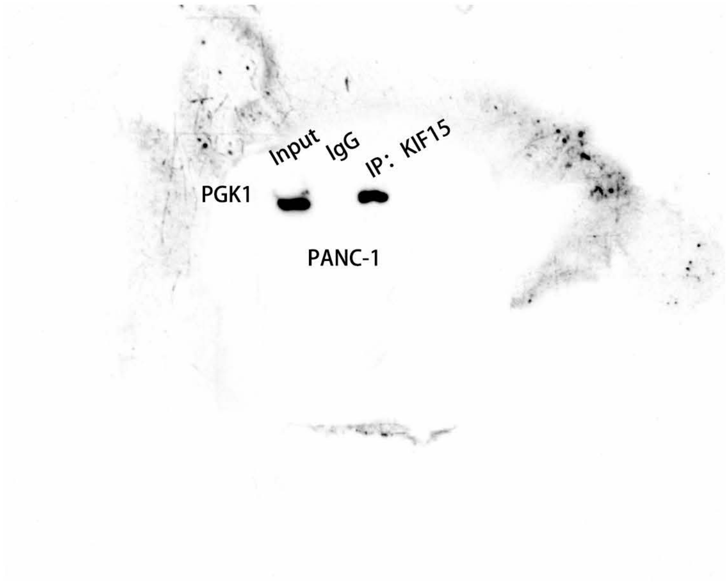

FIG4C down second

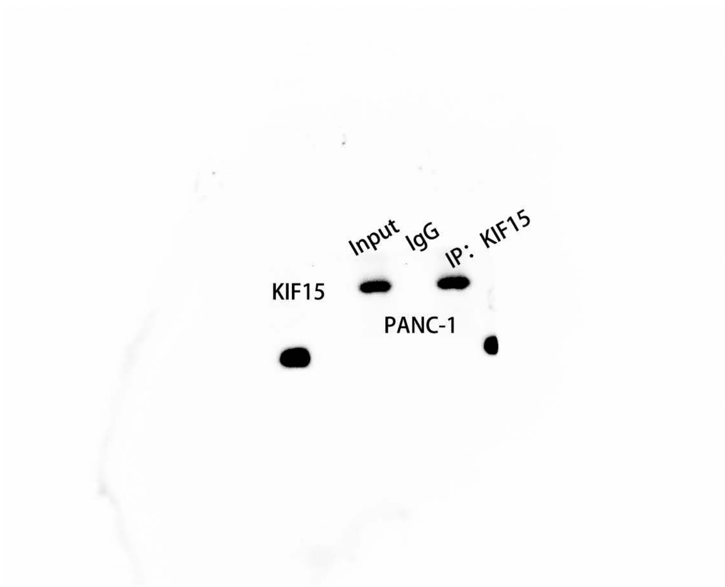

FIG4C up third

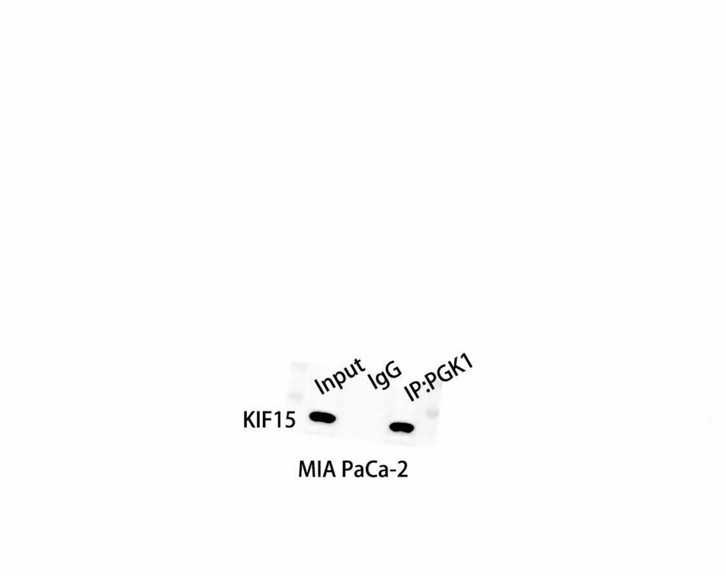

FIG4C down third

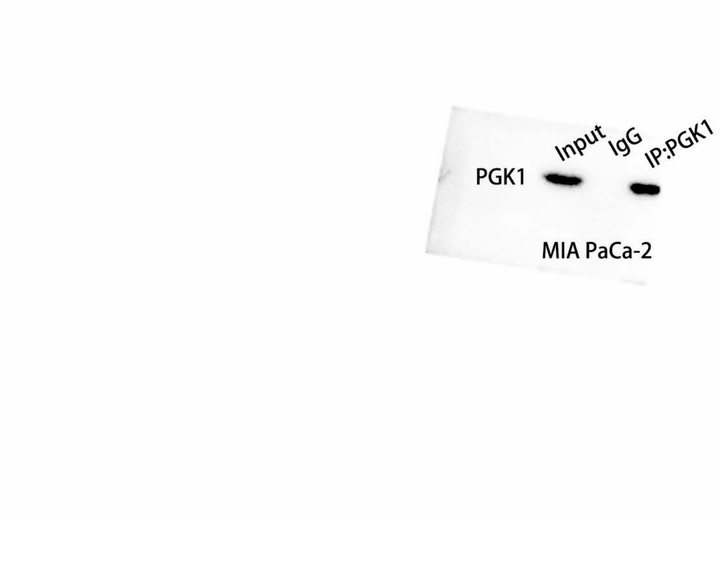

FIG4C up fourth

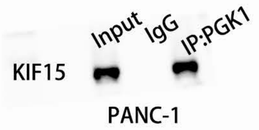

FIG4C down fourth

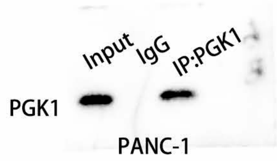

FIG5B left first

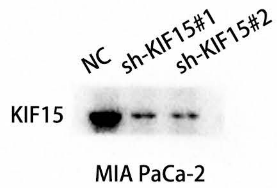

FIG5B left second

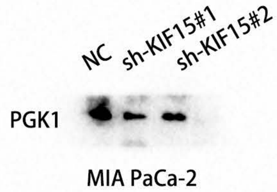

FIG5B left third

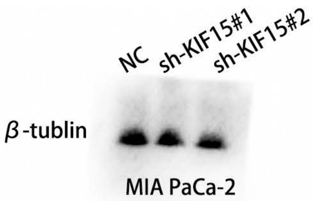

FIG5B right first

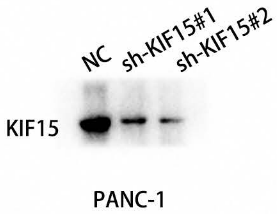

FIG5B right second

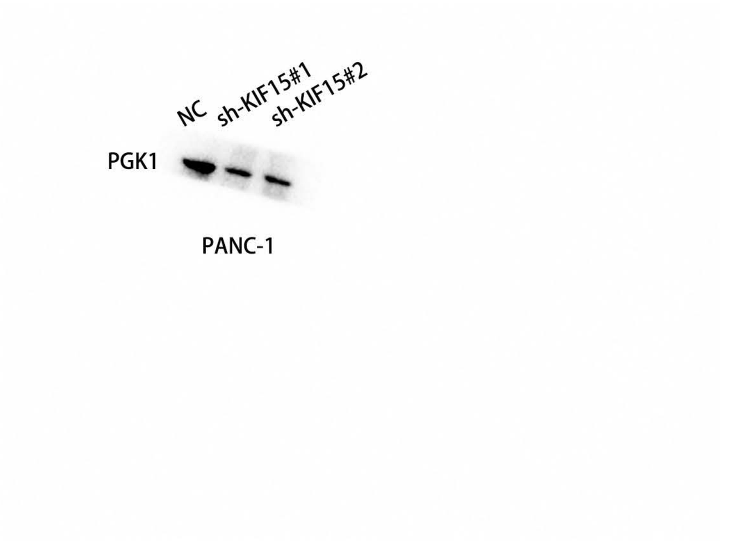

FIG5B right third

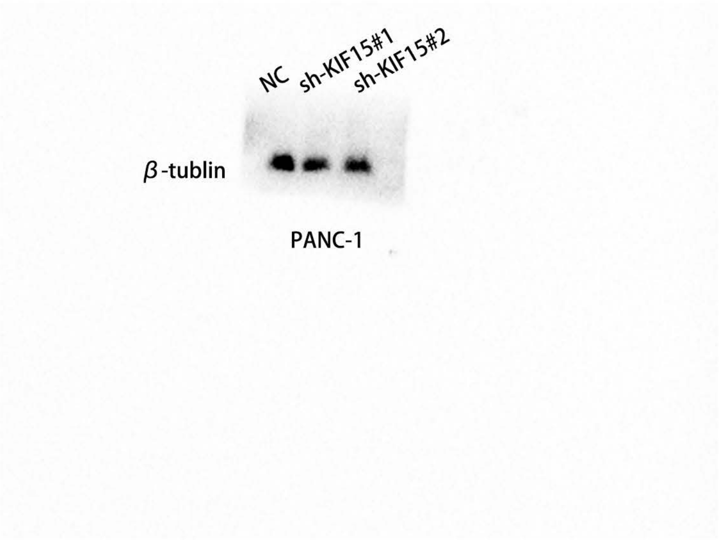

FIG5C left first

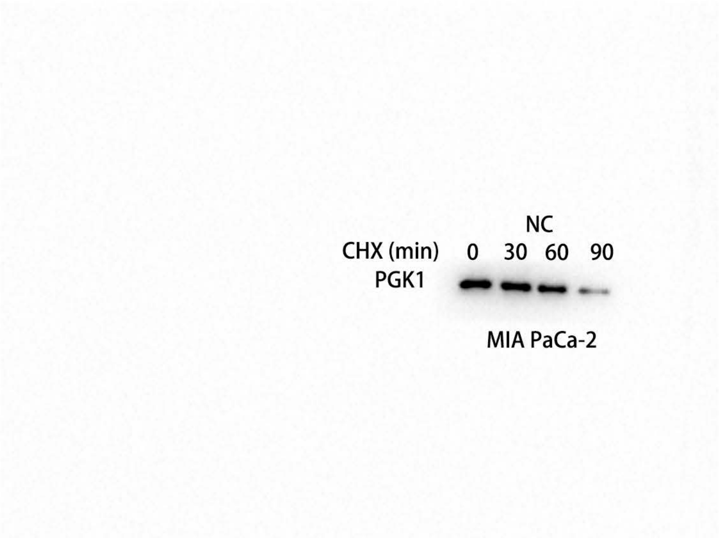

FIG5C left second

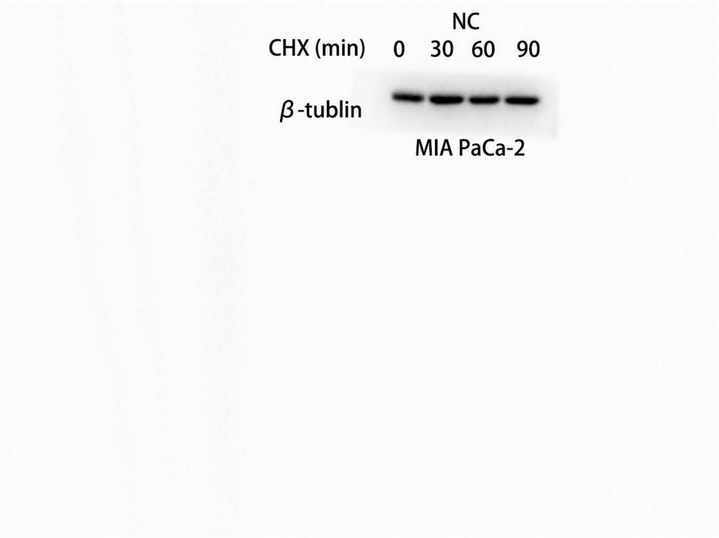

FIG5C left third

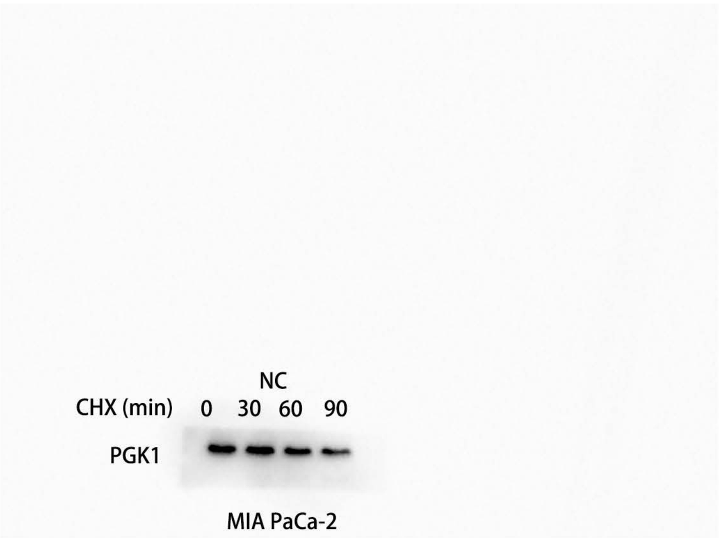

FIG5C left fourth

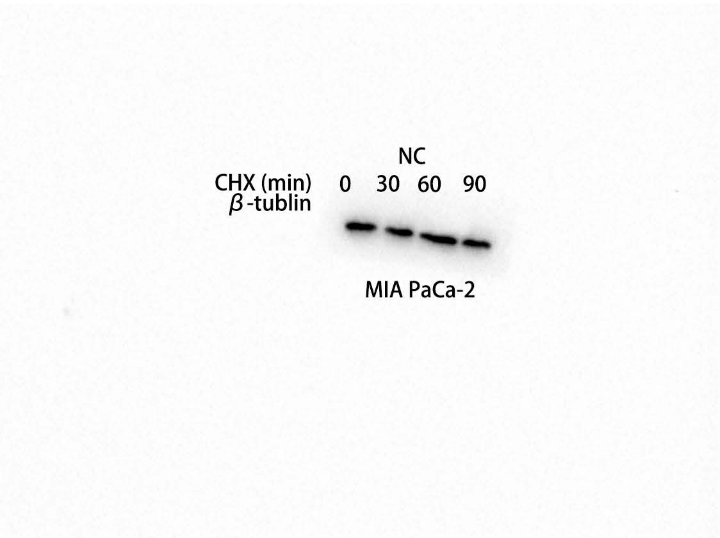

FIG5C right first

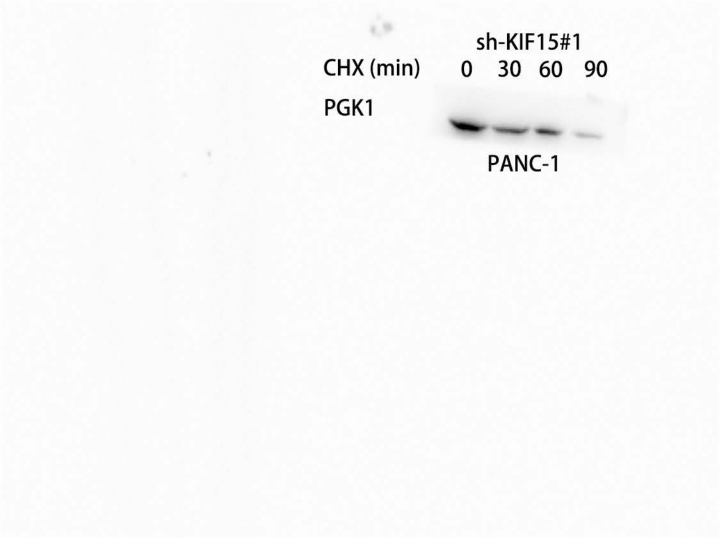

FIG5C right second

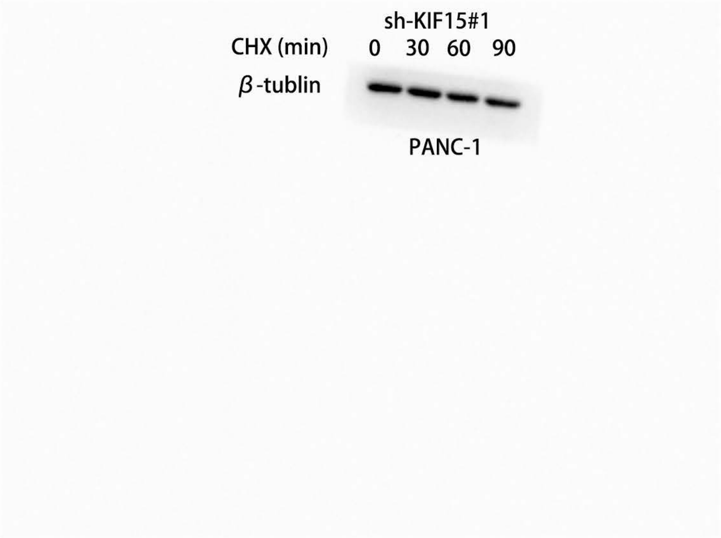

FIG5C right third

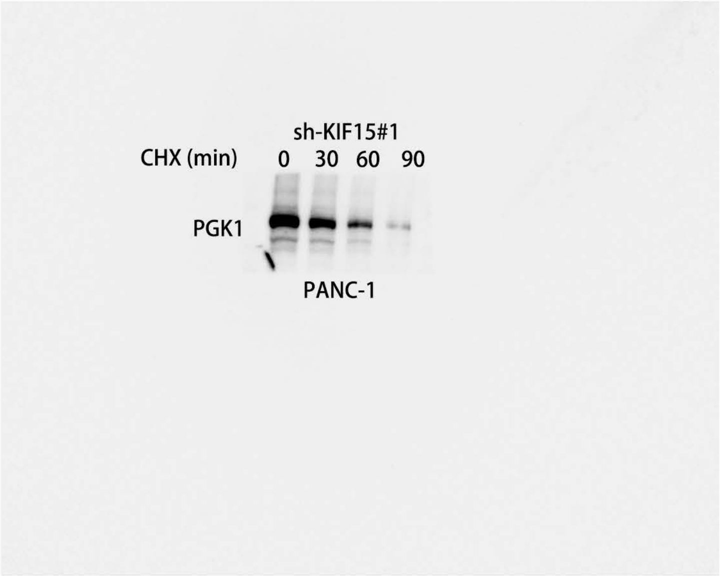

FIG5C right fourth

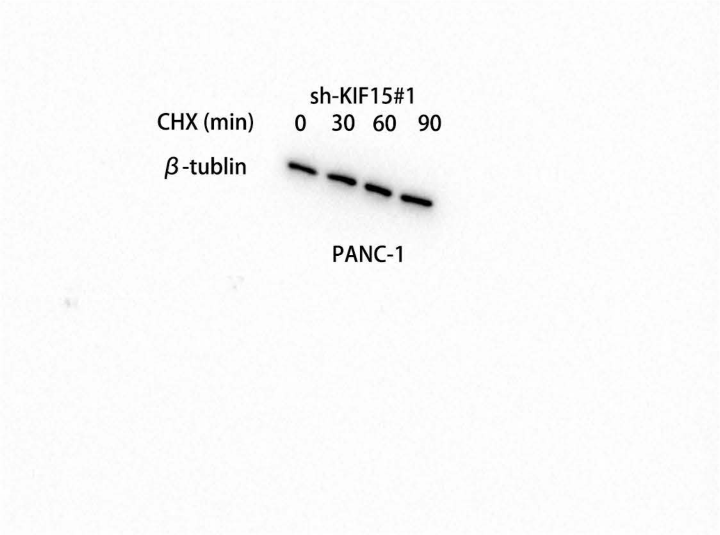

FIG5F left up

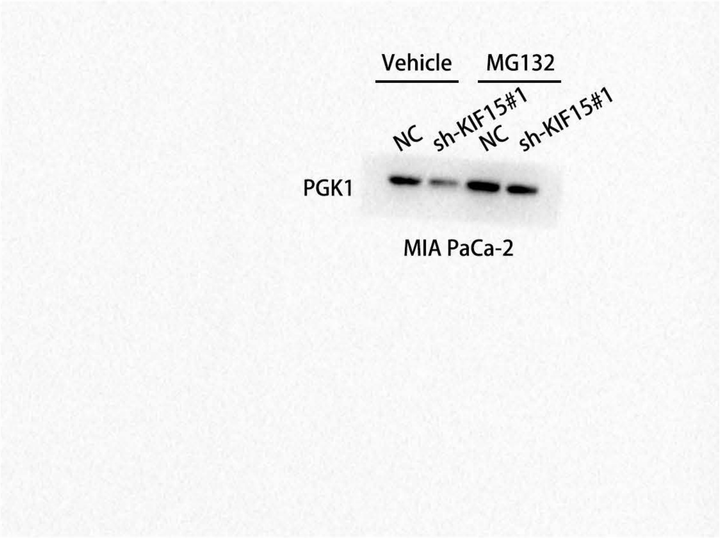

FIG5F left down

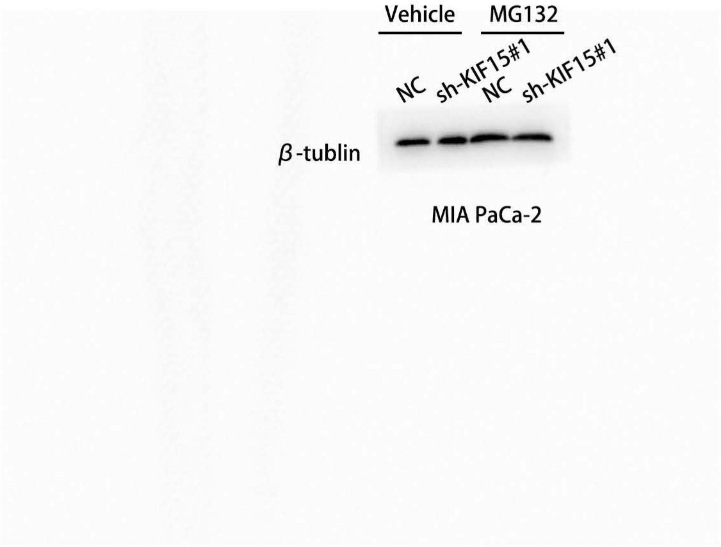

FIG5F right up

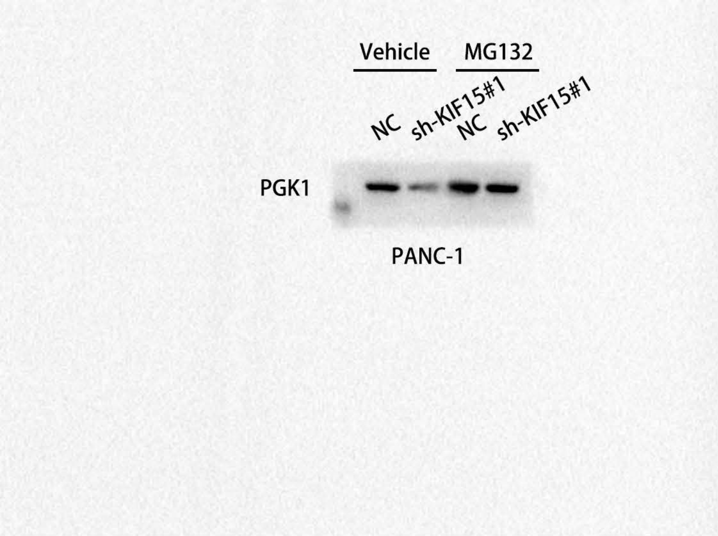

FIG5F right down

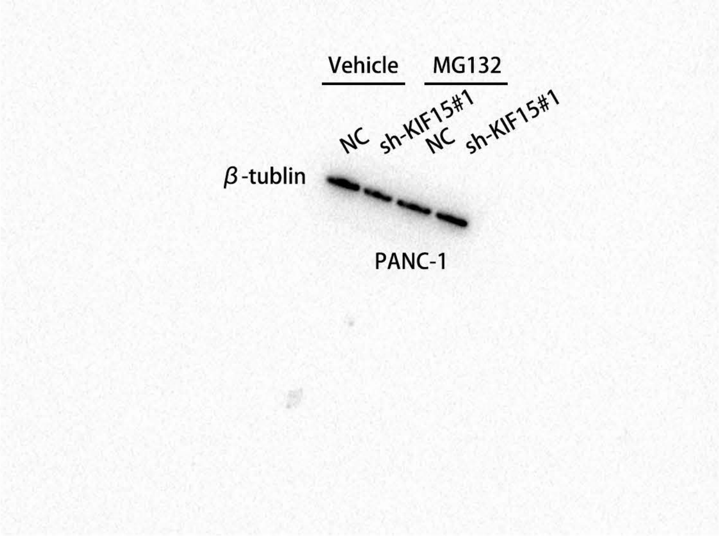

FIG5G first

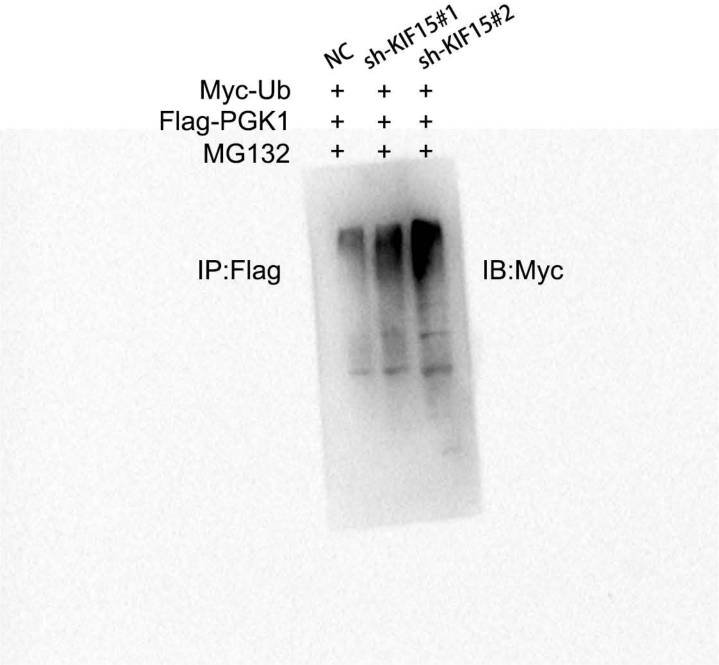

FIG5G second

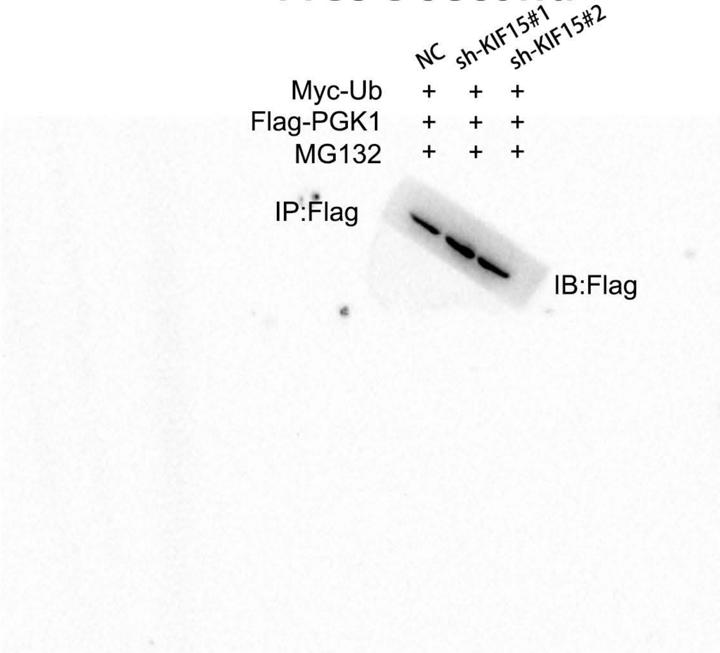

FIG5G third

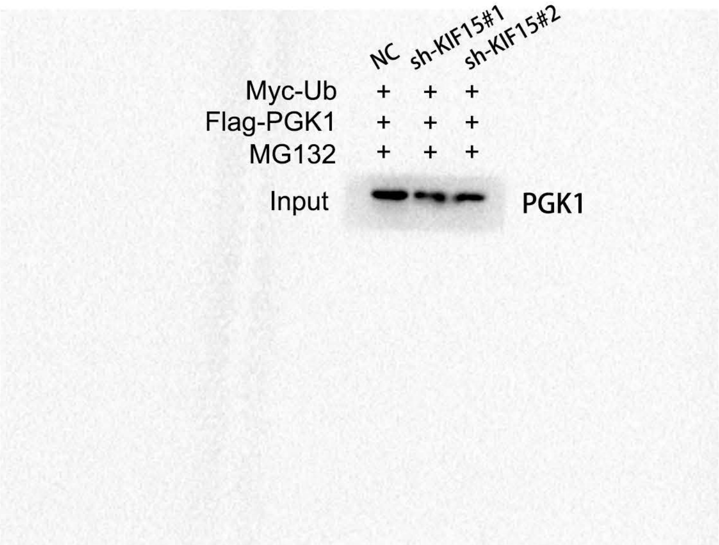

FIG5G fourth

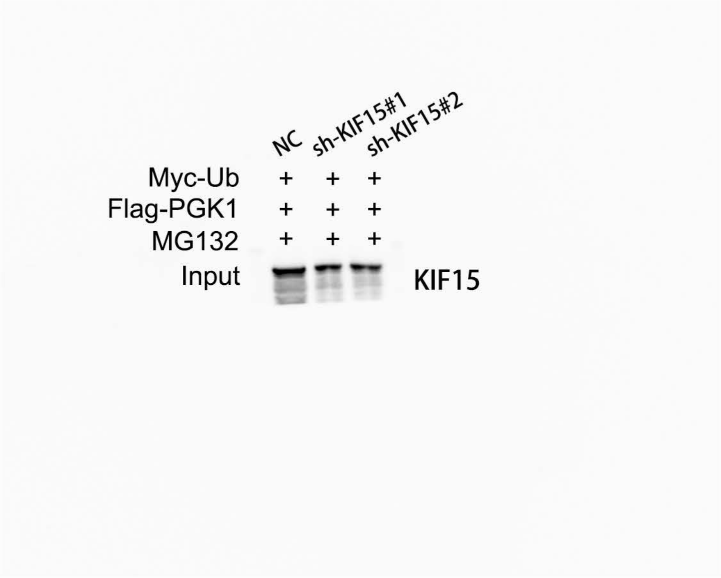

FIG5G fifth

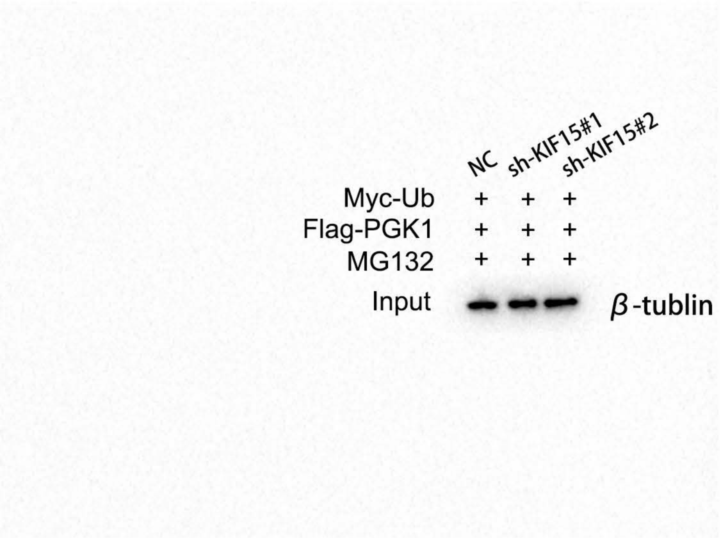

FIG5H left first

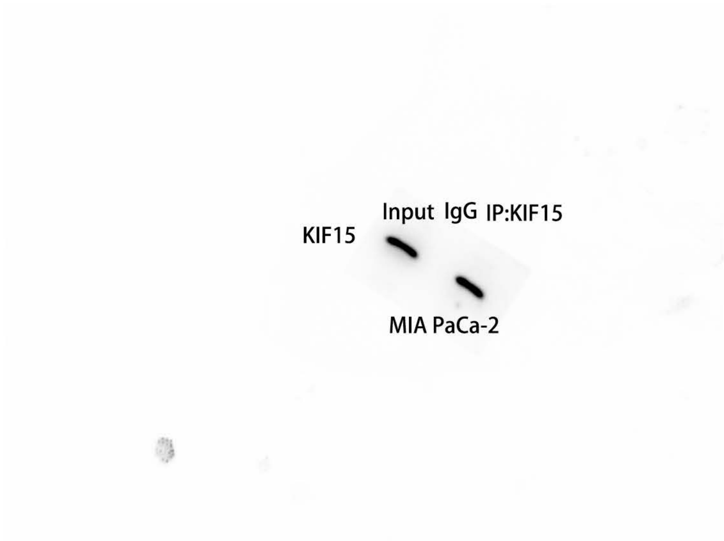

FIG5H left second

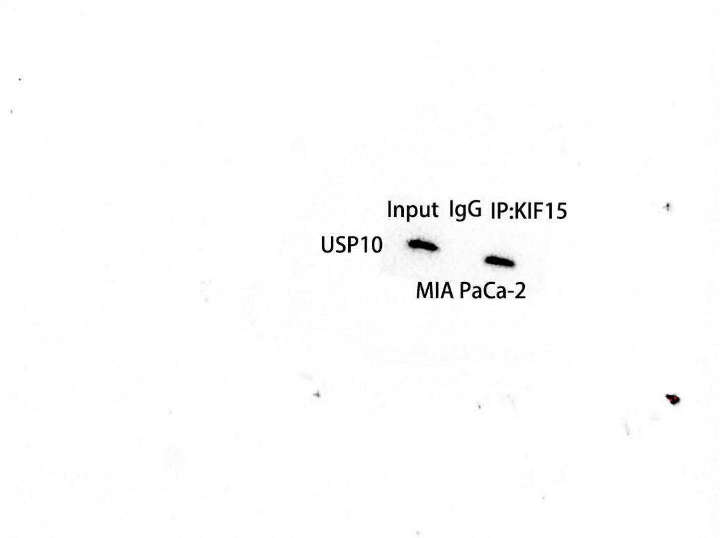

FIG5H right first

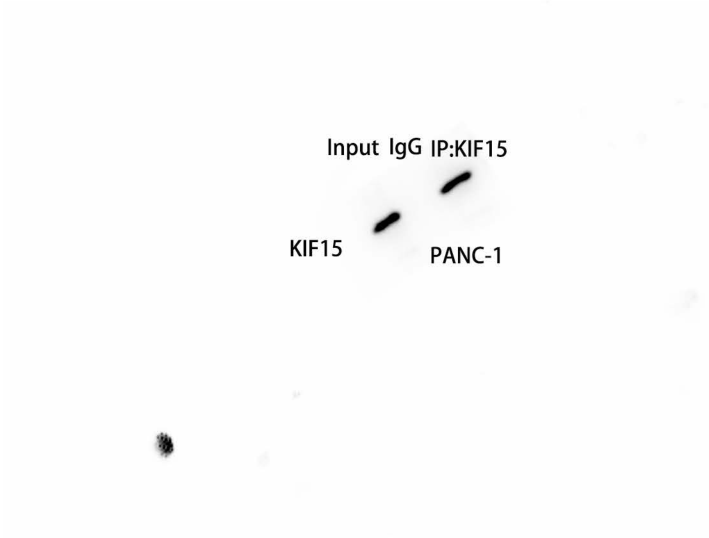

FIG5H right second

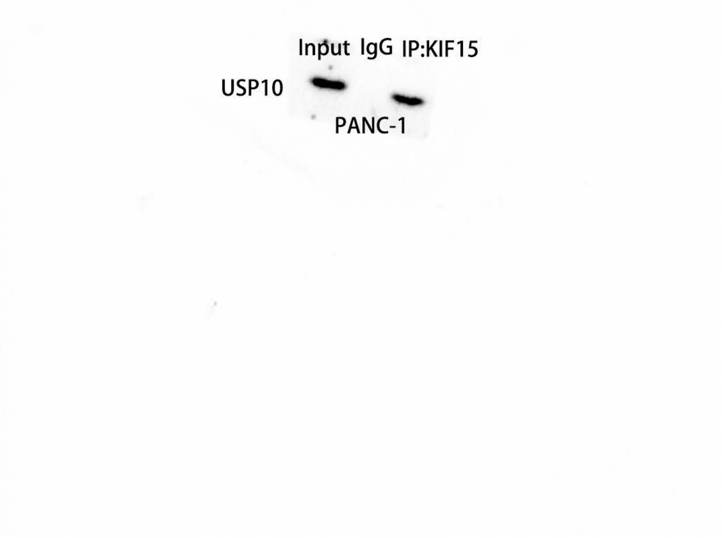

FIG5H left third

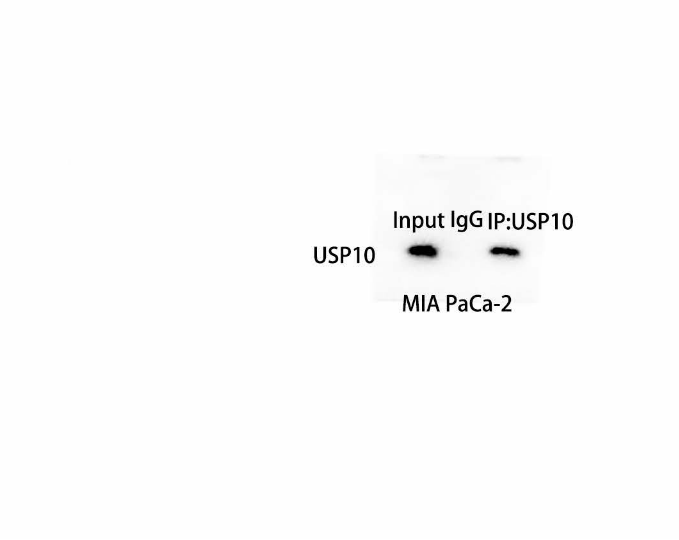

FIG5g lef fourth

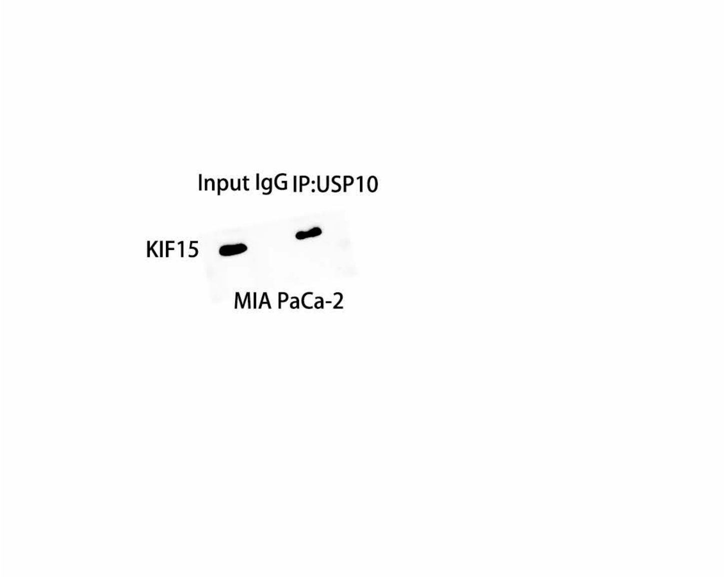

FIG5H right third

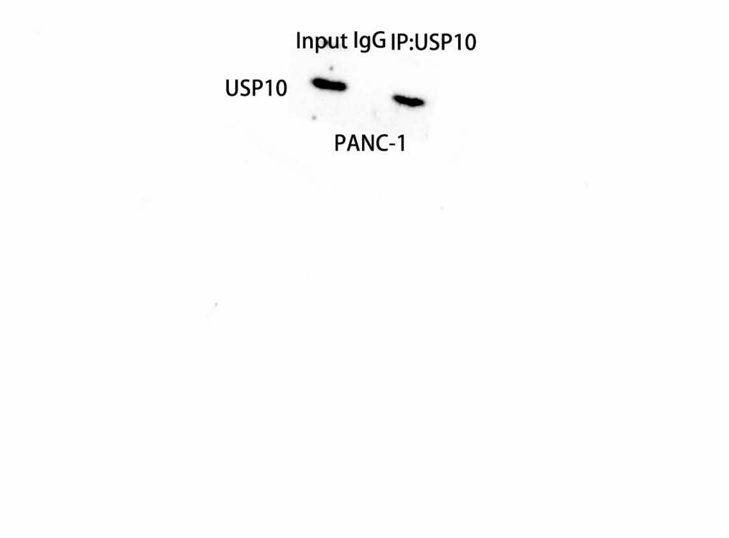

Fig5H right fourth

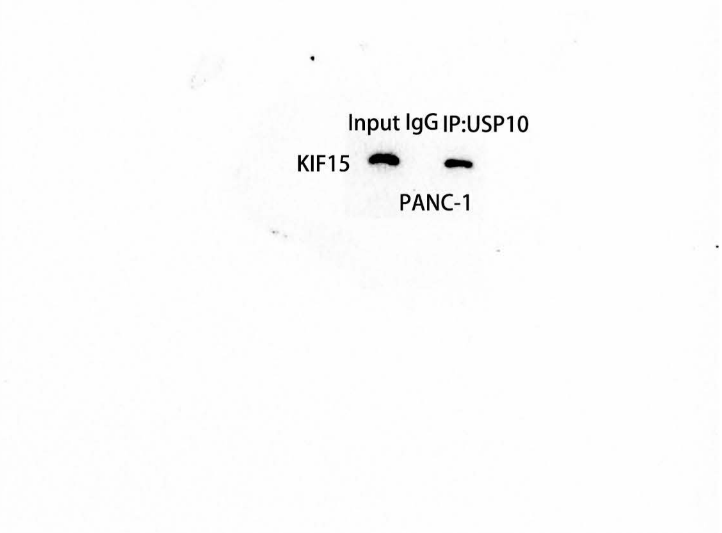

Fig5H left fifth

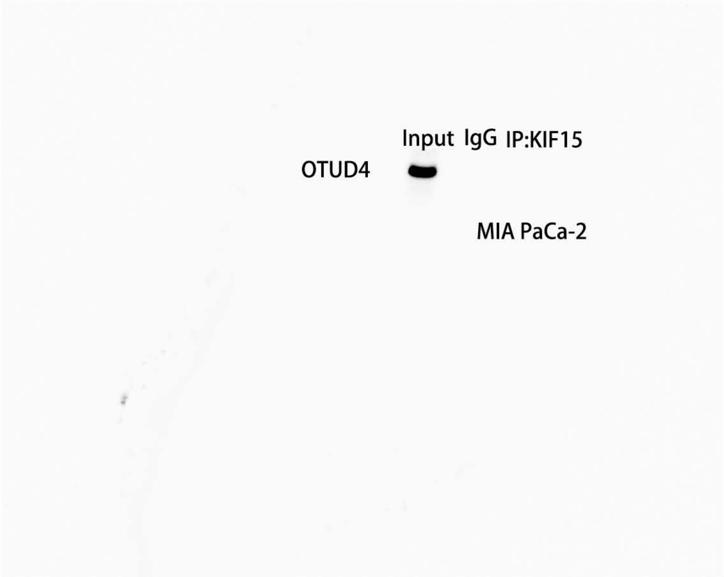

Fig5H left sixth

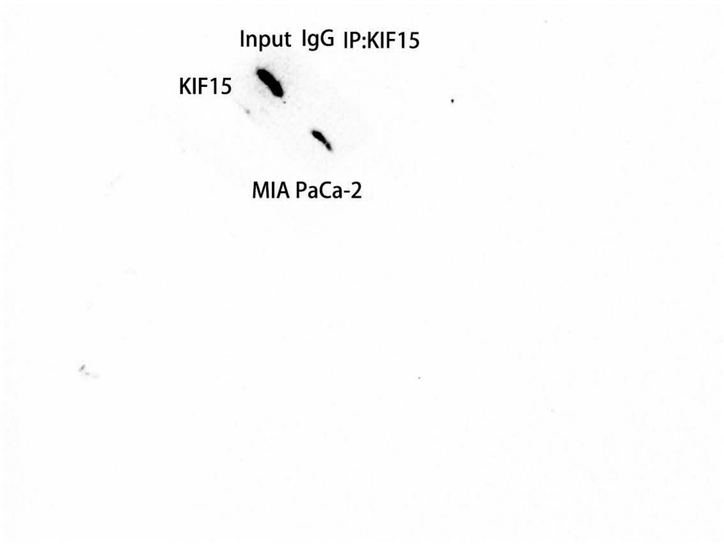

Fig5H right fifth

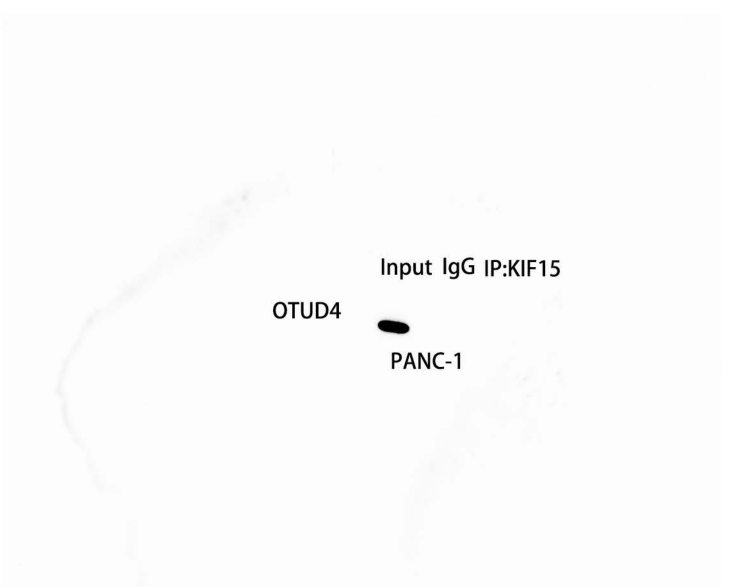

Fig5H right sixth

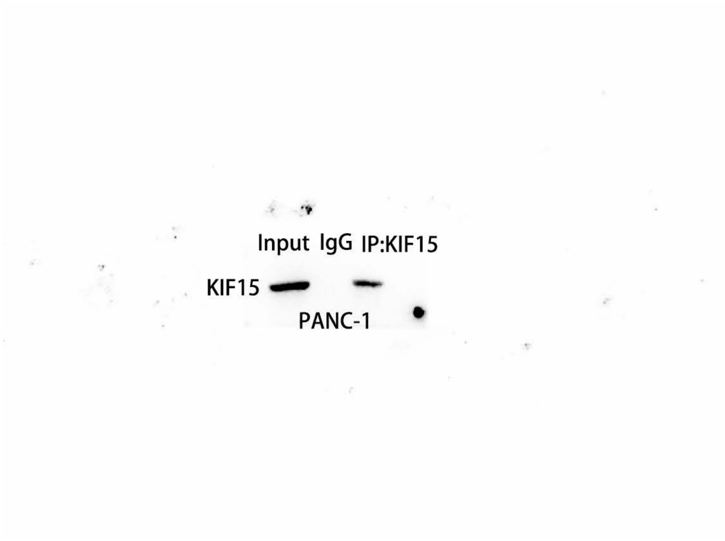

Fig5H left seventh

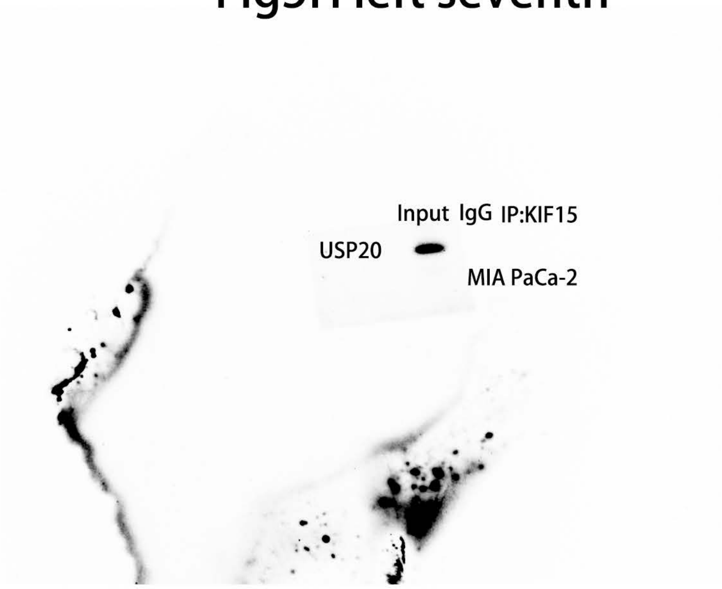

Fig5H left eighth

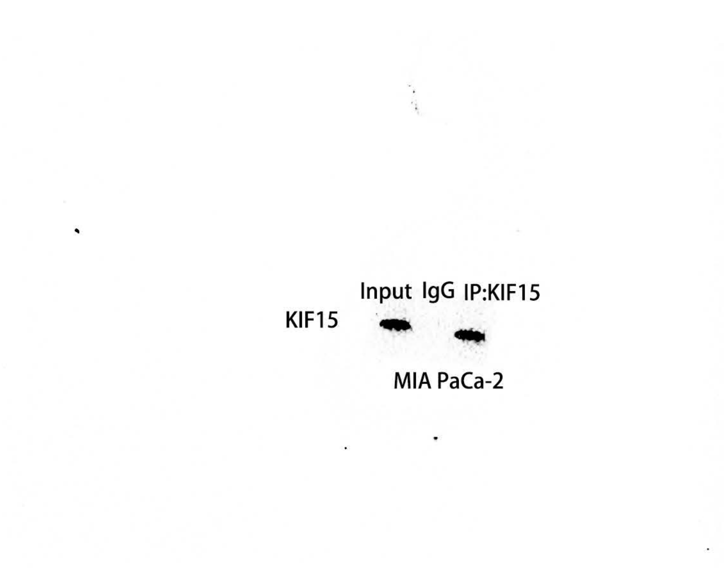

Fig5H right seventh

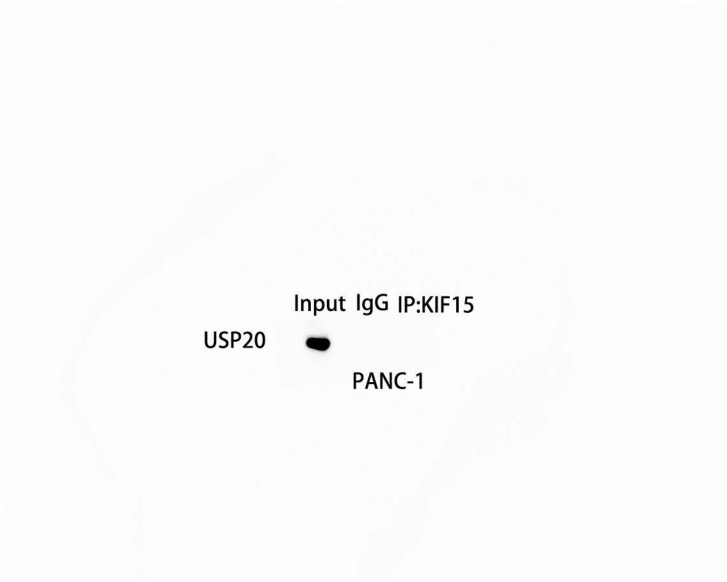

Fig5H right eighth

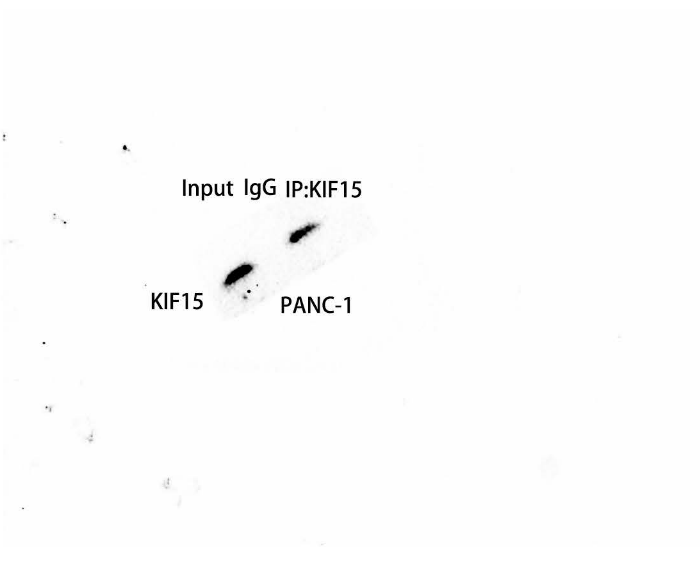

FIG5M up

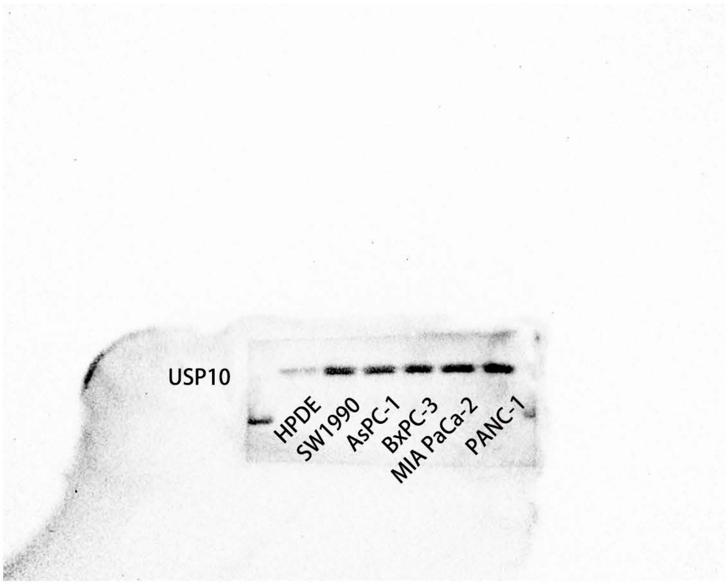

FIG5M down

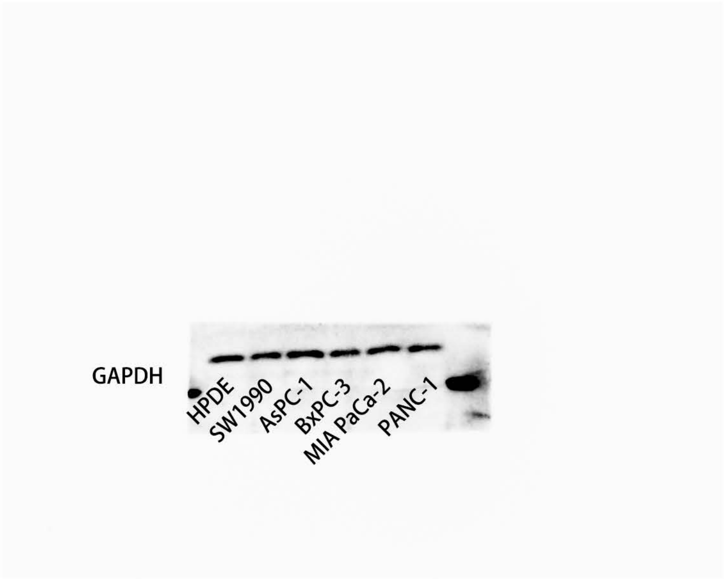

FIG6B left first

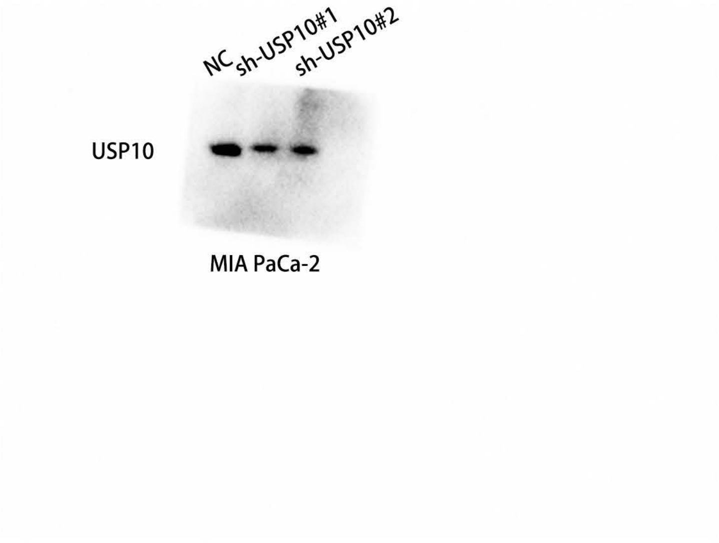

FIG6B left second

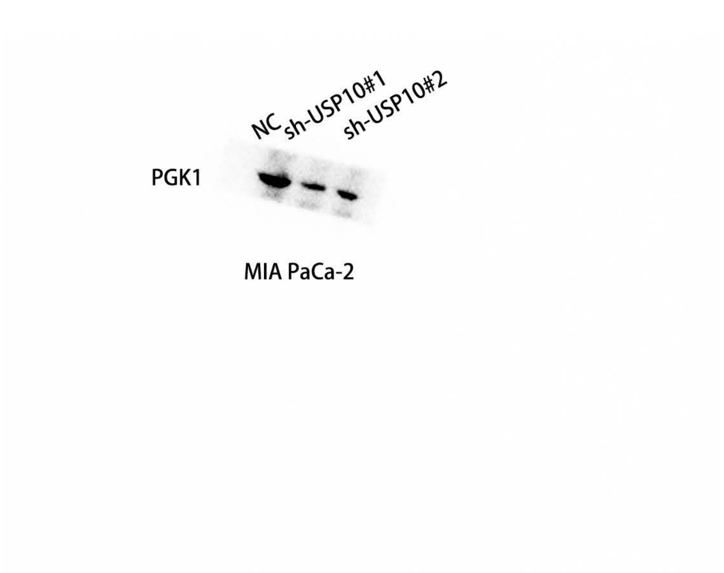

FIG6B left third

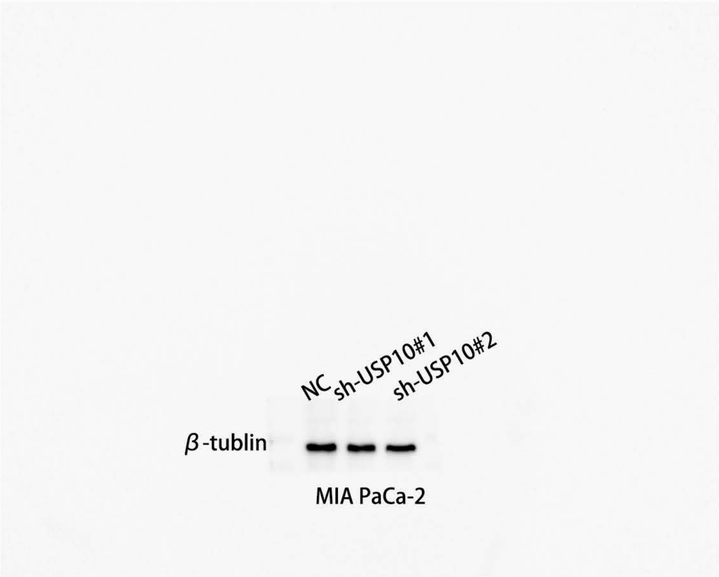

FIG6B right first

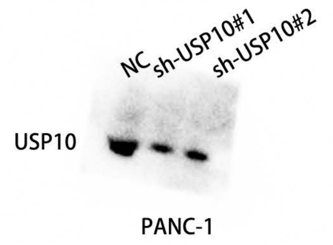

FIG6B right second

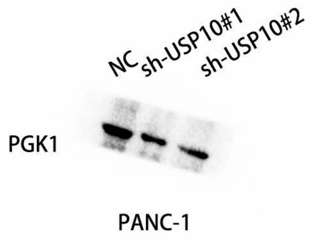

FIG6B right third

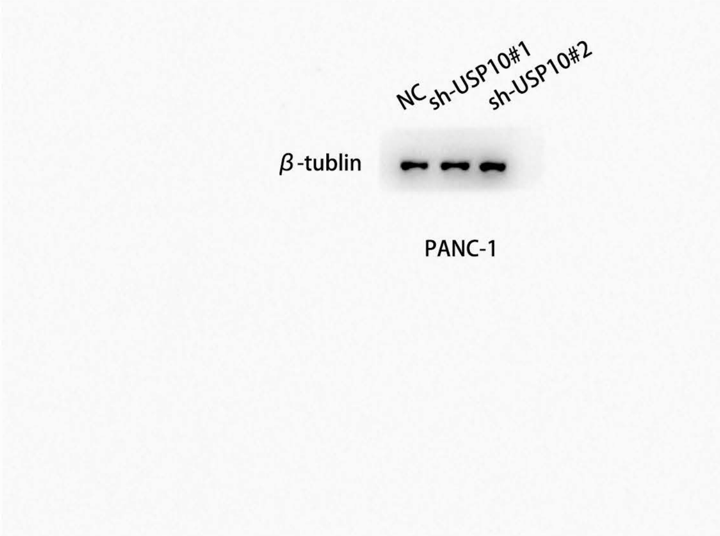

FIG6C left first

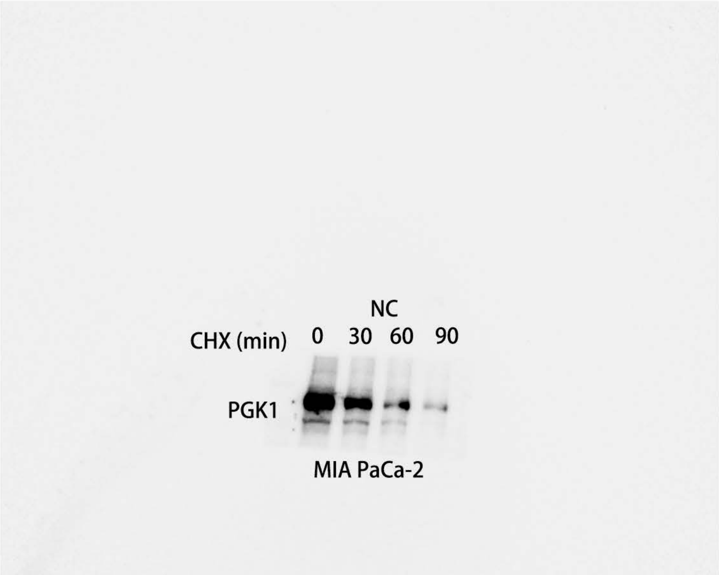

FIG6C left second

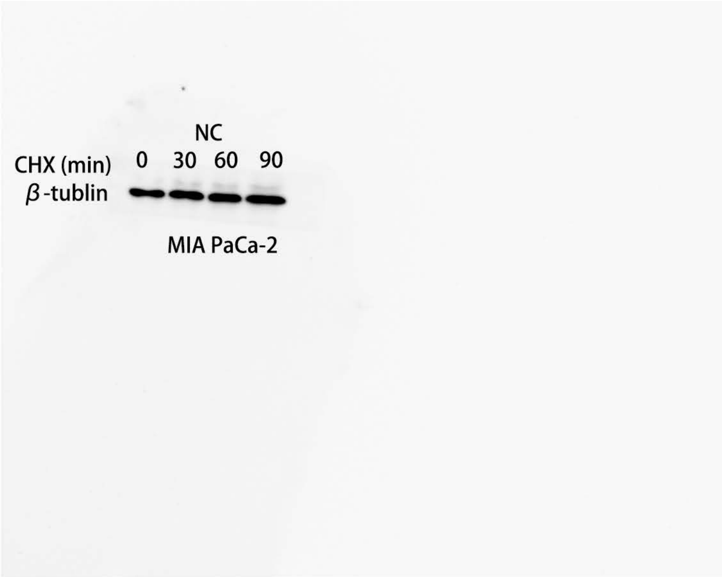

FIG6C left third

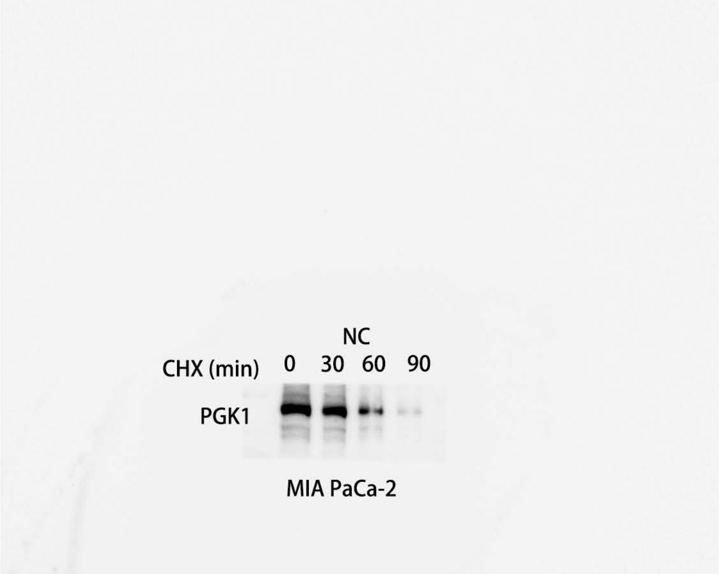

FIG6C left fourth

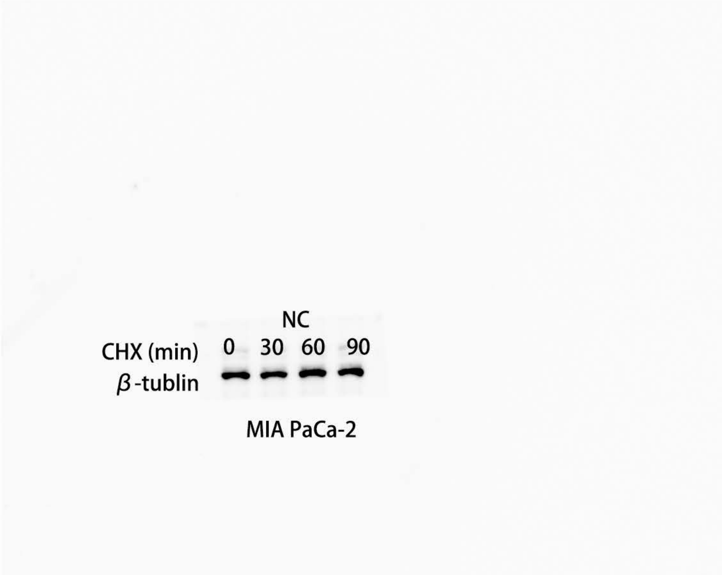

FIG6C right first

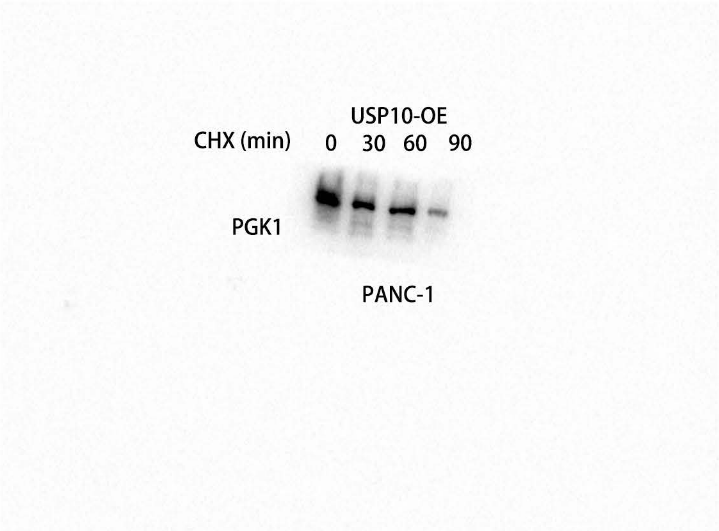

FIG6C right second

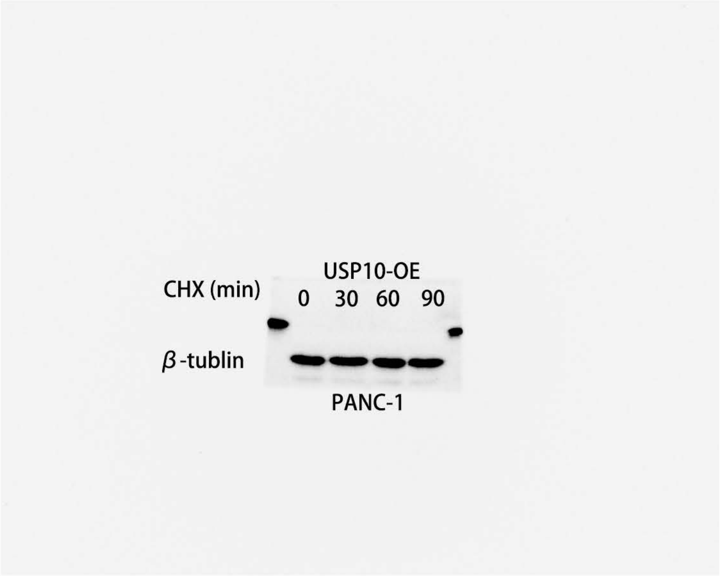

FIG6C right third

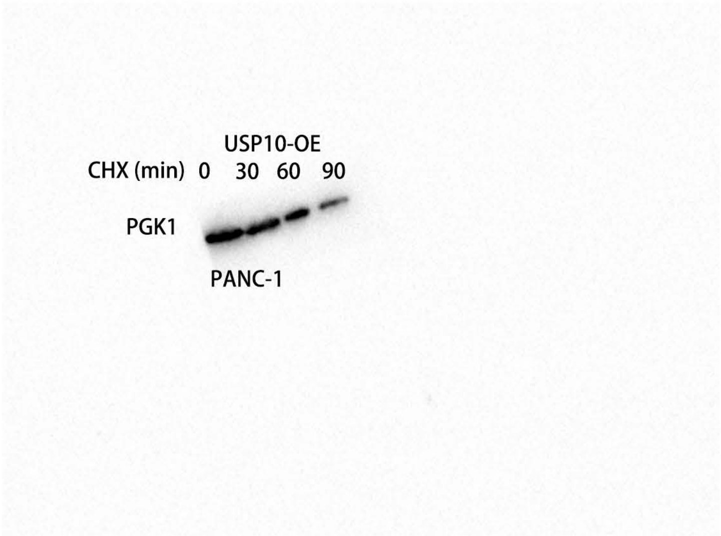

FIG6C right fourth

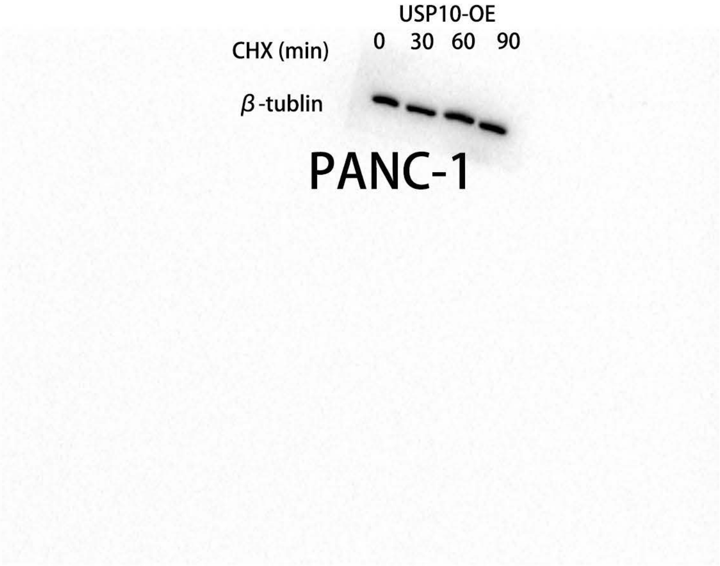

FIG6E first

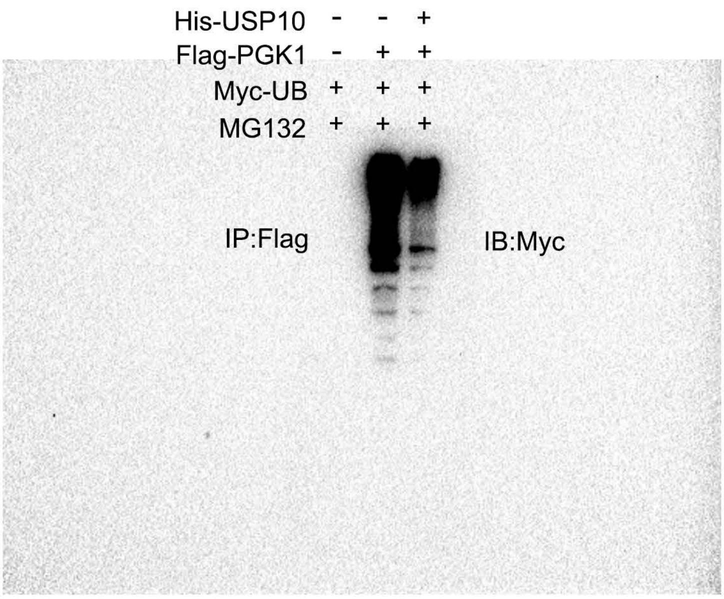

FIG6E second

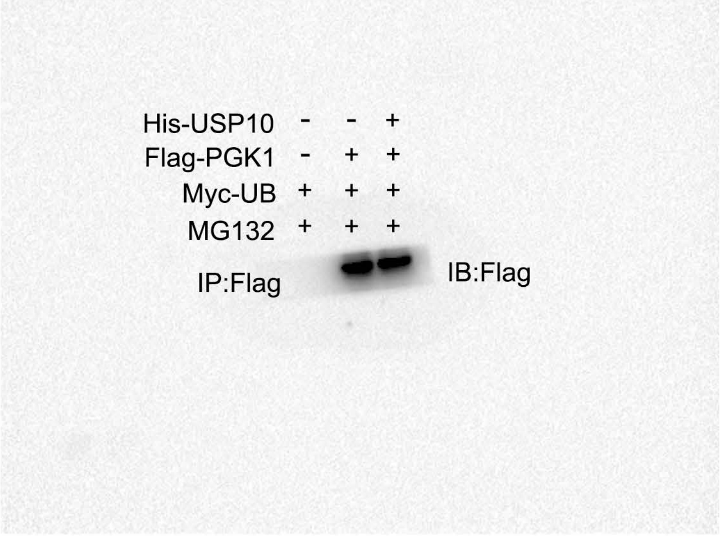

FIG6E third

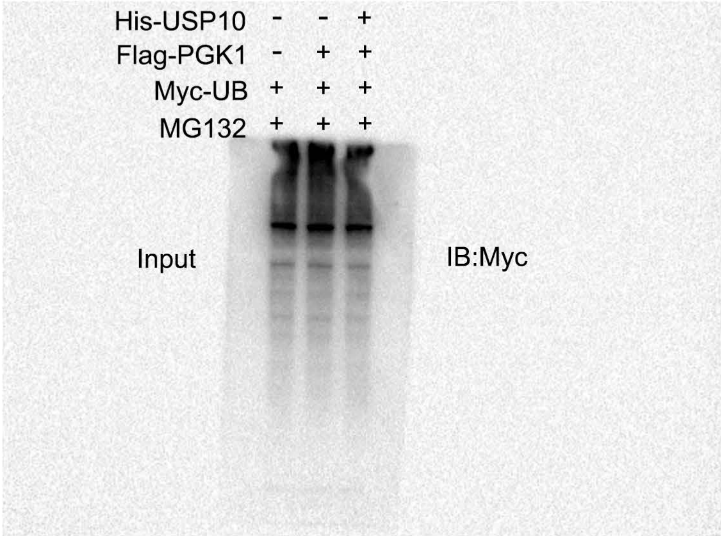

FIG6E fourth

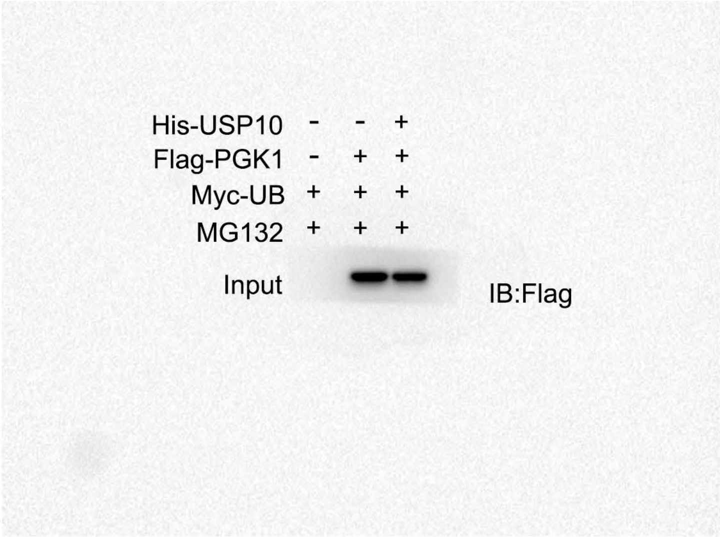

FIG6E fifth

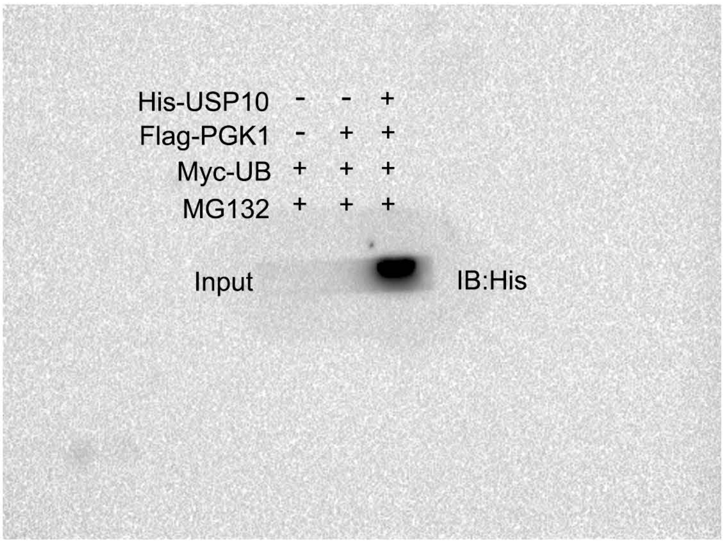

FIG6E sixth

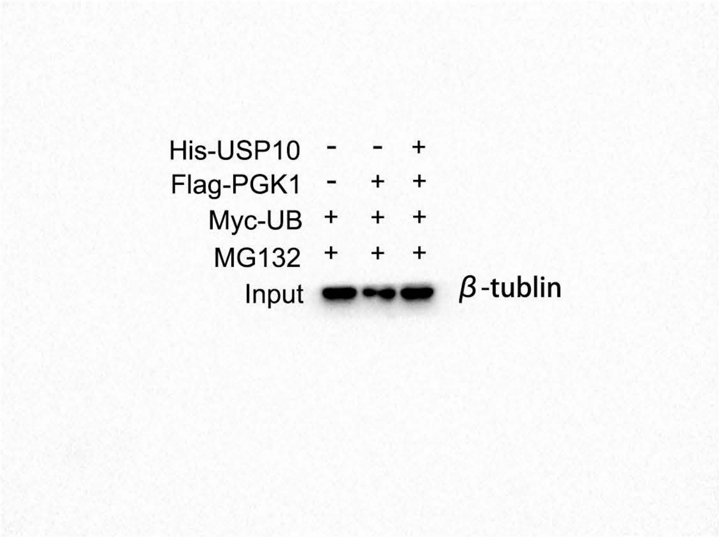

FIG6F left firt

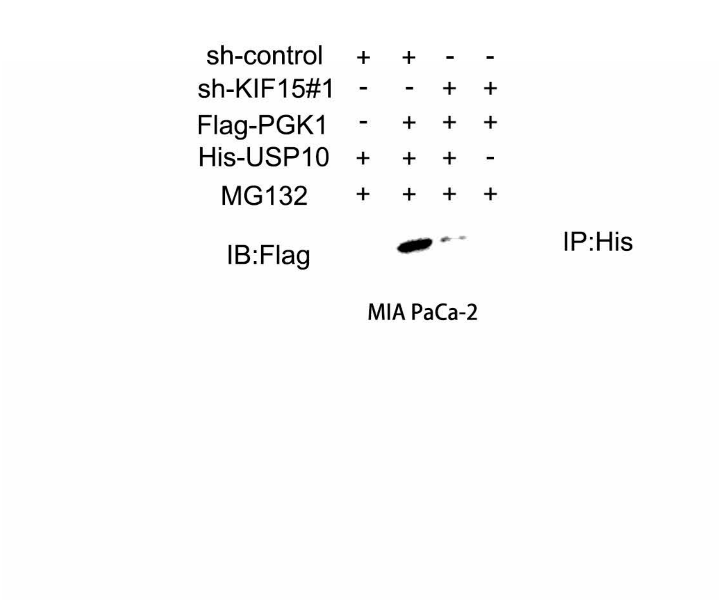

FIG6F left second

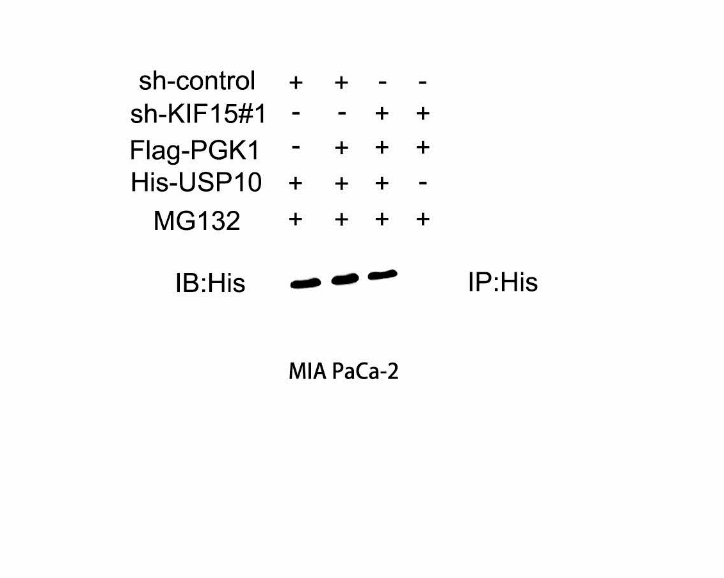

FIG6F left third

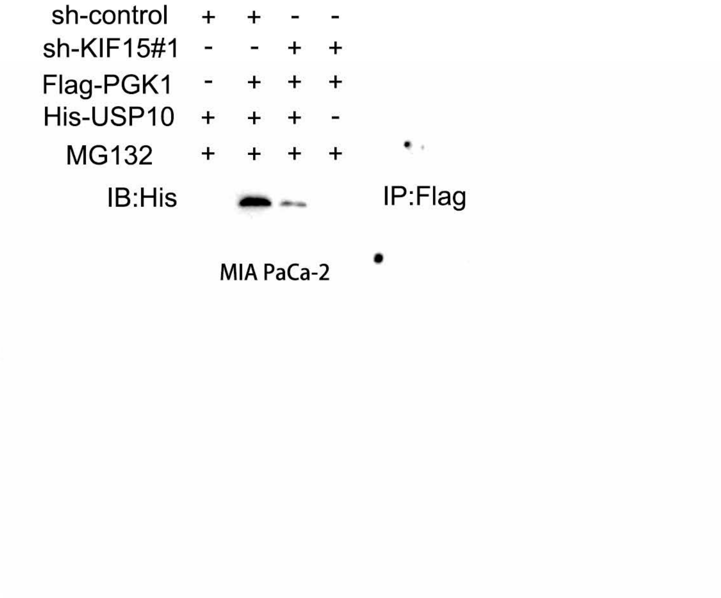

FIG6F left fourth

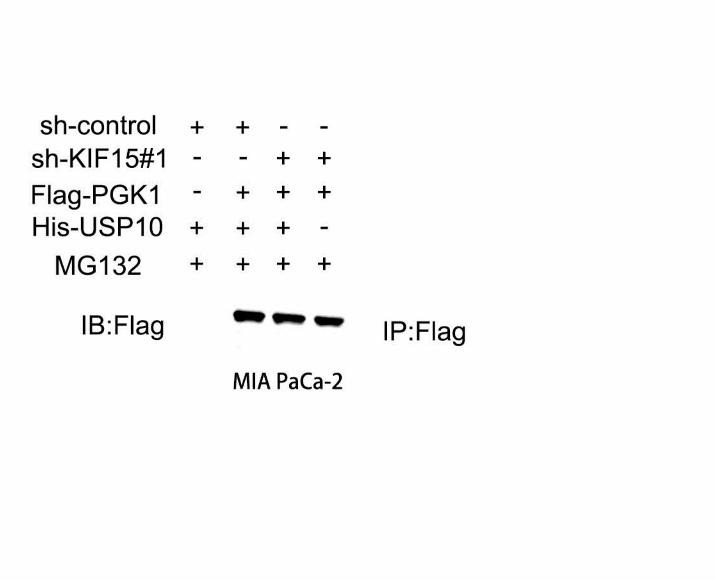

FIG6F left fifth

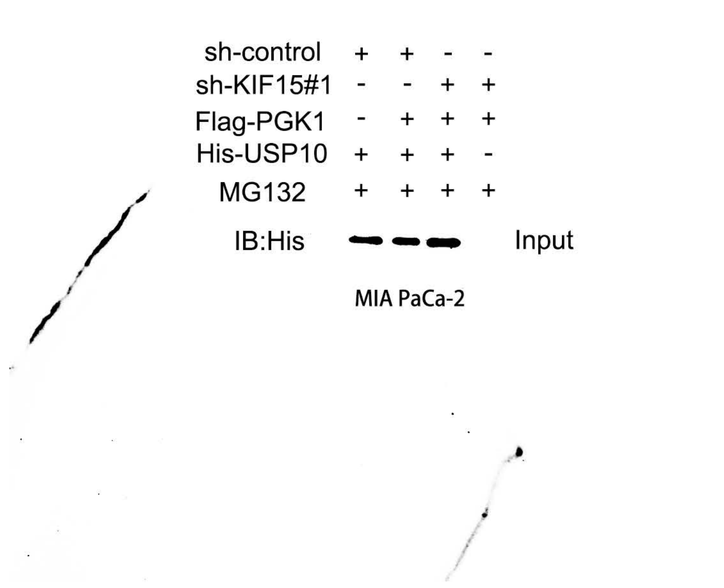

FIG6F left sixth

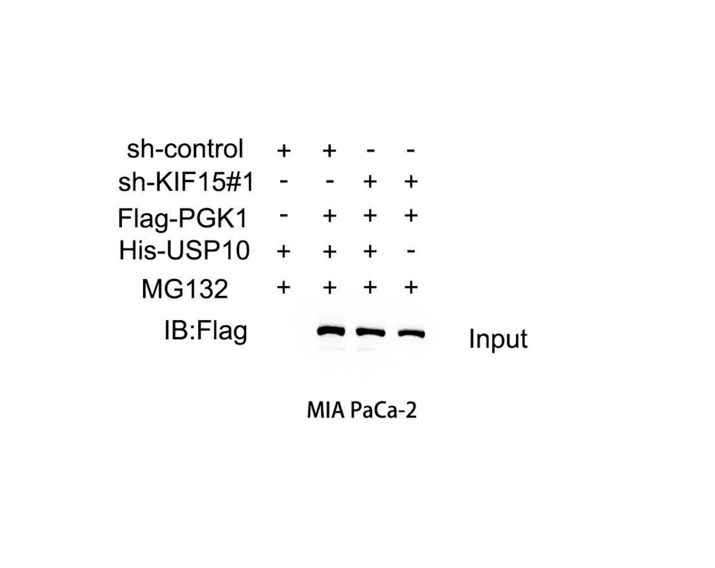

FIG6F left seventh

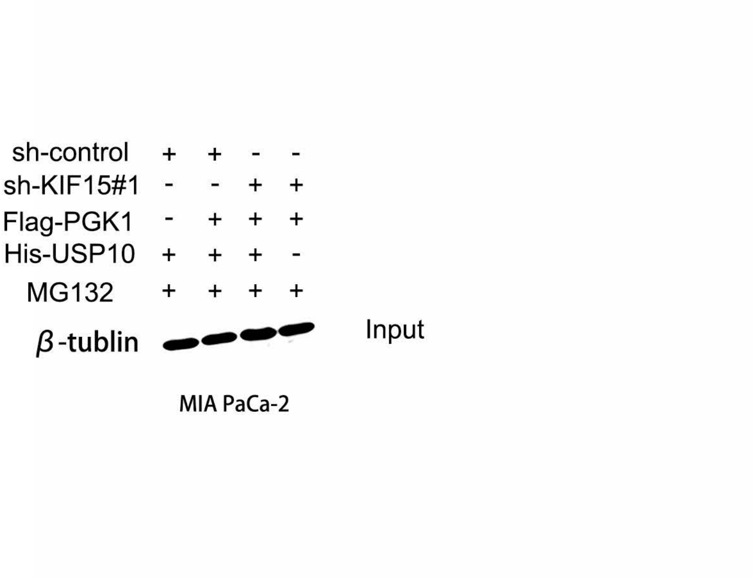

FIG6F right first

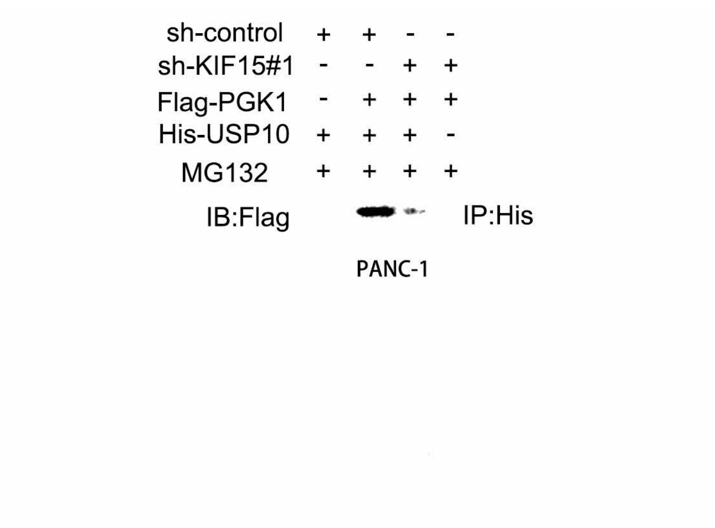

FIG6F right second

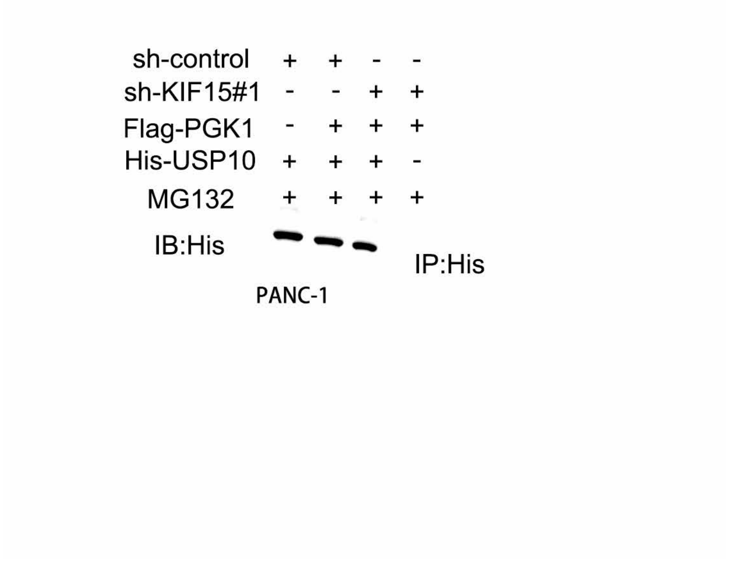

FIG6F right third

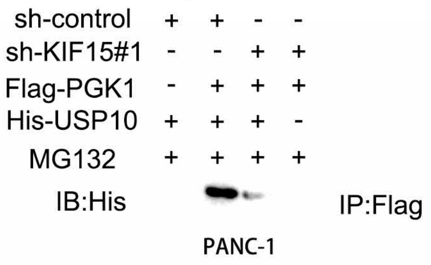

FIG6F right fourth

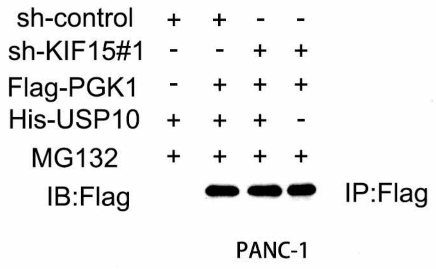

FIG6F right fifth

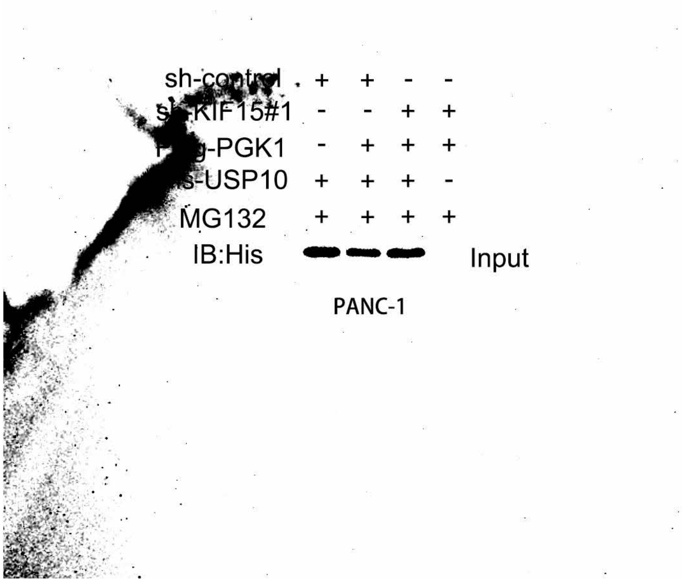

FIG6F right sixth

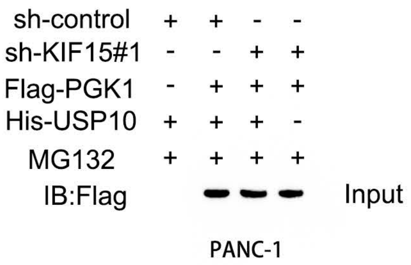

FIG6F right seventh

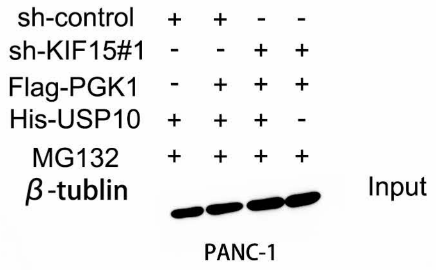

FIG6G first

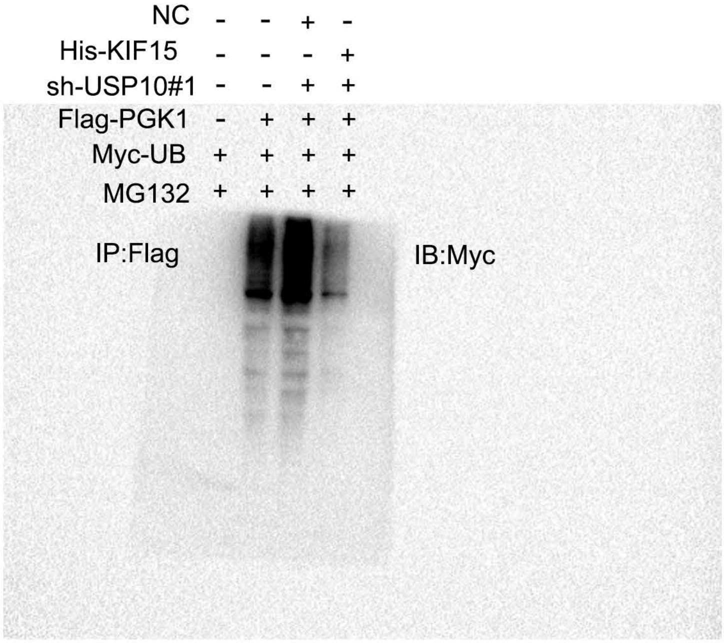

FIG6G second

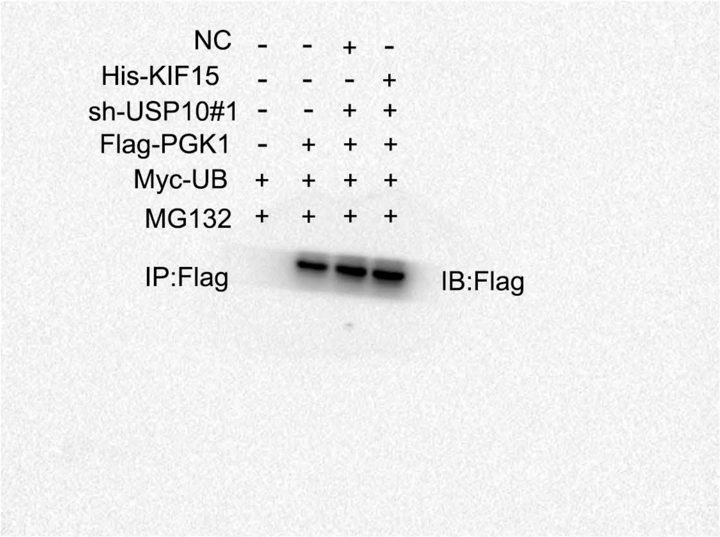

FIG6G third

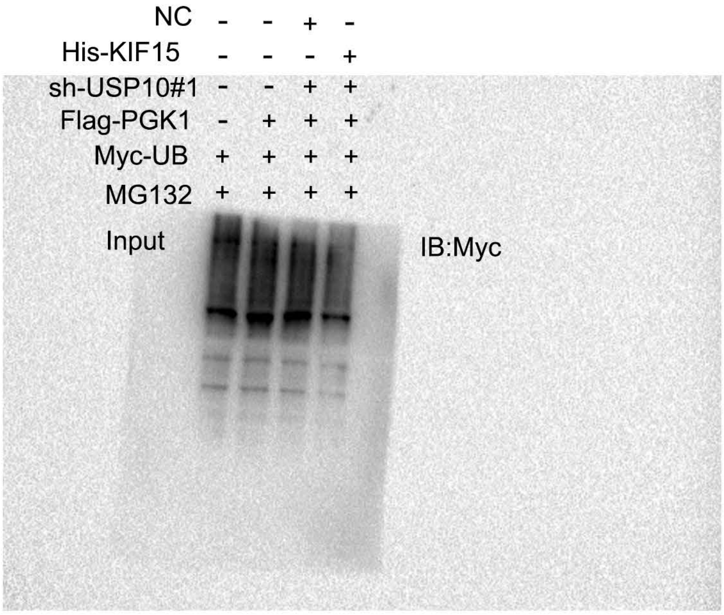

FIG6G fourth

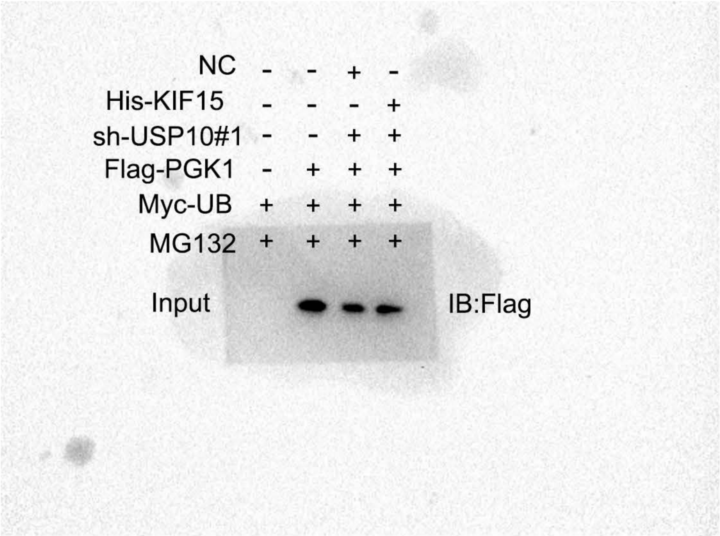

FIG6G fifth

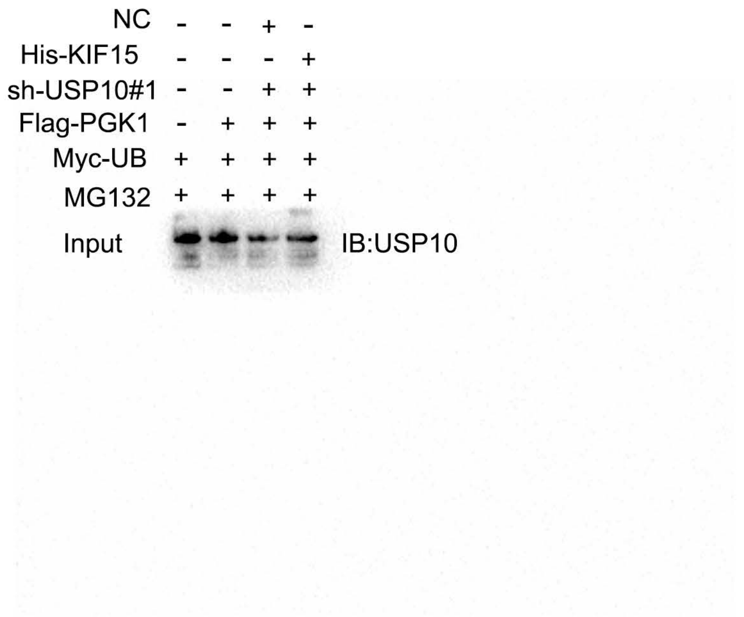

FIG6G sixth

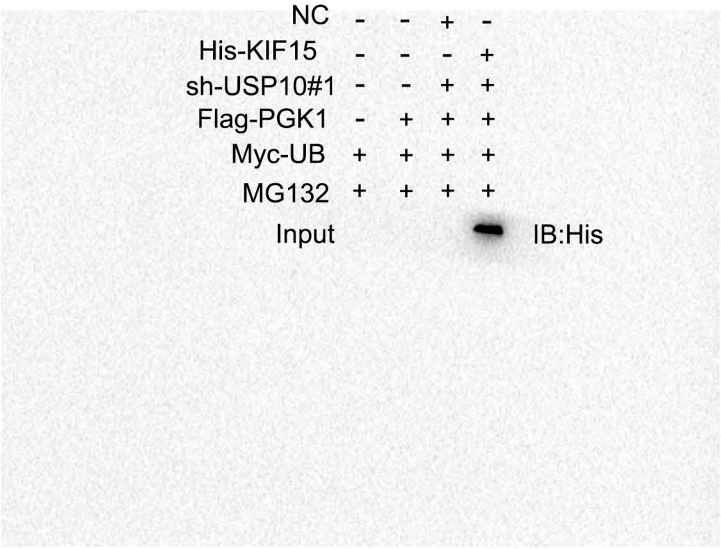

FIG6G seventh

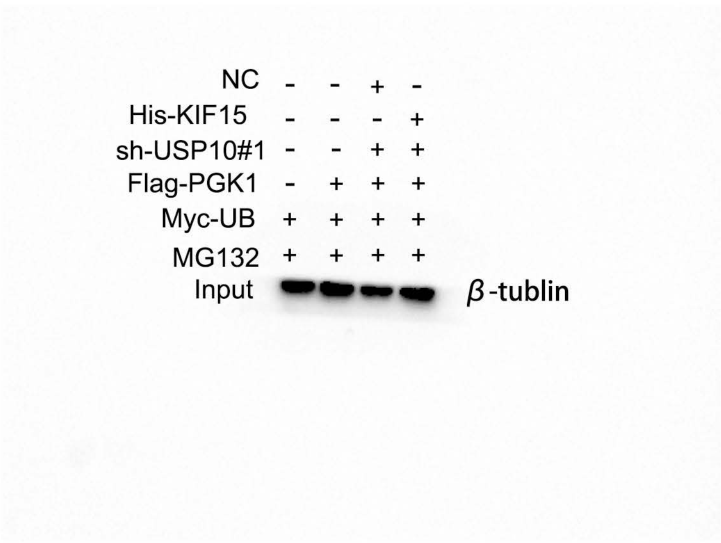

FIG6H first

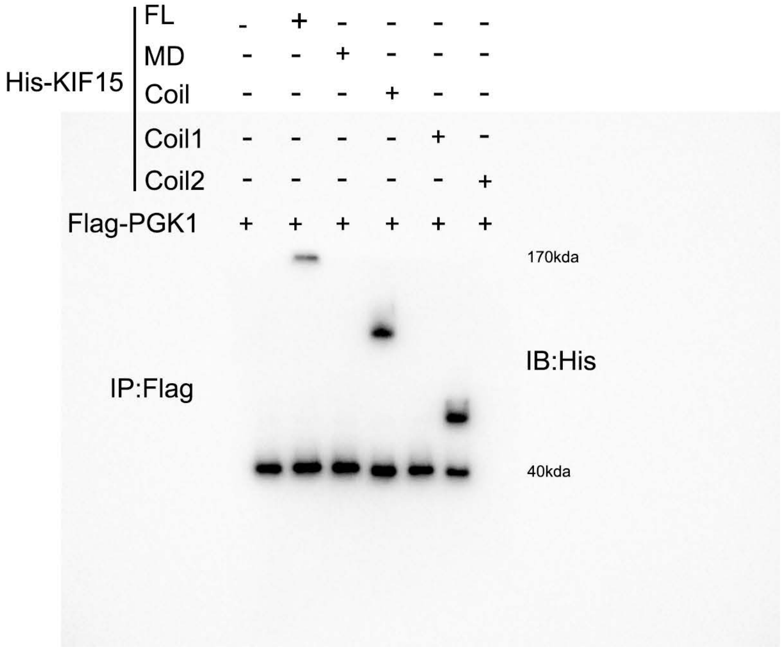

FIG6h second

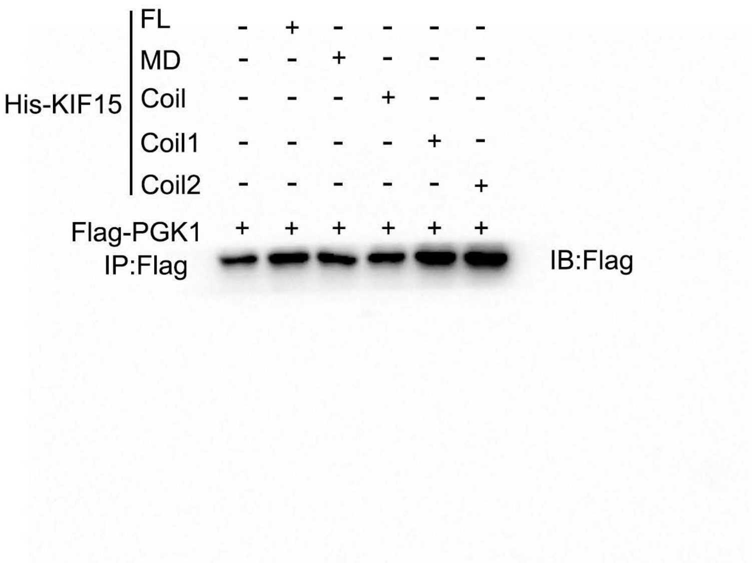

FIG6h third

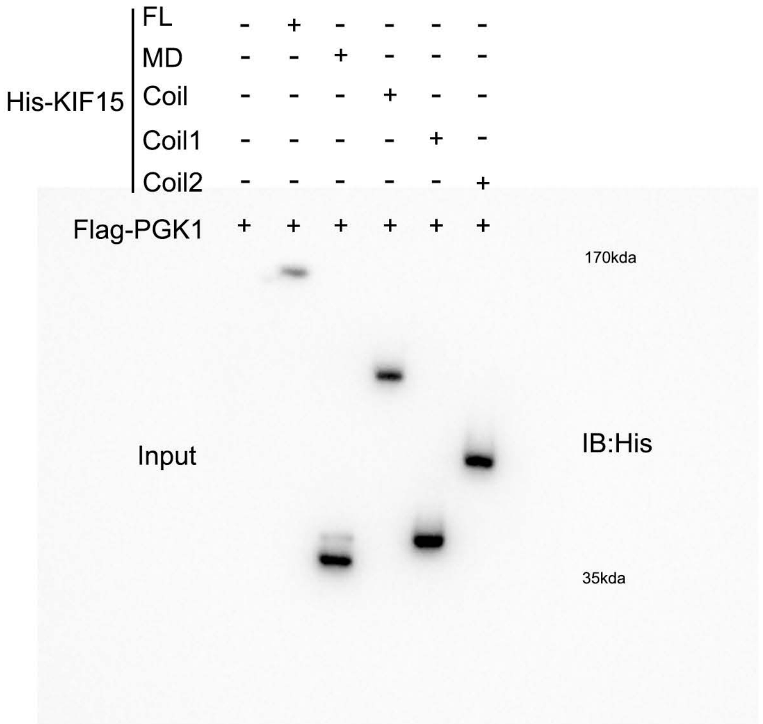

FIG6h fourth

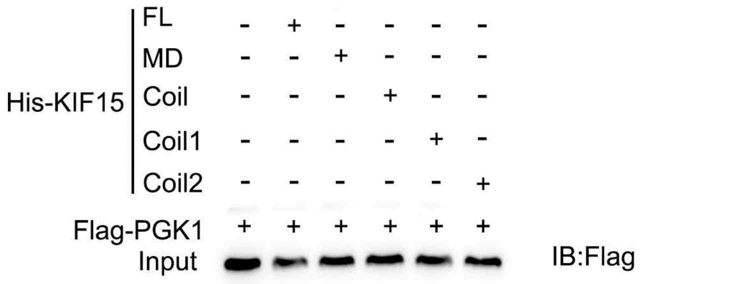

FIG6h fifth

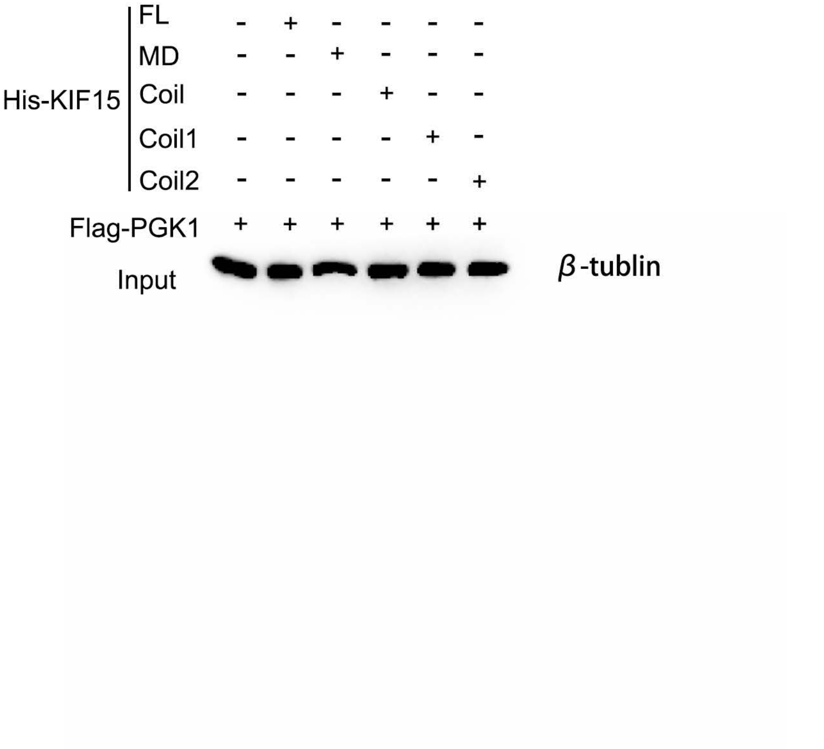

FIG6l first

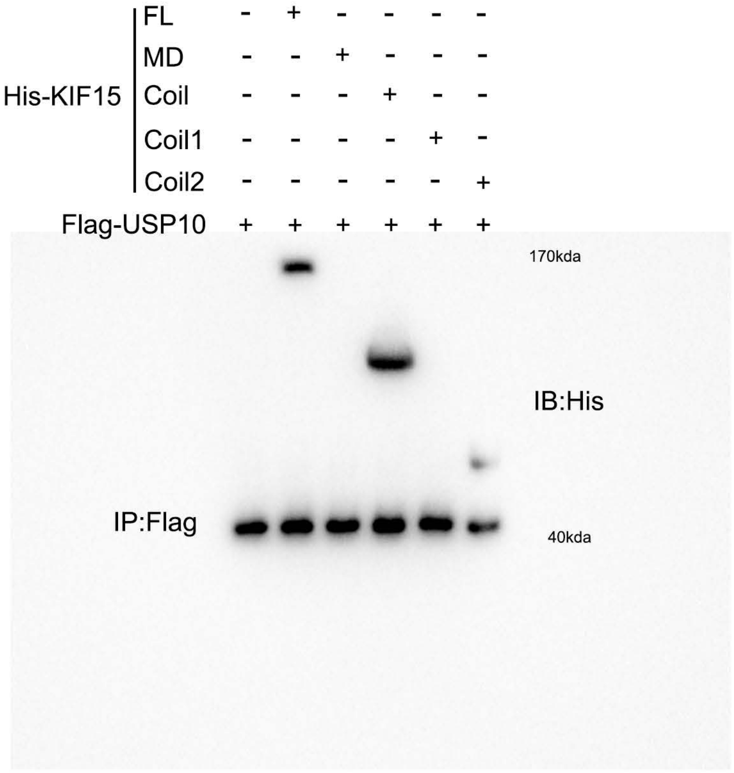

FIG6l second

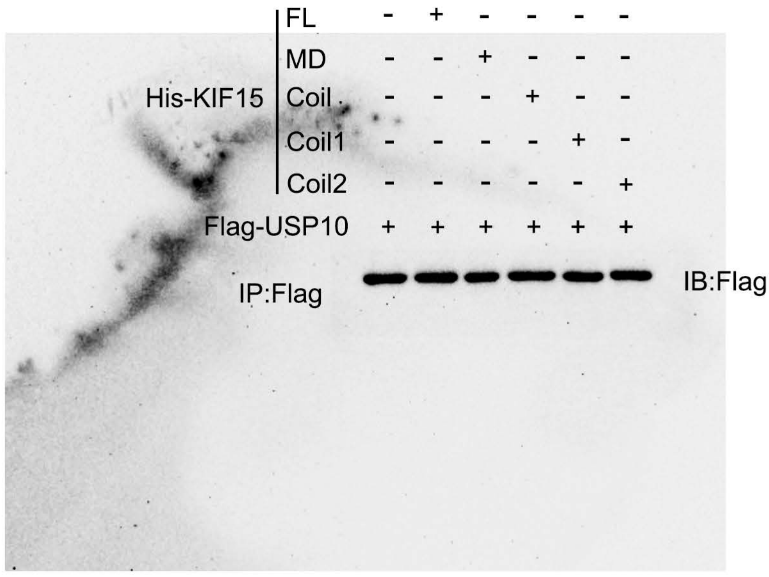

FIG6l third

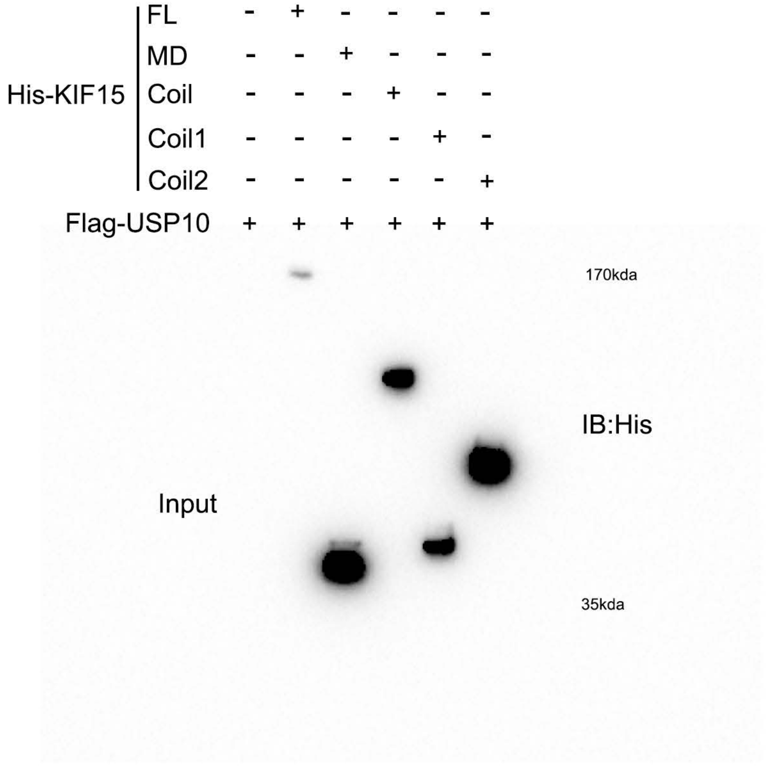

FIG6I fourth

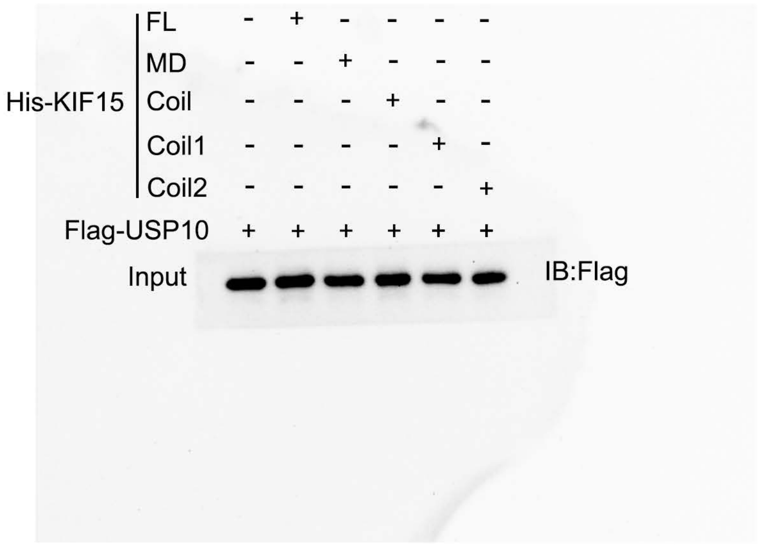

FIG6I fifth

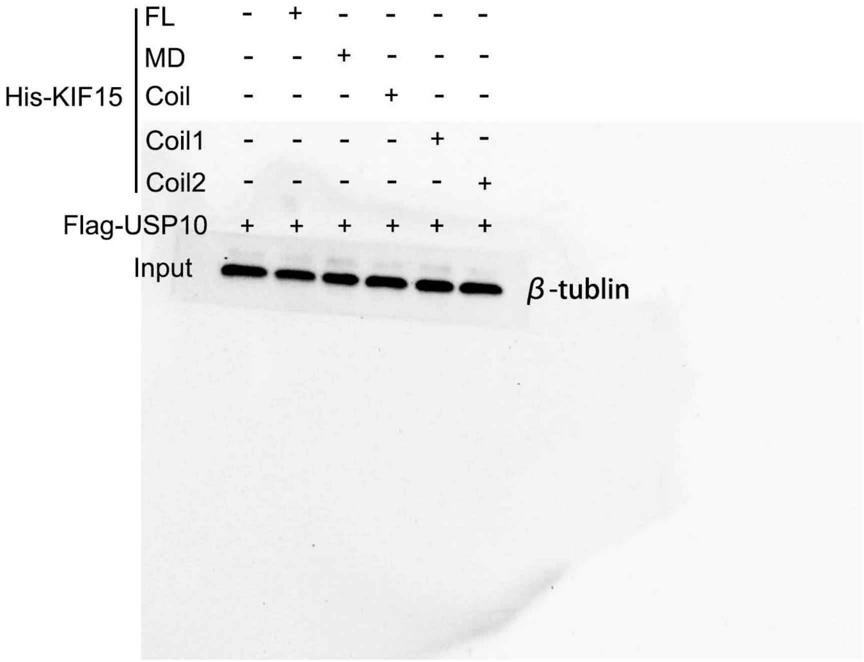

FIG7G MIA left first

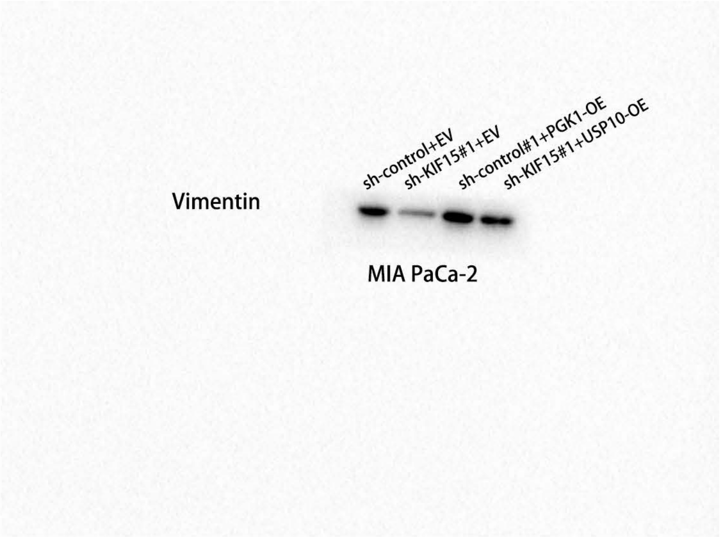

FIG7G MIA left second

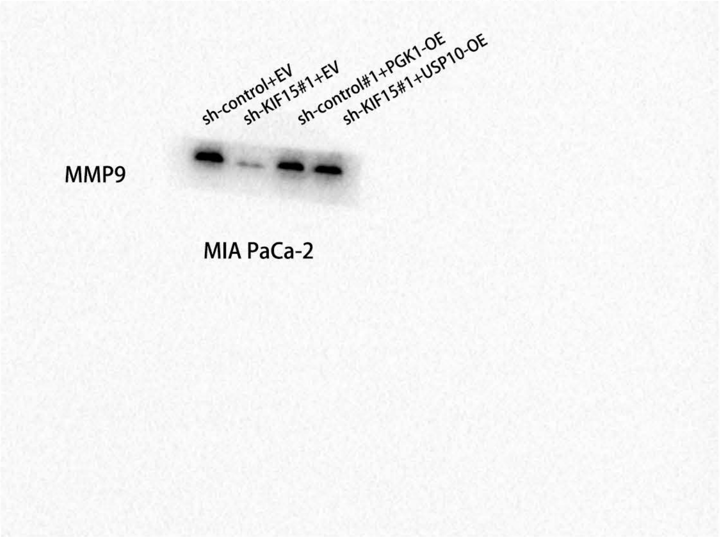

FIG7G MIA left third

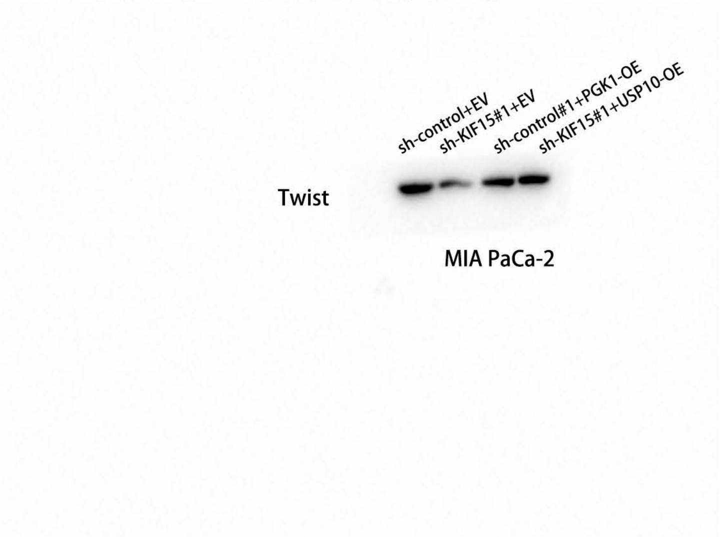

FIG6G mia left fourth

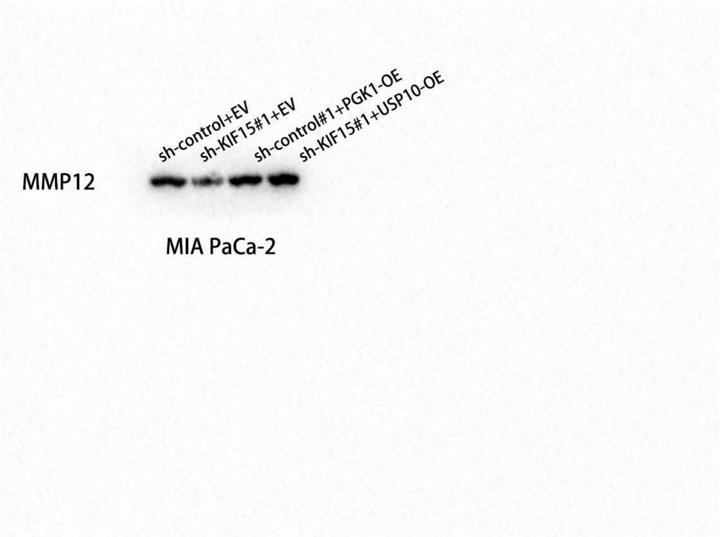

FIG6G mia left fifth

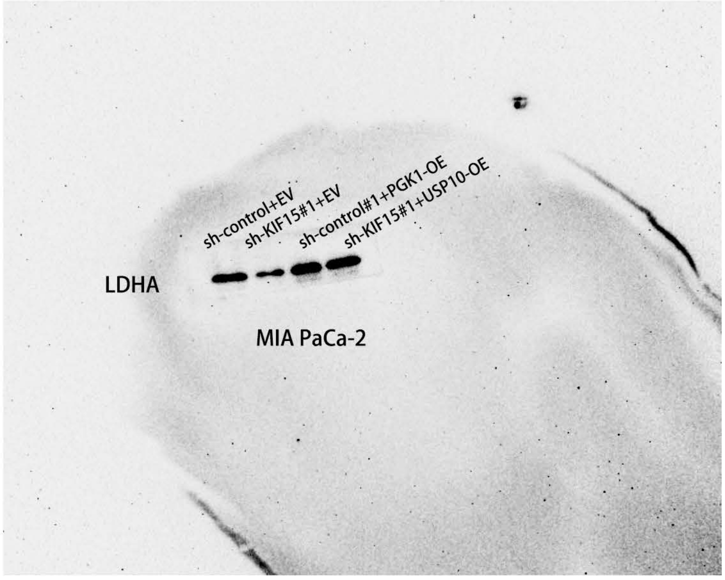

FIG6G mia right first

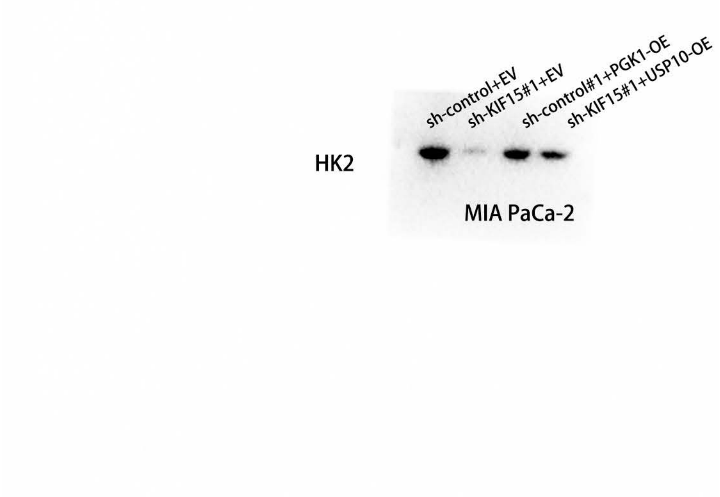

FIG6G mia right second

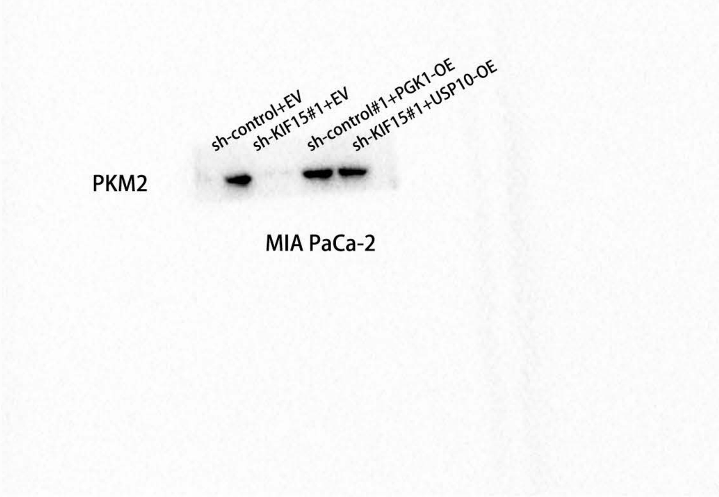

FIG6G mia right third

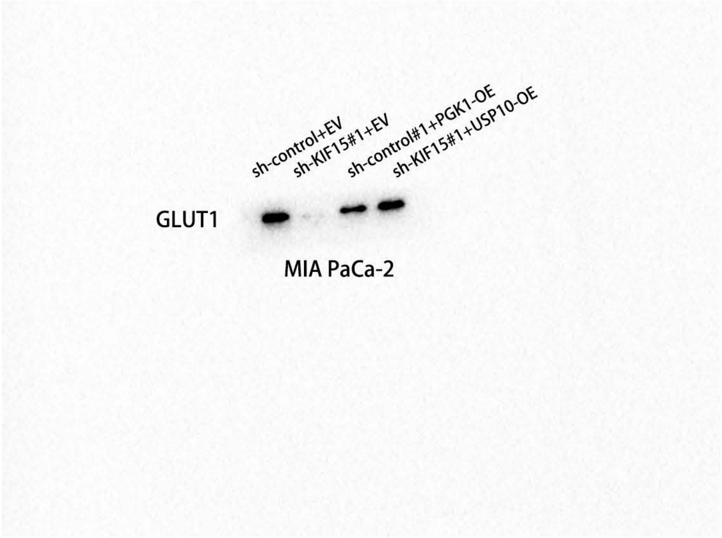

FIG6G mia right fourth

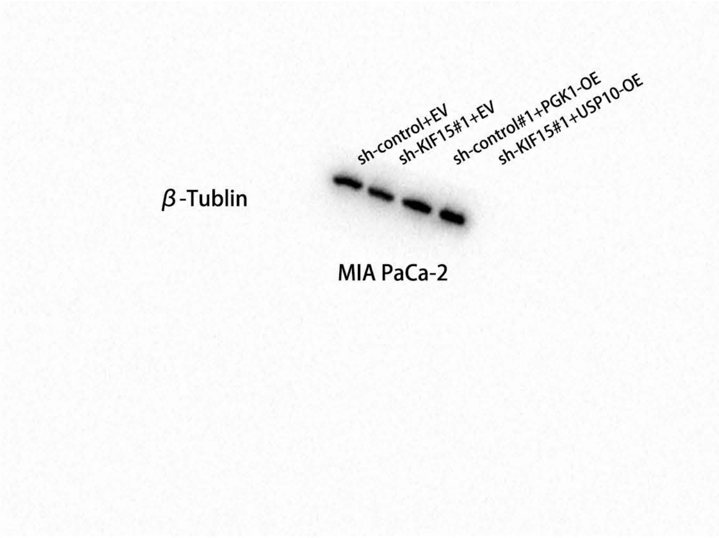

FIG7G panc left first

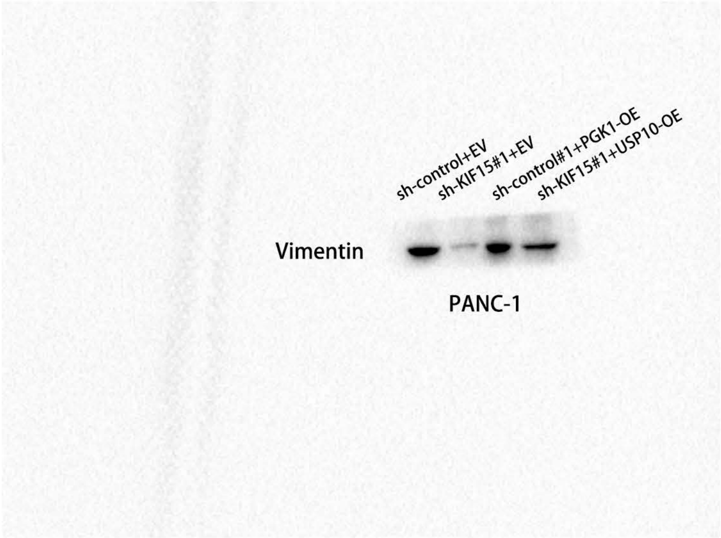

FIG7G panc left second

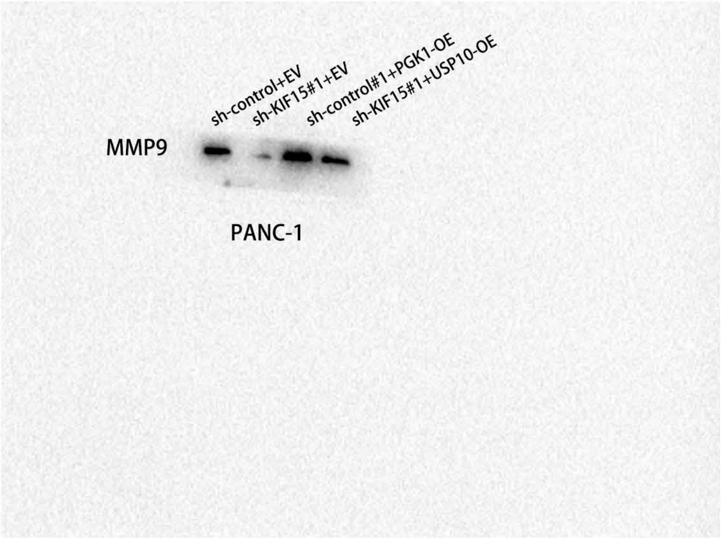

FIG7G panc left third

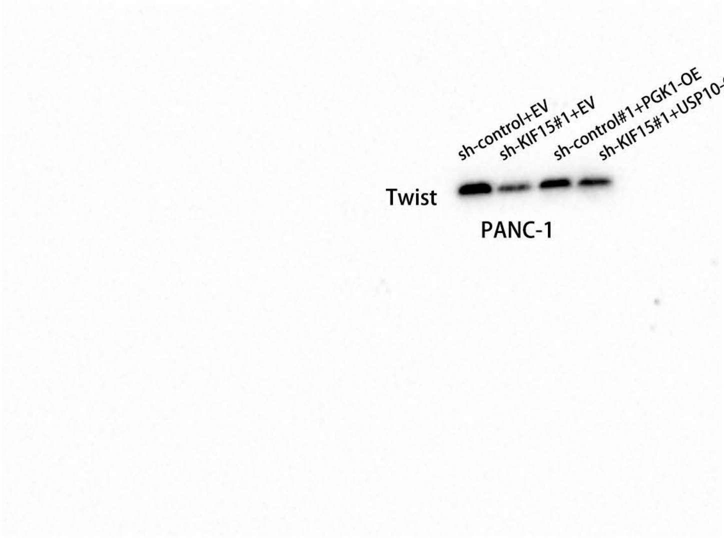

FIG7G panc left fourth

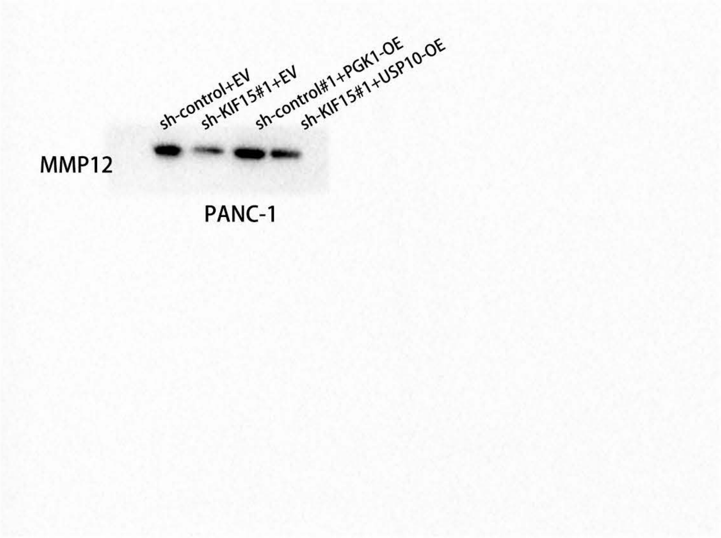

FIG7G panc left fifth

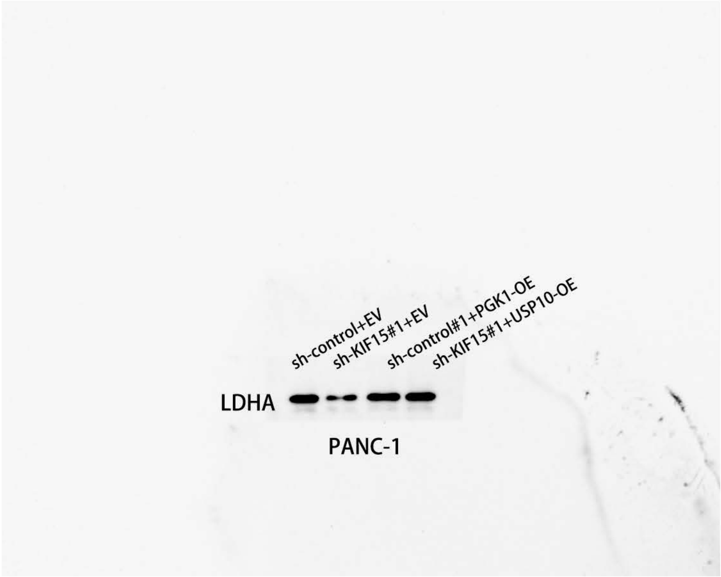

FIG7G panc right first

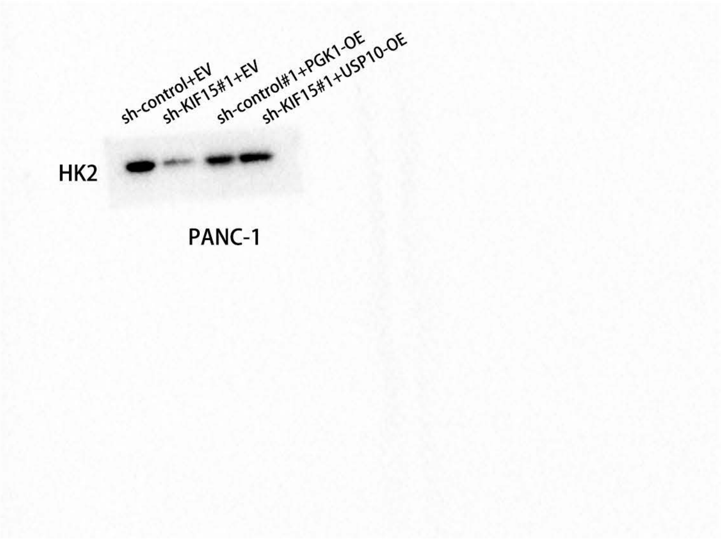

FIG7G panc right second

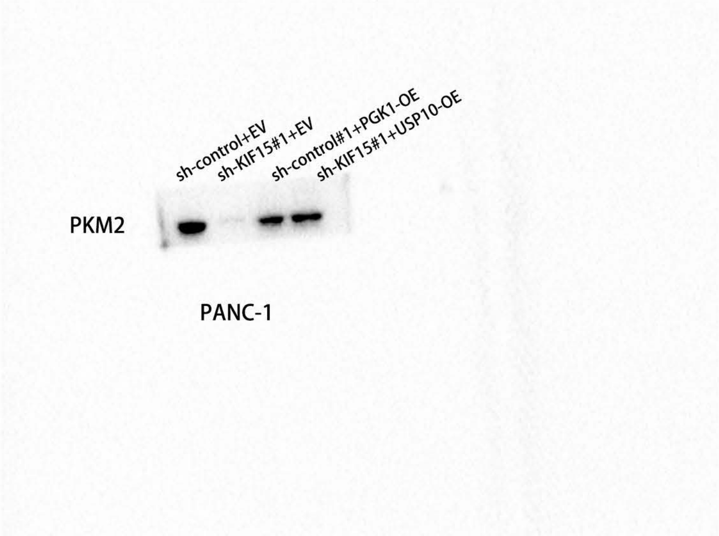

FIG7G panc right third

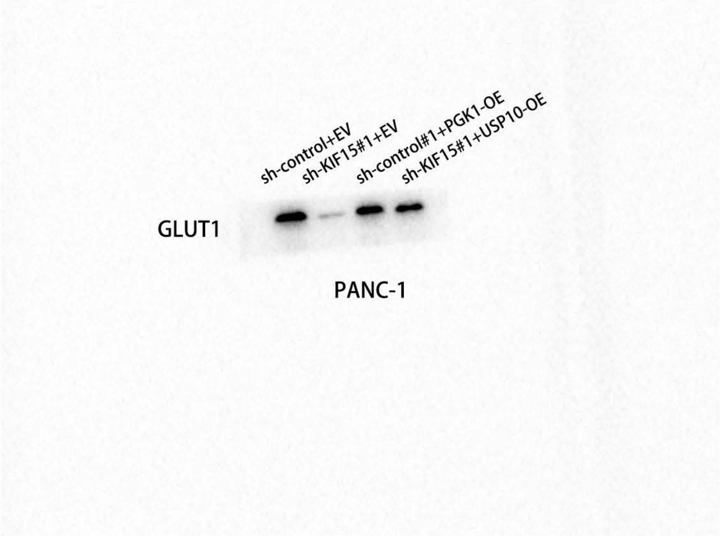

FIG7G panc right fourth

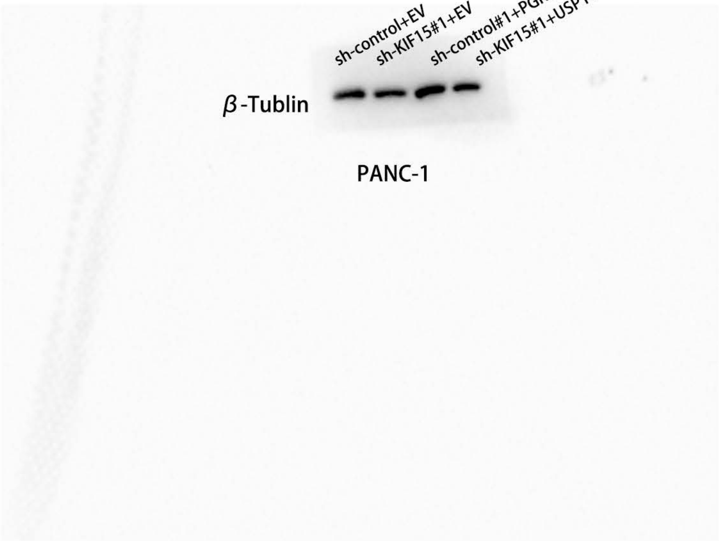

FIGS2 UP first

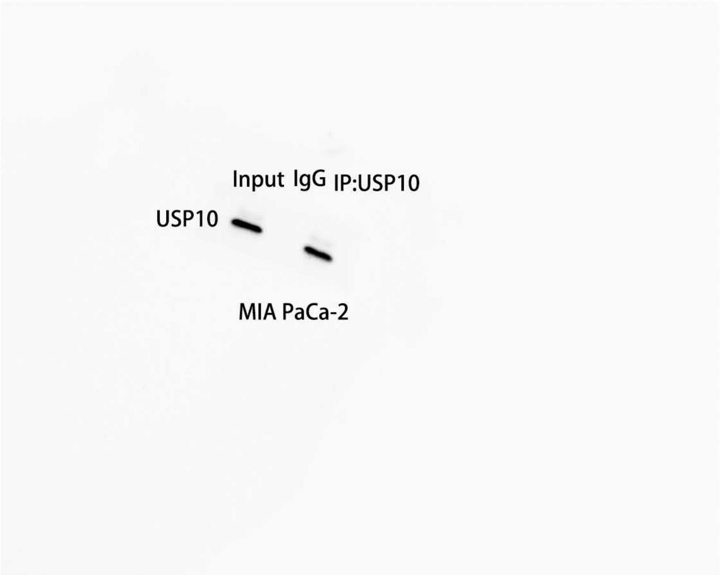

FIGS2 UP second

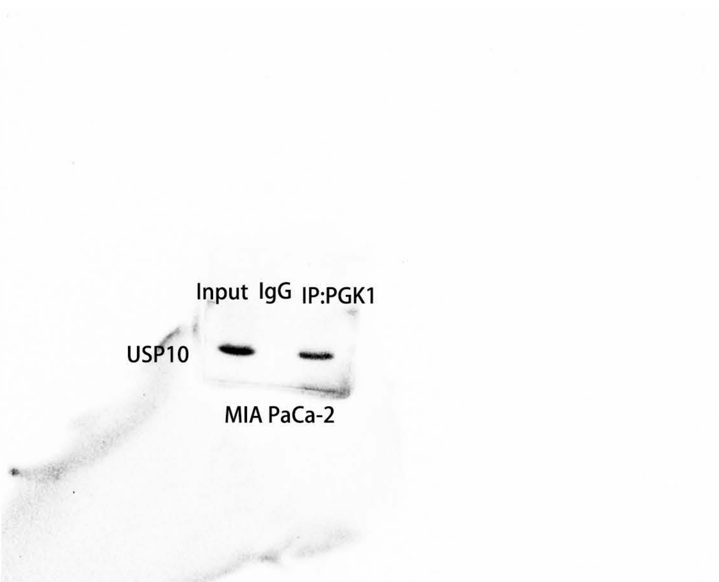

FIGS2 UP third

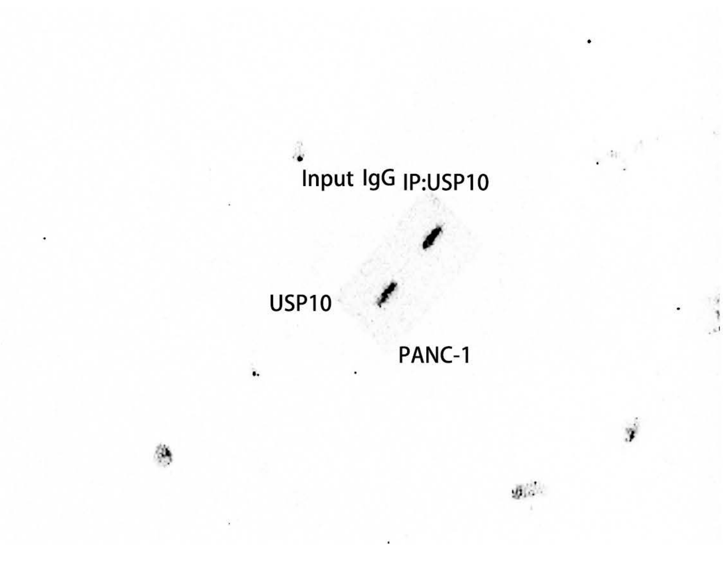

FIGS2 UP fourth

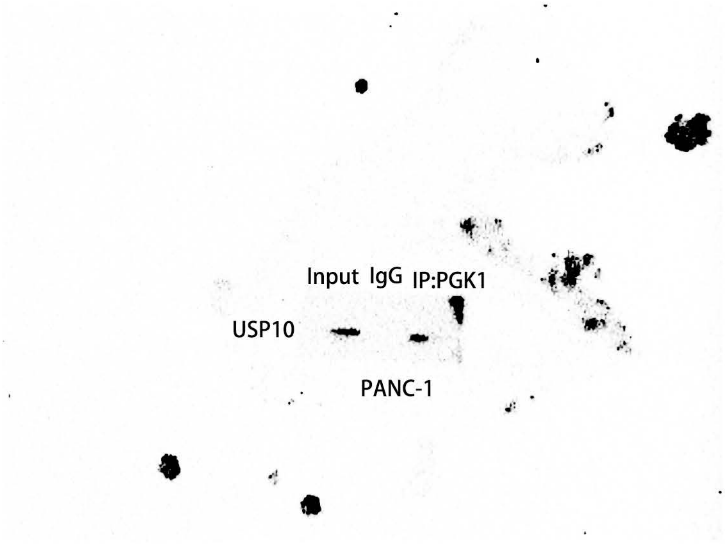

FIGS2 down first

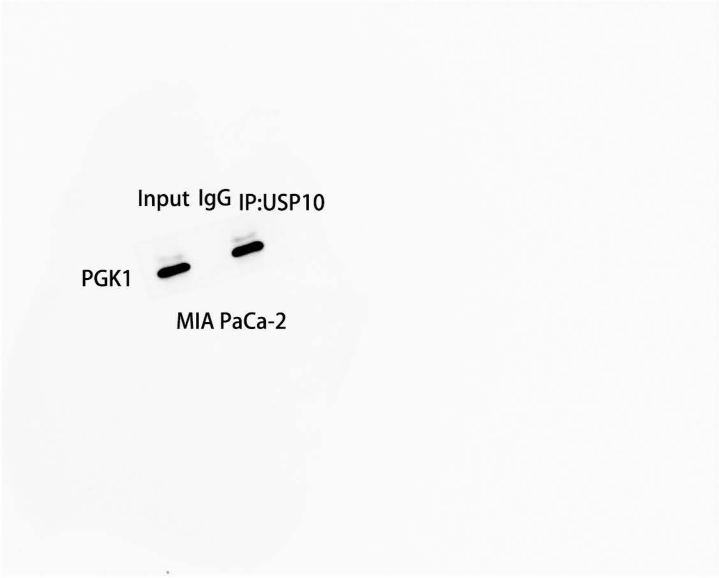

FIGS2 down second

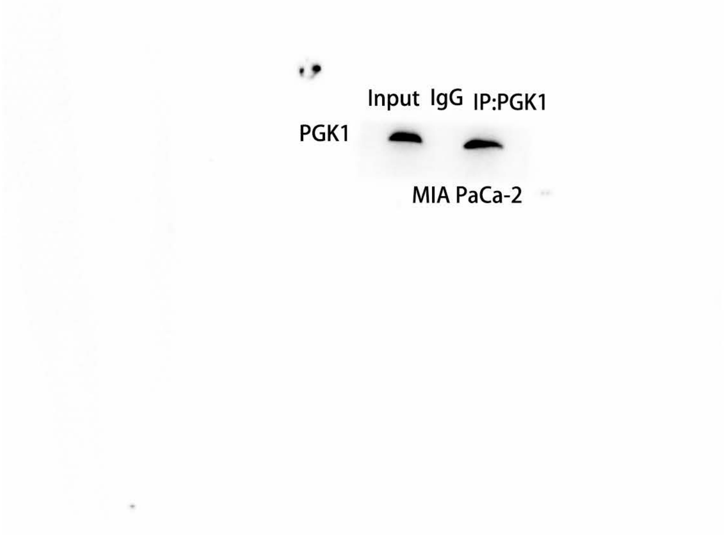

FIGS2 down third

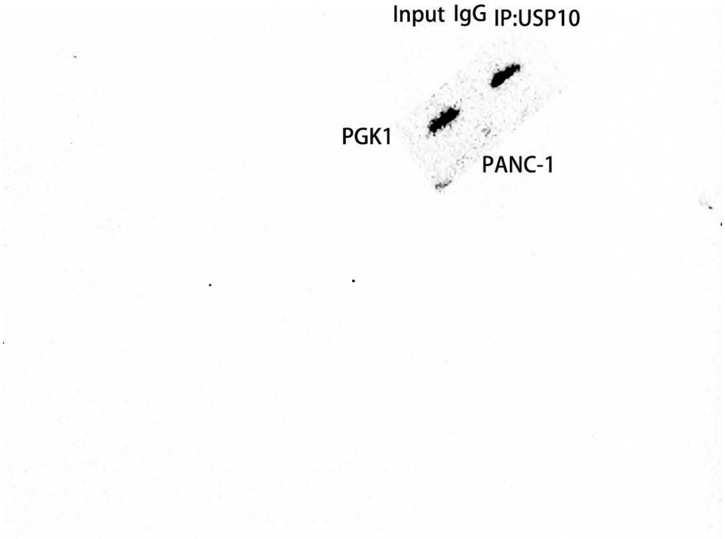

FIGS2 down fourth

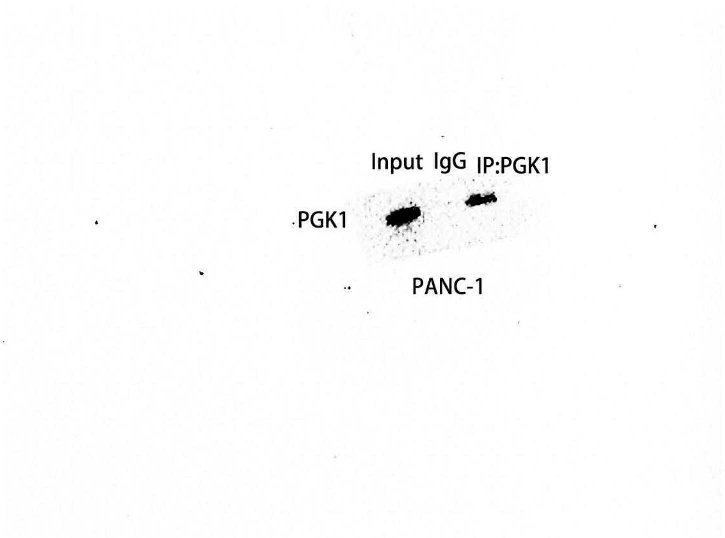

Supplement: Supplementary file 8 — Original Data File [file 41419_2023_5679_MOESM8_ESM.pdf]
